# Unravelling cell migration: defining movement from the cell surface

**DOI:** 10.1080/19336918.2022.2055520

**Published:** 2022-05-01

**Authors:** Francisco Merino-Casallo, Maria Jose Gomez-Benito, Silvia Hervas-Raluy, Jose Manuel Garcia-Aznar

**Affiliations:** a Multiscale in Mechanical and Biological Engineering (M2BE), Aragon Institute of Engineering Research (I3A), Zaragoza, Spain; b Department of Mechanical Engineering, University of Zaragoza, Zaragoza, Spain

**Keywords:** Cell migration, cell mechanics, extracellular matrix

## Abstract

Cell motility is essential for life and development. Unfortunately, cell migration is also linked to several pathological processes, such as cancer metastasis. Cells’ ability to migrate relies on many actors. Cells change their migratory strategy based on their phenotype and the properties of the surrounding microenvironment. Cell migration is, therefore, an extremely complex phenomenon. Researchers have investigated cell motility for more than a century. Recent discoveries have uncovered some of the mysteries associated with the mechanisms involved in cell migration, such as intracellular signaling and cell mechanics. These findings involve different players, including transmembrane receptors, adhesive complexes, cytoskeletal components , the nucleus, and the extracellular matrix. This review aims to give a global overview of our current understanding of cell migration.

## Introduction

Cell migration is fundamental for life and development. Key physiological processes of multicellular organisms depend on cell migration, from embryonic development to the more specific bone formation and angiogenesis. Cells’ ability to migrate is also critical during tissue repair and the inflammatory and immune responses. But cell migration is associated with disease development too, including some of the leading causes of death, such as cancer metastasis. A comprehensive understanding of this biological process is therefore essential.

Cell migration is an extremely complex phenomenon involving a wide variety of biological processes. Factors such as cell phenotype or the properties of the surrounding extracellular matrix (ECM) regulate the activation of some of these processes. Note that cells produce the ECM to surround themselves with a scaffolding structure [1,3]. Therefore, cells can modulate the properties of their surrounding ECM. Different external cues, including chemical and biophysical stimuli from their microenvironment, influence cell migration [4], promoting cell invasion, immune cell motility, and facilitating tumor cell dissemination [5–8]. Notably, cells’ phenotype, as well as their microenvironment, determine if and how cells migrate [9–12].

**Figure 1.**
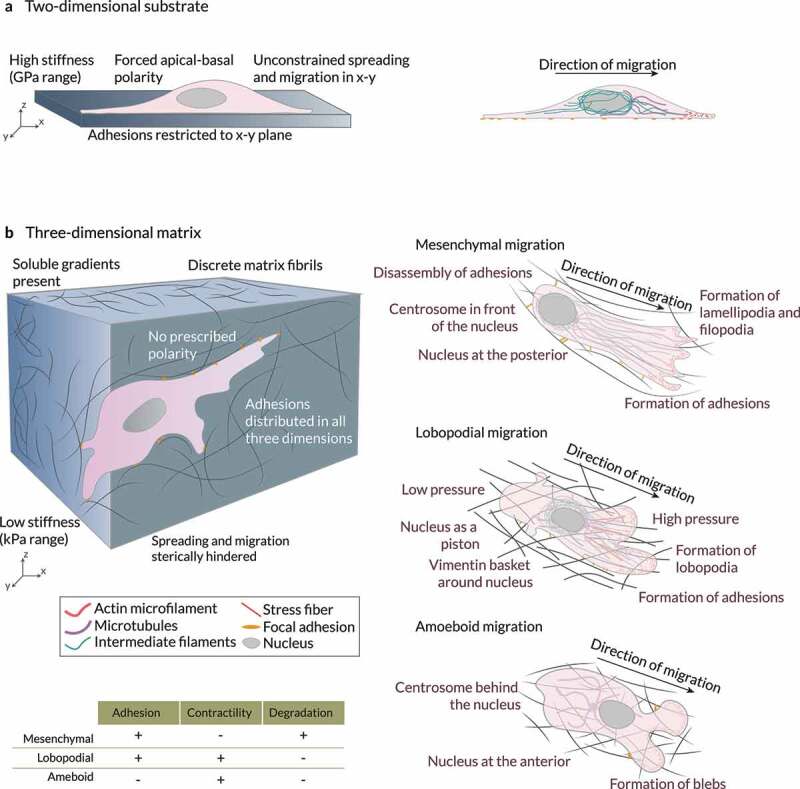
Cells in 2D vs 3D Cells in (a) 2D and (b) 3D microenvironments interact differently with their surroundings. Three modes of 3D migration have been identified so far: mesenchymal, amoeboid, and lobopodial migration. In mesenchymal migration, cells attach very strongly to the extracellular matrix through mature stress fiber-linked focal adhesions. These cells also exhibit a high matrix-degrading activity. The centrosome is in front of the nucleus and the cytoskeletal networks are polarized in the direction of migration. In contrast, amoeboid migration involves very few adhesions and low protease activity. Cells migrate through the formation of contraction-based blebs or use actin-driven protrusions to glide on the substrate. The centrosome is usually behind the nucleus during amoeboid migration. Lastly, during lobopodial migration, tightly adherent cells use actomyosin contractility, hydrostatic pressure, and nuclear pistoning to form bleb-like blunt protrusions called lobopodia. These cells exhibit very low protease activity. Adapted from Refs. [39,167].

More than a century of research in the field [13–15] has allowed us to understand many of the intricacies of cell migration. However, because of its inherent complexity, plenty of unanswered questions still need to be addressed. Besides, much of what we know about cell migration (and of cell biology, for that matter) is based on cells cultured on Petri dishes or rigid flat sheets of plastic. Still, many are the differences between these two-dimensional (2D) substrates and the more physiological three-dimensional (3D) matrices (Figure 1). For one, soluble gradients are absent on plated cultures, whereas they may be present in 3D. While an apical-basal polarity is forced on 2D substrates, there is no prescribed polarity in 3D environments. Instead of the high stiffnesses (GPa range) associated with plated cultures, the stiffness of gels in 3D is in the lower kPa range. Also, 3D matrices are more pliable than 2D substrates. As a result, cells can alter ECM compliance more easily in 3D domains. Cells also behave differently within 3D matrices than on 2D substrates (Figure 1) [16–18], including during migration [4,19,20]. Although spreading and migration are unconstrained on the *x–y* plane on flat surfaces, they may be sterically hindered in 3D. Cells in 3D environments adopt a thinner and more elongated shape. They also follow a more persistent and direct trajectory than those on 2D surfaces. Adhesions are restricted to the *x–y* plane in 2D substrates but are distributed in all three dimensions in these gels. Nuclear positioning is much more complex for cells migrating within 3D domains [21]. Another source of complexity in the study of cell migration is that its regulation depends on the biochemical and biophysical features of the pericellular space [22]. Therefore, cells must integrate concurrent, potentially cooperative, or opposing inputs in their decision-making process [23–26]. These external cues can modulate cellular properties and events, from cell shape and polarity to cell–cell and cell–matrix interactions. Likewise, cells adjust their trajectory, speed, and mode of migration accordingly (Figure 1) [25,27]. Even modest variations in the biochemical or biophysical stimuli can dramatically impact cells’ migratory phenotype [28]. Thus, we still need to fill in some gaps in our knowledge of how cells (i) probe the surrounding environment, (ii) integrate these cues, as well as (iii) adapt and respond to them.

Replicating scenarios closer to *in vivo* conditions, though, is a challenging endeavor [18,29–31]. For instance, if we focus on the mechanical response of *in vivo* environments, they have been identified as viscoelastic [32,33] (they present properties observed in solids and fluids) and exhibit stress relaxation [34]. Interestingly, the impact of stress relaxation speed on 3D cell migration may be modulated by the material’s steric hindrance [35]. Still, hydrogels used as synthetic substrates for 3D culture and tissue engineering *in vitro* are typically elastic. Measuring some features of these 3D matrices with the current technologies may be extremely difficult or even impossible [17 33,36–38]. Besides, in 3D domains, the underlying conditions must be more tightly controlled [22,39–41]. Hence, studying cell migration under more physiologically relevant scenarios is not an easy task.

This review aims to give a global overview of our current understanding of cell migration and the different processes and players involved. We will start at the cell surface, where transmembrane receptors enable cells to sense external stimuli from their surroundings. Then, we will focus on the mechanics of cell motility. Different adhesive complexes, also located at the surface, allow cells to interact with one another and with the ECM. By binding to and interacting with all these players from the plasma membrane, the cytoskeleton can receive, process, and respond to signals from the outside. The cytoskeleton is also coupled to the nucleus. As a result, cells nuclei can adapt and react to the relayed signals initiated by external stimuli. Next, we will review different approaches to model some aspects of cell motility. Finally, we will discuss some of the current and future challenges for the research community. Note there are many excellent reviews about specific players or events associated with cell migration (e.g. [8,9,17,25,42]).

## Probe of biochemical stimuli

Cells can change their migratory patterns and bias their trajectories in response to different biochemical stimuli, such as soluble ligands (chemotaxis) or cues fastened either to cell surfaces or to the substrate (haptotaxis) [2,43–45]. Haptotaxis seems cell-type specific, dependent on cell-induced tractions, and therefore limited by substrate adhesiveness. Cells’ ability to respond to biochemical stimuli (chemoattraction) is crucial in multicellular organisms. For instance, it allows the sperm to locate the egg during fertilization [46,47]. Neural crest cells are guided toward their appropriate destination during embryogenesis [48–51]. Chemoattraction also enables immune cells to locate foreign invaders [52–54]. Hence, by allowing cells to read the biochemical profile of their surroundings and adapt their behavior accordingly, chemoattraction is essential for the proper functioning of multicellular organisms.

### Biochemical cues

Cells can sense differences in concentrations of organic and inorganic substances. As a result, cells move toward and away from the gradients of these ligands, from bacterial peptides and ECM degradation products to chemokines and growth factors. Some of these proteins can exist in the fluid phase or immobilized (surface bound). Many different cell types can secrete chemokines into the surrounding environment. As a result, they can induce the migration of endothelial cells and promote angiogenesis. Chemokines can also attract angiogenesis-promoting immune cells. Interestingly, cells can even create their own attractant gradients [42], which allow them to migrate collectively [55], and navigate complex routes using self-generated chemotaxis [56]. Thus, cells produce and respond to biochemical cues diffused into the matrix or surface-bound, guiding other cells and their future selves.

Secreted proteins can induce distinct cellular responses (e.g., their migratory phenotype) in different ways. For example, different growth factors, including vascular endothelial (VEGF) and epidermal growth factor (EGF), as well as cytokines such as transforming growth factor beta (TGFβ), stimulate epithelial to mesenchymal transition (EMT). Such transition enables individual cancer cells to detach from an epithelial cluster and move freely, promoting tumor progression. Notably, TGFβ not only drives fibrosis, invasion, and metastasis [57,58], but also induces highly motile amoeboid phenotypes [28]. Furthermore, Lopez-Luque and colleagues [59] demonstrated that some tumoral cells respond to TGFβ inducing and epithelial to amoeboid transition (EAT), after silencing epidermal growth factor receptors (EGFRs). Interestingly, metabolic challenges such as hypoxia can also induce collective to amoeboid transition (CAT) in cancer cells [60]. Independent works have pointed toward TGFβ promoting EMT. Still, some of these studies showed an atypical response to TGFβ, which stimulated different cell types to an incomplete EMT phenotype [61,62]. Cells exhibiting such hybrid EMT phenotype, which promote metastasis, acquire mesenchymal features while maintaining cell–cell adhesions and therefore acting as collectives [63,64]. These findings may suggest that, in the metastatic progress, the role of TGFβ strongly depends on context, including cell and cancer type. Ligand concentration may also influence other cell behaviors. For instance, low concentrations of platelet-derived growth factor (PDGF) can promote cell migration, whereas high concentrations may induce proliferation [65]. Hence, cells acting individually or as a collective can determine not only their own fate but also the fate of others.

### Internalization of biochemical stimuli

**Figure 2.**
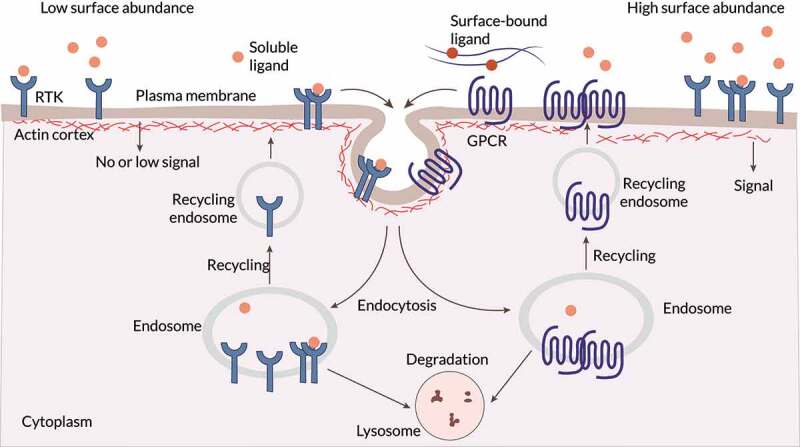
**Sensing biochemical cues** The surface abundance and distribution of transmembrane receptors, such as receptor tyrosine kinase (RTK) and G protein-coupled receptor (GPCR), is a key regulatory step. Locally high surface levels of an individual surface receptor may promote homodimerization and/or clustering, and high surface abundance of two or more of these receptors may also increase heterodimer pairing. Distinct domains within the plasma membrane, as well as the closely apposed and dynamic cortical actin cytoskeleton, affect this key step in receptor activation. The surface abundance of transmembrane receptors is predominantly controlled by receptor endocytosis, which ultimately leads to receptor degradation or recycling. When localized in specific plasma membrane domains, stimulated (ligand bound) or unstimulated (unbound) surface receptors are endocytosed or sequestered. Adapted from Ref. [66].

Biochemical cues bind to transmembrane receptors, triggering cascades of signaling pathways. As a result, the signals initiated by these receptors are transmitted across the plasma membrane and inside the cytosol. There are several classes of these receptors (ion channel-linked receptors, enzyme-linked receptors, and G protein-coupled receptors), which bind to and sense different types of chemoattractants. Receptor tyrosine kinase (RTKs) are the enzyme-linked receptors with the largest population and the widest application, and detect many different growth factors (e.g., EGF, PDGF, and VEGF). In contrast, G protein-coupled receptor (GPCR) is the largest receptor superfamily in eukaryotic cells and recognizes many different ligands (e.g., chemokines, hormones, neurotransmitters, and photons). The spatial distribution of transmembrane receptors over the cell surface was initially considered homogeneous. Subsequent works discovered that the plasma membrane is divided into nanometre-scale domains that can be extended over macrodomains and exhibit different membrane receptor profiles. Some domains may have different amounts of the distinct cell surface receptors, including EGFRs and vascular endothelial growth factor receptors (VEGFRs) [66]. Also, those transmembrane receptors might be present in different configurations (monomeric, dimeric, higher-order oligomers, or clusters) even in the absence of ligands [67–69]. A high surface abundance of a particular transmembrane receptor may promote homodimerization and clustering. Conversely, a high surface abundance of distinct transmembrane receptors would promote heterodimer pairing. Other factors, such as the cytoskeleton organization and ligand stimuli, may bias such membrane receptor profile too (Figure 2) [65,66,70]. At the tissue scale, cells can establish larger macrodomains of the plasma membrane. In such scenarios, cell–cell contacts regulate membrane asymmetry, allowing cells to sense and respond to each other. Transmembrane receptors, which enable cells to probe and internalize external stimuli, are continually being synthesized, internalized, recycled, and degraded.

Cells degrade and recycle surface receptors through membrane trafficking using membrane-bound transport vesicles (Figure 2) [65,71]. Different factors such as ligand concentration, distinct types of stresses, and hypoxia seem to influence the preferred internalization route of RTKs, that is, their sorting toward degradation or recycling. Different GPCR-interacting proteins and arrestins can also influence the GPCR internalization route [72]. Various studies showed that distinct RTK classes remain active during their internalization [65,66,69]. Indeed, in some cases, RTK and GPCR internalization is required for a complete signaling response [73,74]. Whatsmore, transmembrane receptors can activate different effectors depending on whether they are at the plasma membrane or in endosomes. Changes in the spatial distribution generate variations in the internalized signals [65]. For instance, these signals can be localized and amplified over a specific area of the cell surface. Besides, an altered expression of transmembrane receptors can change their spatial distribution, which may impact cell tracking, polarity, adhesion, and cytoskeletal organization during pathological processes (e.g., cancer development and progression) [66,75,76]. Thus, the internalization of transmembrane receptors allows for their dynamic organization over the plasma membrane and may be required for an appropriate signaling response.

Although some receptor classes access many of the same signaling pathways, their dynamics are significantly different. Each cell surface receptor may be activated by distinct ligands, triggering different signaling outcomes [77]. Some ligands can activate different RTKs too [68]. Interestingly, the activity of transmembrane receptors is even possible in the absence of ligands (basal activity). Ligand-bound GPCRs can also trigger the activation of unbound EGFRs through transactivation [78]. In addition, some ligands can bind different receptors together, mediating distinct biological responses. Besides, RTKs directly interact with the plasma membrane and the cytoskeleton. Altogether, surface receptors translate the biochemical profile of the ECM into biochemical signals inside the cell through many different interactions, occurring under a wide variety of circumstances.

By initiating these downstream signaling, chemoattractants influence cells internal organization and their transcriptional regulation. As a result, these ligands may initiate changes in cell polarity. Thus, chemoattractants may bias influence cells’ trajectories, enabling directed migration and different physiological processes, including immune response, wound repair, and tissue homeostasis.

## Probe of biophysical stimuli

Recently, much interest has focused on how biophysical factors, such as the stiffness and the microarchitecture of the ECM, influence cell migration. Still, our understanding of the role of these factors in cell motility is far from complete. Partially, at least, because many of these biophysical cues cannot be incorporated into and studied on flat surface assays. Indeed, 2D studies about the impact of biophysical stimuli in cell migration are limited to planar substrates with stiffness gradients [79], micropatterned barriers (e.g., slabs, micropillars, or microstencils) [80], and other nanometer- to micrometer-scale topographies (e.g., nanoscale ridges, needles, cones, sawtooth structures, or grooves) [81,82]. In 3D environments, cells use different modes of migration (e.g., mesenchymal, amoeboid, lobopodial, collective) based on the local ECM (Figure 1) [20,30,80]. For instance, macrophages use an amoeboid-like migration in porous substrates, whereas in dense matrices such as Matrigel they use a mesenchymal-like one [52]. Furthermore, *in vitro* studies suggest that the speed of migrating macrophages is stiffness dependent. Substrate stiffness can also guide cell migration (durotaxis) [9,83,84]. Indeed, mesoderm stiffening is required and sufficient to trigger the collective migration of neural crest cells during morphogenesis [85]. However, cells may also migrate toward softer environments to generate higher traction forces [86]. The biophysical properties of the tumor microenvironment contribute to cancer development and progression too [87–90]. For example, increasing substrate stiffness led to a switch from proteolytically independent invasion to a proteolytically dependent phenotype in breast cancer cells [91]. Substrate stiffening also promotes EMT by controlling the subcellular localization of downstream effectors [58]. Interestingly, ECM-induced EMT correlates with TGFβ activation by resident epithelial cells. Also, the inhomogeneity of 3D environments may promote clustered cells to switch to a single cell-dominated invasion [92]. Conversely, denser substrates and decreased porosity would lead to the opposite switch, from individual to collective cell migration. Thus, cells can sense the biophysical cues from the microenvironment and adapt their behavior accordingly.

### Biophysical cues

**Figure 3.**
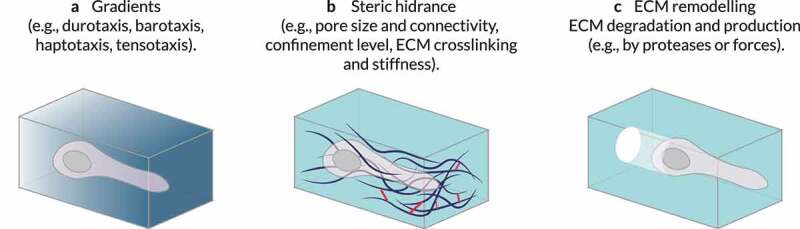
**Extrinsic regulators of 3D cell migration**. Different properties of the surrounding microenvironment can regulate or modulate cell migration. (a) The concentration of each extracellular matrix (ECM) component can vary locally creating, for example, gradients of stiffness (durotaxis) or ligand concentration (haptotaxis), biasing cell motility. (b) The presence and size of pores within the 3D environment – which can be altered by ECM crosslinking and may be dependent on ECM or tissue stiffness – and the level of confinement of cells mediated by the ECM modulate spatial obstruction of the substrate (steric hindrance) to cell migration. (c) Local remodeling (e.g., by proteases or local force causing physical displacement of ECM components) can also influence cell motion. The features of the local microenvironment can be overlapping; for example, increasing the concentration of ECM components can increase local stiffness and alter the sizes of pores. Adapted from Ref. [4].

Many biophysical cues from the surrounding microenvironment can influence cell migration. A list of the primary ECM features regulating or modulating cell migration may include at least the following: (i) ECM topology, (ii) the molecular composition of the ECM, and (iii) the local concentration of each ECM component (Figure 3) [4]. However, many other factors influence cell motility too, such as (i) ECM crosslinking, (ii) gradients of stiffness or ligand concentration, (iii) porosity and pore size within the ECM, (iv) ECM stiffness, (v) ECM (visco-)elastic behavior, and (vi) ECM confinement of cells. Whatsmore, some of these properties may be overlapping [93]. For example, collagen alignment can alter the ECM pore size and the micro-scale stiffness. Fibril diameter and intrafibrillar crosslinking control fibril bending stiffness independently, which correlates with matrix mechanical properties [94]. Increasing the concentration of Matrigel or ECM components (e.g., collagen) can also increase ECM stiffness and alter the size of its pores [4,95]. Therefore, we must study how distinct architectural features (e.g., geometry, porosity, topology) affect cell behavior in these matrices. Lastly, during tumor progression, the organization and composition of the ECM are altered [6]. As a result, tumoral tissue exhibits biophysical properties strikingly different than those of its healthy equivalent. In summary, a wide variety of biophysical features associated with the ECM affect cell motility.

The response of cells to ECM stiffness is cell-type specific [86,96–98]. Still, there is ample evidence that substrate stiffness plays a role in cancer metastasis as tumoral tissue is stiffer than its normal counterpart [3 84,89,99]. Increased stiffness may hinder cell migration due to an excessive steric hindrance [100,101]. Besides, substrate rigidity in 3D may also impact cell–matrix interactions and intracellular activity [18]. Preliminary reports from Higgins and colleagues [102] suggest that decreased cell stiffness drives tumor-cell detachment and migration. On the other hand, in stiffened matrices, cells must either soften or remodel the surrounding environment to avoid migration arrest. Recent studies suggested that ECM rigidity and deformation mediate cell mechanosensing [103].

Fibers comprising the ECM are usually aligned in a specific direction, anisotropically. Moreover, in mammary tumors, aligned collagen fibers are oriented perpendicular to the tumor boundary [104]. Enhanced fiber alignment promotes a more directed cell polarization and migration [105]. Indeed, elongated cells respond more strongly to fiber alignment than those with a rounded morphology. Of note, cell–matrix adhesions and Rho-mediated actomyosin contractility modulate cell responses through the mesenchymal to amoeboid transition (MAT). Besides, the degree of fiber alignment regulates the transition rates between elongated and rounded morphologies. Notably, cells respond to ECM fiber alignment differently based on dimensionality. Fiber alignment modulates protrusion rate and orientation [106]. It also promotes the directed migration of cells [107]. For instance, recent *in vitro* studies suggest that, by aligning collagen fibers, cancer-associated fibroblasts may help tumor cells migrate toward blood vessels during the initial stage of metastasis.

When ECM pores are about the size of cells or slightly smaller, cells seem to migrate more effectively [28,104]. However, if pores are significantly smaller than cells, their nuclei may impede cell migration because of their size, rigidity, and limited deformability [108]. On the other hand, pores bigger than the cell size may also impede migration as cells cannot develop protrusions and adhere to the ECM properly [109]. ECM architectures with narrow pores and short fibers seem to confine cells to a rounded shape and altered protrusion dynamics independently of substrate rigidity or bulk collagen density [110]. Hence, understanding the intricacies of how cells sense all these features may allow us, for example, to develop novel and effective techniques against metastasic diseases.

### Internalization of biophysical stimuli

Mechanotransduction enables cells to probe for biophysical features. It involves different membrane receptors (e.g., ion channels and growth factor receptors), and a wide range of proteins and assemblies, such as integrins and integrin adhesion complexes (IACs) [103,111–113]. Ion channels tightly control cellular voltage through the influx or efflux of ions, which trigger downstream signaling cascades [114–117]. They are activated by distinct stimuli, including ligands, temperature, and force (e.g., tensional stretch, shear stress, membrane tension).

Integrins are one of the primary transmembrane receptors that play a central role in cell–matrix interactions [117–120]. These receptors also act as biomechanical sensors of the microenvironment. As a result, integrins allow cells to sense haptotactic gradients composed of ECM components too [121]. Each integrin binds to specific ECM components and cell surface molecules with specific spatiotemporal distribution patterns in a given tissue [113,118]. Distinct integrins can have overlapping ligand specificity. In such cases, integrins may synergize, antagonize, or complement their activities [122]. Moreover, every cell type has its specific integrin profile, and they can modulate it to adapt to new substrates [23]. Note that altered integrin expression is associated with several types of cancer and other diseases [118,119,123,124].

**Figure 4.**
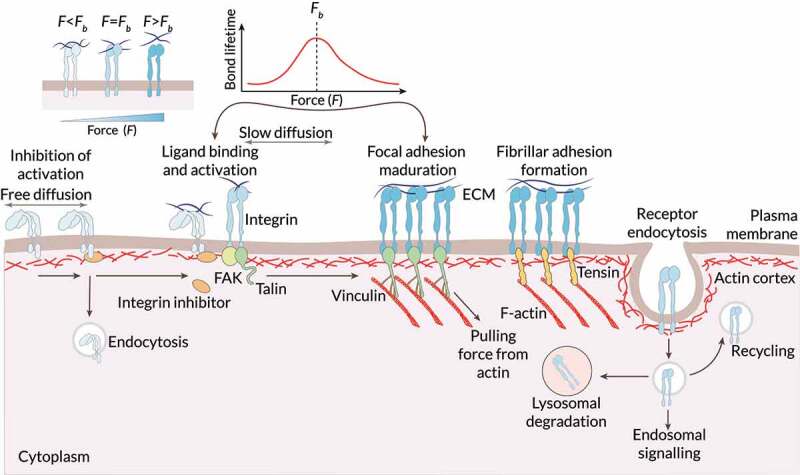
**Sensing biophysical cues by means of the integrin dynamics**. On the plasma membrane, different factors (e.g., the forces from the ECM) enable integrin activation and increased affinity for ligand binding. Inside-out signals regulate displacement of intracellular integrin inhibitors and allow talin to bind to integrins, tightly controlling integrin affinity for ECM ligands. In fibroblasts, recruitment of focal adhesion kinase (FAK) to integrins has been suggested to precede talin recruitment. Integrin activation is also promoted by an outside-in mechanism through ECM binding and force application that slows the diffusion of integrin dimers within the plasma membrane. Force application leads to integrin clustering and the initiation of integrin downstream signaling through the coupling of integrins via talin and vinculin to the actin cytoskeleton. Reciprocally, actin can pull on integrins, further contributing to force generation. In fibroblasts, focal adhesions can mature further to fibrillar adhesions where talin is replaced by tensin. Trafficking of integrins regulates their availability at the plasma membrane. Integrins are constantly endocytosed from the plasma membrane. They are then efficiently recycled, with a small subset of the receptors targeted to lysosomal degradation. Integrins can be endocytosed via multiple different routes depending on the cell type, adhesion status, and cellular signaling pathways that are activated. Force regulates integrin properties. Integrin–ligand binding follows a catch bond behavior. When force (F) applied to the ligand-bound integrin is below the optimal bond force ($F_{B}$), the strength (lifetime) of the bond increases with force. When F exceeds $F_{B}$, the bond lifetime decays with force. Mechanical force (F) acting on integrins through their ligands can favor integrin unbending and subsequent activation, thereby triggering outside-in integrin signaling. Activation increases catch bond behavior, further strengthening the bond. If a given F is applied to an adhesion site, further integrin clustering decreases the force applied to individual integrin dimers. This minimizes elastic energy since it decreases the applied strain, and could thus be promoted. Adapted from Ref. [120].

Integrins are activated through biochemical interactions and by forces transmitted between intracellular and extracellular spaces (Figure 4) [117,119,120]. While activated, integrins have an increased affinity for ligand binding. In turn, extracellular binding and force application promote integrin clustering, triggering signaling pathways that couple integrins to the actin cytoskeleton [99,119,125,126]. These integrin clusters, together with force-induced catch bonds, extend the lifetime of adhesion sites. Their targeted downstream effectors are essential for many processes such as cytoskeletal dynamics and cellular structure. Moreover, some of these processes are fundamental for maintaining cell polarity.

Integrin traffic not only regulates their spatial distribution (i.e., their cell-surface availability) but also IACs turnover [113,127], based, among other factors, on biophysical stimuli [128]. The specifics of integrin trafficking pathways, though, depend on context and cell type [122]. As with other surface receptors (e.g., RTKs, and GPCR), endocytosis allow integrins to be efficiently recycled back to the plasma membrane or degraded by lysosomes (Figure 4). These processes are essential for regulating integrin function and therefore to cell migration and invasion in 3D substrates [127]. Interestingly, crosstalk with RTKs and other co-receptors modulate integrin functions in migrating cells [68,111,112,129]. This crosstalk between integrins and growth factor receptors can enhance growth factor receptor activation and focal adhesion kinase (FAK) phosphorylation [111]. Whatsmore, mechanical stimuli can independently activate growth factor receptors without ligand-induced activation [82,111,122]. The dynamics of these processes allow for adhesion turnover, which is essential for mesenchymal cell migration.

Integrin clustering initiates IACs formation [113,118 126,130]. These IACs allow cells to adhere to their surrounding ECM, probing biophysical cues and transmitting forces. Of note, substrate stiffness and ligand spacing determine an optimal force threshold for IACs formation and coordination with downstream cascades [131]. During this initial stage of IACs formation, several proteins, such as tensin and talin, are recruited to nascent adhesions [104,120]. As a result, downstream effectors, including Ras-related C3 botulinum toxin substrate 1 (Rac1) and the Actin-related protein 2/3 (Arp2/3) complex, are activated, which induces protrusions formation. These nascent adhesions are also critical for ECM haptotaxis [121]. Integrin-mediated force transmission between cells and the ECM mature nascent adhesions to focal adhesions, recruiting other proteins such as paxillin, vinculin, and FAK [125,132]. In turn, FAK activates downstream pathways controlling different cell behaviors such as adhesion and motility [3,133,134]. Recently, nuclear paxillin was also associated with enhanced tumor angiogenesis, growth, and metastasis [135]. Focal adhesions may mature further to fibrillar adhesions in some cell types (e.g., fibroblasts, platelets) [120,122]. These are long, thin, and centrally located adhesions, which enable fibronectin fibrillogenesis. Interestingly, mechanotransduction on stiffer surfaces alters EGFR organization and induces their clustering at focal adhesions [111]. Besides, IACs are not limited to actin-binding cell–ECM adhesions [122]. Instead, distinct proteins, when recruited to integrins, allow for specialized functions and connections with the cytoskeleton. The presence of Matrigel in collagen hydrogels increases the number and size of focal adhesions [95]. Focal adhesions also serve as signaling hubs where several signaling proteins group because of integrin activation and clustering [4]. Recent studies have demonstrated that focal adhesions also form nutrient-sensing hubs, which mediate, among others, spatially restricted growth factor receptor signaling and nutrient uptake [136]. Consequently, these macromolecular assemblies transmit mechanical forces and regulatory signals between cells and the ECM.

## Mechanics of cell migration

Cells rely on the coordination of four core biophysical processes to interact with and migrate through 3D environments: (i) adhesion, (ii) cytoskeletal, and (iii) nuclear dynamics, as well as (iv) matrix remodeling through cell–matrix interactions. The biophysical properties of the ECM modulate several of these biophysical processes. Migration through dense environments requires enhanced cytoskeletal remodeling to displace the surrounding ECM and enable cells to squeeze themselves through narrower pores [60]. Cells also increase their protrusive activity to enhance matrix remodeling and the probe for cell tracks, which would enable a more efficient migration. As a result, cells increase their metabolism while migrating through dense environments to meet higher energy demands [137,138]. Multiple signaling mechanisms tightly regulate these processes [139,140].

The Rho family of small guanosine triphosphatases (GTPases) is involved in many signaling pathways activated during cell migration [141,142]. Rho GTPases such as Rho-related BTB domain-containing protein 1 (RhoBTB1) inhibit invasion [143]. Besides, an altered expression of several Rho GTPases appears in different human tumors and cancers [58,140,144–146]. Rho proteins are also involved in the EMT. As a result, they enable carcinoma cells to metastasize [140]. Hence, Rho GTPases are critical for cell motility.

The opposing actions of guanine nucleotide exchange factors (GEFs) and GTPase-activating proteins (GAPs) regulate the activity of Rho GTPases [58,143,145]. Such dynamic regulation depends on a coordinated and localized activation and inactivation of multiple proteins such as PI3K, FAK, and Src. Indeed, the ability of RhoGEFs and RhoGAPs to form complexes with such proteins is fundamental to spatiotemporal regulation of Rho GTPase activation in migration and invasion [139]. Notice that cellular events can be regulated by integrated signaling networks instead of a specific signaling cascade. Therefore, the same stimuli in different cell contexts could promote distinct responses. The dynamics of such signaling events are thus varied and tightly regulated.

Next, we will summarize our current knowledge of the aforementioned four core biophysical processes enabling cells to interact with and migrate within 3D environments. In particular, we will highlight the roles of (i) cell–matrix and cell–cell adhesions; (ii) the cytoskeletal actin microfilaments, microtubules, and intermediate filaments; (iii) the nucleus; and (iv) cell–matrix interactions enabling matrix remodeling through alignment, degradation, deposition, and crosslinking.

### Adhesion dynamics

Different modes of migration depend on adhesive complexes. For example, individual fibroblasts may use mesenchymal migration mediated by cell–matrix adhesions during wound healing. However, collective migration used by neural crest cells during embryogenesis requires cell–cell junctions [42]. Besides, cell–matrix and cell–cell contacts play an important role in mechanotransduction [103,111].

#### Cell–matrix adhesions for individual migration

**Figure 5.**
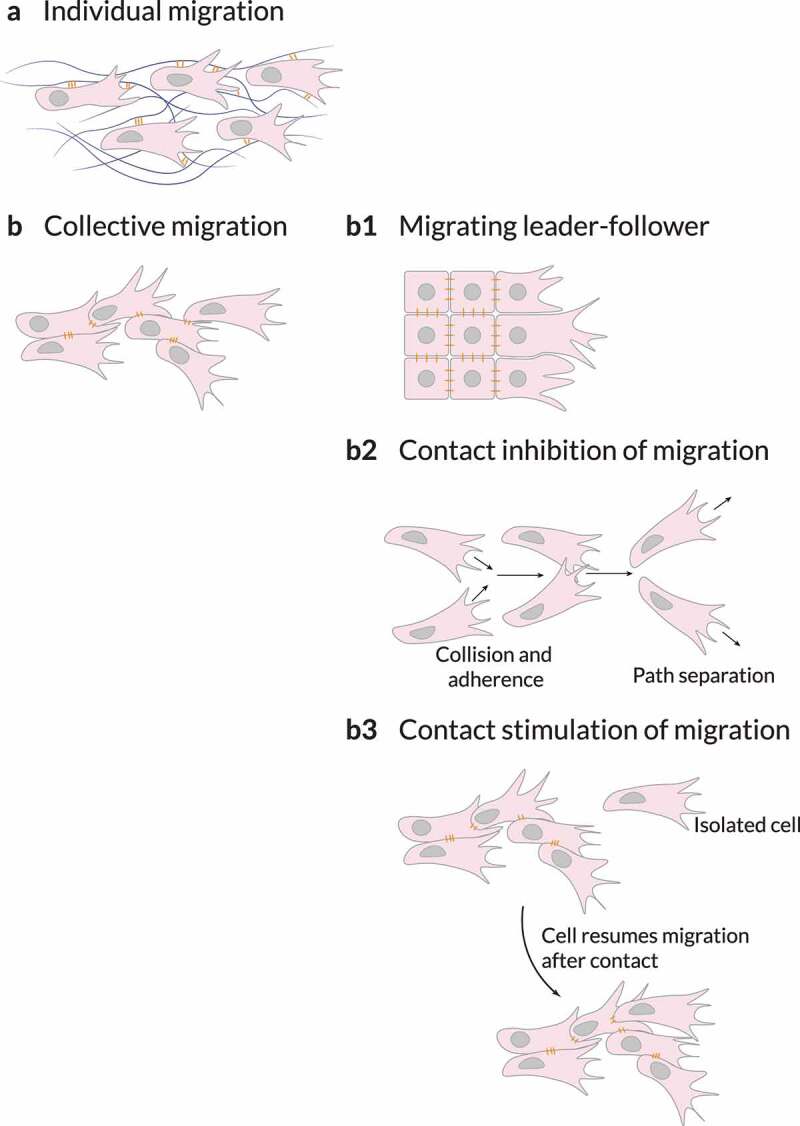
**Cell adhesions**. Non-migratory cells must be stimulated to migrate by transcription factors, growth factors, chemokines or physical forces. (a) They can migrate as loose cohorts of individually migrating cells. (b) Cells can also maintain cohesiveness by adherence using cell–cell adhesion molecules. (b1) When migrating collectively, cells can organize into leaders and followers, in which the leaders – established by signaling cues (for example, by diffusible growth and other factors) or by mechanical cues such as those generated by neighboring cells – provide guidance as long as the biochemical or biophysical signaling is maintained. Cohesive migration of cell populations can be supported by two types of cell–cell interactions: contact inhibition of migration or contact stimulation of migration. (b2) Contact inhibition of migration is a mechanism in which colliding cells migrate in new directions after collision rather than cohering; however, on a population level, this mechanism ensures that cells maintain similar polarities, thereby resulting in directionality of migration in collectives. (b3) Contact stimulation of migration provides a direct mechanism for maintaining cohesion. In this case, cells that migrate away from contact with their neighbors cease migrating and resume migration only after being contacted by another migrating cell. Adapted from Ref. [4].

Cell–matrix adhesions, essential for mesenchymal cell migration, support force transmission between extra- and intra-cellular spaces (Figure 5a). They also allow cells to probe the biophysical properties of the substrate. These adhesions are of particular importance in 3D scenarios where cells have to squeeze themselves across ECM pores. In 3D microenvironments, cell–matrix adhesions are longer and more elongated than the 2D counterpart. Indeed, fibroblasts seem to attach more strongly to the ECM in 3D domains than on flat surfaces. Still, integrin-mediated adhesions are not essential for 3D cell migration. More confining ECM architectures (i.e., smaller pores and shorter fibers) alter protrusion dynamics by reducing, but not eliminating, cell adhesions to the substrate [110]. Moreover, high confined spaces featuring low-adhesion properties abolish focal adhesions. Fast actomyosin retrograde flow allows cells to generate sufficient friction. As a result, cells switch to rapid amoeboid-like cell migration, propelling themselves forward. Active water transport through the cell membrane may induce an osmotic pressure gradient, which can also initiate and sustain friction-driven cell migration in 3D surroundings [104]. However, although cells can migrate without cell–matrix adhesions under specific circumstances, such adhesive complexes are fundamental for many biological responses.

Cells’ ability to adhere to the substrate involves different players. Adaptor proteins, such as talin and vinculin, couple integrins located at IACs to actin microfilaments. As a result, cells’ cytoskeleton binds to the substrate [99,133,147]. Adaptor proteins also interact with cells’ cytoskeleton through intermediate filaments and microtubules [148]. However, the scientific community still lacks a detailed view of how adaptor proteins behave under different conditions. For example, Kluger and colleagues [149] recently unveiled that vinculin acts as a mechanosensitive logical gate, converting the input forces, pulling geometry (e.g., zipper-like vs. shear-like), and magnitude into distinct structural outputs. Mechanical forces generated during actin polymerization or by myosin motors initially exerted to actin microfilaments are transmitted to different adaptor proteins. Then, these forces are transmitted to transmembrane proteins, such as integrins, linking adaptor proteins to the surrounding ECM. According to the molecular clutch hypothesis, contractile forces are only optimally transmitted if the whole system (from actin microfilaments to these adaptor proteins) is engaged. Otherwise, the adhesion complex cannot maintain high force transmission because of an unstructured or fluidized, softened cytoskeleton [150]. Also, preliminary reports from Newman and colleagues [151] showed that IACs in protrusions enable actomyosin-mediated force transmission to the nucleus. The ECM is paramount for this mechanism because substrate rigidity directly controls when contractile forces are optimally transmitted. In fibrilar collagen substrates, effective cell adhesion may require proteolytic activity [110]. Thus, adaptor proteins and other proteins, different factors such as ECM stiffness, and processes (e.g., matrix degradation) play a part in cell–matrix adhesions.

#### Cell–cell adhesions for collective migration

Collective migration depends on cell–cell interactions coordinated with the actin cytoskeleton (Figure 5b) [11,80]. By establishing attachments between cells and coupling their cytoskeletons, cells can sense and transmit forces between them [111]. These attachments also enable stress distribution between cells [10,148]. As a result, cells can integrate external signals from and communicate over longer distances, which allows them to sense shallow biochemical and biophysical gradients [79]. Cell–cell coupling enables multicellular assemblies to migrate and rearrange during morphogenesis and tissue repair [80,152]. These cohesive cell groups ensure the proper formation and repair of organs. Multicellular assemblies may display front-to-back polarity, where leading cells coordinate the migration at the front edge [80,152,153]. For instance, in epithelial monolayers exposed to an empty edge, leader cells drag follower cells by forming large lamellipodia and maintaining robust cell–cell adhesions with them (Figure 5b1). Therefore, cell–cell interactions and collective migration are critical for other fundamental biological processes.

Different cell–cell adhesion systems are fundamental for collective migration, including but not limited to adherens junctions and tight junctions [11,111]. Adherens junctions are central hubs that control cell–cell cohesion and collective cell migration during tissue dynamics and remodeling [11]. Although usually associated with epithelial and endothelial tissues, adherens junctions may also transiently form in mesenchymal cells. Distinct mechanisms (e.g., endocytosis, cytoskeletal regulation) control adherens junctions’ stability. Rho GTPases are also involved in these mechanisms [11,80,141]. Actin cytoskeleton coupling enables contractile forces transmission across adherens junctions [80,111]. On the other hand, tight junctions form a central hub between cell–cell interactions and actin dynamics. The primary role of tight junctions is to function as paracellular gates restricting diffusion based on size and charge. Tight and adherens junctions seal the paracellular space and adhere epithelial cells to one another [154]. They also bind with the actomyosin cytoskeleton. Actomyosin dynamics are essential for the formation, structure, and function of junctions during epithelium homeostasis and morphogenesis. Altogether, each cell–cell junctions have a different role, but all are essential for cells to migrate collectively.

Cells may also repolarize and change their trajectory upon contact with one another. An example of this phenomenon is contact inhibition of locomotion. This mechanism of cell repulsion moves cells away from cell–cell contacts (Figure 5b2) [155–158], and can occur between cells of the same or different type.

Contact inhibition of locomotion is a multistep phenomenon, which initiates upon a collision. Colliding cells accelerate toward each other and form cadherin-based cell–cell adhesions. Then, their protrusive structures toward the contact collapse. Finally, cells develop new protrusions away from cell–cell contacts, separate, and move away. Note that cell–matrix adhesions play different roles in contact inhibition of locomotion (e.g., inducing lamellae paralysis upon collision and enabling separation by disassembling themselves near the contact afterward). Besides, cell–cell and cell–matrix adhesions directly crosslink to actin and regulate cytoskeleton dynamics. Cytoskeletal rearrangements are essential in contact inhibition of locomotion. In particular, the importance of actin microfilaments and microtubules has been demonstrated during the different stages of contact inhibition of locomotion. Small GTPases, which regulate cytoskeletal dynamics, play also a fundamental role in contact inhibition of locomotion. Rac1 activity, initially elevated in the leading edge of the cell, is suppressed near the contact upon collision. In contrast, Rho activity is stimulated around that contact region. Lastly, Rac1 activation is triggered in the edge driving cells repolarization and separation.

Contact inhibition of locomotion opposes cell propulsion [9]. When migrating collective, cells at the edge experience less contact inhibition of locomotion and therefore have more propulsion than those at the core of the cluster. In this scenario, edge cells also have stronger alignment interactions. Further, the collision properties of malignant tumoral cells may influence the alignment of cell motion.

A less recognized phenomenon where cells change their migratory phenotype upon contact with one another is contact stimulation of locomotion [4,159]. Complementary to contact inhibition of locomotion, in contact stimulation of locomotion, cell–cell contacts stimulate collective migration (Figure 5b3). As a result, cells that race ahead of the migrating cohort lose contact with the rest and migrate poorly (if at all) when isolated. Only after restimulation by the group of migrating cells, do these isolated cells regain the initial migratory phenotype. Initially observed in neural crest cells by Thomas and Yamada [159], contact stimulation of locomotion has more recently been observed in prostate cancer cells [160] and myoblast-forming myotubes [161].

#### Interactions between different adhesive complexes and with other cellular components

Distinct cell–cell adhesions, such as adherens junctions and tight junctions, seem to communicate with each other [111]. The regulation of cell–cell junction stability allows for different collective migration modes and patterns [9,80,111]. Furthermore, EMT depends on the regulation of cell–cell adhesions. The stability and strength of these adhesions modulate the degree of the transition. Cell junctions provide positional cues that guide the distribution of RTKs and their ligands [66]. They also transmit physical information, regulating RTKs more directly. Whatsmore, cell–cell contacts can inhibit RTK signaling. The interplay between cell–cell and cell–matrix interactions enables cell monolayers to self-organize, migrate, and evolve [96,162]. This interplay regulates different phenomena such as tissue morphogenesis, EMT, wound healing, and tumor progression. Cell–cell and cell–matrix adhesion are not only interconnected [10]. Instead, the crosstalk between them affects downstream adhesion dynamics and signaling transduction [111]. For example, cadherins and integrins activate different Rho GTPases such as Rac, Ras homolog family member A (RhoA), and cell division control protein 42 homolog (Cdc42). At the same time, Rho GTPases intervene in regulating the formation of integrin-based focal adhesions and cadherin-based adherens junctions. Other studies have revealed pathways controlled by growth factor receptors and cadherins that regulate cell–cell adhesion and cell migration [163]. The coupling to common cytoskeletal and scaffolding structures is fundamental for the cadherin-integrin crosstalk. Therefore, tightly regulated adhesion dynamics are required to enable cell migration plasticity.

### Cytoskeletal dynamics

**Figure 6.**
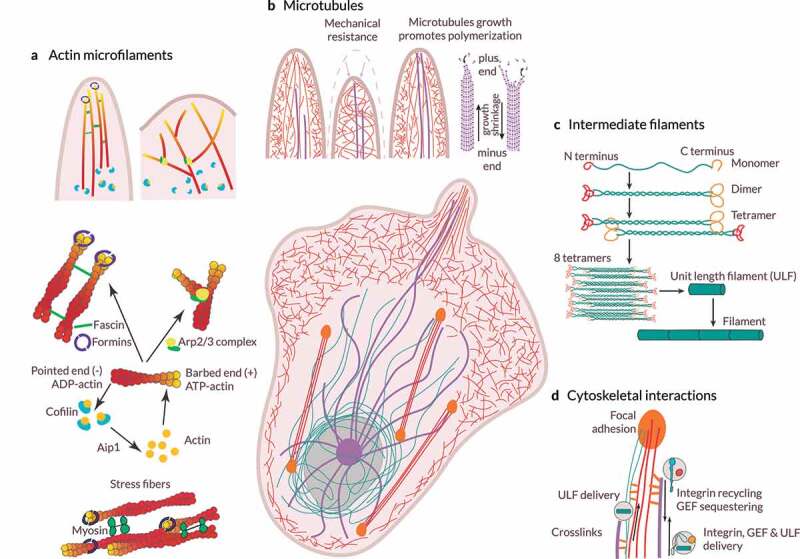
**Cytoskeletal dynamics.** (a) *Assembly and organization of the actin microfilament network*. The Arp2/3 complex nucleate branched actin microfilaments. Conversely, unbranched filaments may be nucleated *de novo* by the formins or generated from a preexisting arp2/3-nucleated network. Actin filaments grow toward the plasma membrane, generating forces that move forward the leading edge. In filopodia, fascin is the main actin microfilament cross-linking/bundling protein. Cofilin triggers actin microfilament disassembly. (b) *Microtubule structure and functions*. Microtubules are anchored at the centrosome and grow toward the cell cortex. Microtubule stiffness paired with the viscosity of the cytoplasm allows them to resist large compressive forces. Microtubule assembly and disassembly result in pushing and pulling forces. Stiff microtubules may provide mechanical support against membrane retraction when actin polymerization is driving membrane protrusion. Also, the growth of microtubules leads to actin polymerization in protrusions. The binding of actin microfilaments and microtubules through crosslinks allows actin microfilaments to guide microtubule growth toward focal adhesions. (c) *Organization and assembly of intermediate filaments*. Monomers associate to form dimers, dimers then associates to form a staggered tetramer, eight tetramers associate to form a unit-length filament (ULF), ULFs anneal to form a thick filament, and further annealing of ULFs results in filament elongation, which is followed by compaction to achieve the final intermediate filament. By organizing into a cytoplasmic nuclear cage, intermediate filaments protect the nucleus against compressive forces. Intermediate filaments also provide mechanical support for the plasma membrane in contact sites with other cells and the ECM. (d) *Cytoskeletal interactions*. Both actin microfilaments and microtubules can act as transport tracks of ULFs and bind to intermediate filaments through crosslinks. Further, microtubules act as transport tracks, enabling the delivery and recycling or sequestering of integrins and other signaling molecules, such as guanine nucleotide exchange factors (GEFs). As a result, microtubules regulate different processes, such as mechanotransduction and actomyosin contractility.

To navigate through complex and constraining environments and overcome physical barriers, cells may remodel their cytoskeleton [2]. The cytoskeleton (Figure 6) is a dynamic network of fibrillar structures located in the cytoplasm of cells [164–166]. This fibrillar network allows cells to modulate their shape and migrate by creating a viscoelastic environment within themselves [167,168]. In eukaryotes, the cytoskeleton comprises actin microfilaments, microtubules, and intermediate filaments. These three cytoskeletal components have starkly different stiffnesses and mechanical behaviors. Besides, they could often spread over the entire cell because of their length and straight shape [168]. Next, we will take a closer look at each of these cytoskeletal components and how they are involved in cell migration.

#### Actin microfilaments

##### Actin (de)polymerization

Cell migration depends, among others, on the dynamic formation and disassembly of actin microfilament networks (e.g., filopodia, lamellipodia, invadopodia), which differ in their structure and functionality (Figure 6a) [139,169]. These different actin-based structures are also located in specific subregions of the plasma membrane. Distinct external cues and downstream effectors are involved in actin dynamics. For one, the (dis)assembly of actin microfilaments and monomer recycling in lamellipodia are regulated by actin-binding proteins. Capping protein, cofilin, profilin, and cyclase-associated protein are some examples of actin-binding proteins. Kinase-phosphatase networks, small GTPases, and membrane phospholipids such as phosphatidylinositol 4,5-bisphosphate (PI45P_2_) tightly regulate the activities of these actin-binding proteins [170]. Receptors located at the plasma membrane, including RTKs, can initiate signaling pathways where Rho GTPases may take part. Indeed, the Rac, Cdc42, and Rho subfamilies promote actin cytoskeleton reorganization: from the formation of actin-based structures and cell polarization to stress fiber formation and Rho-mediated contractility [171 172]. For instance, different stimuli, including growth factors (e.g., PDGF, EGF) and integrin-mediated cell–matrix adhesions, activate Rac. In turn, Rac activation stimulates PI3K and the Arp2/3 complex [173]. Rac activation also creates a positive feedback loop that promotes active Rac accumulation at the cell front. Note that PI3K is paramount for distinct mechanotransduction pathways of, among others, the cardiovascular system [174]. Also, PI3K inhibition reverse fish keratocytes directed migration in electric fields (galvanotaxis) [175]. However, during collective migration, PI3K inhibition does not reverse the directed migration of large groups of these cells. Notably, smaller groups do not exhibit persistent directional migration.

The Arp2/3 complex initiates the growth of new actin microfilaments, branches of older actin microfilaments (Figure 6a). Interestingly, the Arp2/3 complex acts as an actin amplifier as it stimulates the production of its own drivers (positive feedback loop) [176]. Conversely, formins and enabled/vasodilator-stimulated phosphoprotein (Ena/VASP) proteins promote nucleation and elongation of unbranched actin microfilaments at the barbed end of actin microfilaments (Figure 6a) [140,173,177,178]. Indeed, the formin Diaphanous-related formin-1 (mDia1) localizes at the leading edge of some cells (e.g., T-cells) and cooperates with the Arp2/3 complex to initiate lamellipodium formation. The activation of Cdc42 stimulates PI3K, the Arp2/3 complex, and Rho-associated protein kinase (ROCK)-mediated myosin contractility [177]. Heavily branched actin microfilaments made up the cytoskeleton of lamellipodia (Figure 6a). Conversely, filopodia consist of tightly packed, parallelly aligned actin microfilaments, with fascin as their main cross-linking/bundling protein (Figure 6a) [140 172]. Indeed, Rac1 and Cdc42 stimulate lamellipodia and filopodia formation, respectively [171,172,177]. As actin microfilaments grow, they push and protrude the plasma membrane forward [148]. By pushing the plasma membrane, actin microfilaments increase membrane tension, which may act as a long-range inhibitor for protrusions anywhere else under specific conditions [179]. Recent reports on flat surfaces showed that protrusion initiation requires local depletion of actin-plasma membrane links acting in coordination with actin polymerization [180]. The density of membrane-proximal actin microfilaments is low at the leading front and high at the rear [181]. Cells migrating in one, two, or three dimensions exhibit stable gradients of membrane-proximal actin microfilaments. By locally decreasing the density of membrane-proximal actin microfilaments through cofilin, cells may enable Rac-mediated protrusions onset, directing and promoting cell migration.

In contrast, ADF/cofilin, a family of actin-binding proteins, is associated with the rapid depolymerization of actin microfilaments (Figure 6a). Of note, ADF and Cofilin1 are also required to prevent over-accumulation of stress fibers and associated focal adhesions. They promote cortical actin flow as well as the leader bleb-based migration of constricted cells [182]. Also, they modulate nuclear shape, movement, and integrity [183].

Proteins involved in signaling pathways activated by extracellular cues, such as PI3K, Rac1, and FAK, influence actin dynamics in different ways, regulating protrusion formation, stabilization, length, and lifetime [184–186]. Interestingly, in 3D substrates, protrusive activity increases with collagen density [137]. Cells’ dependency on ECM remodeling to migrate in dense environments could explain such behavior. Furthermore, substrate stress relaxation regulates filopodial protrusions (i.e., their lifetime, length, and number) and cell migration [187]. Overall, actin microfilaments dynamics, which are tightly regulated (in time and space), are fundamental for cell polarity and motility.

##### Contractile forces through the actin-myosin complex

Rho/ROCK signaling, including the RhoA effector, promotes focal adhesion formation and actomyosin-mediated contractility upon integrin-ECM engagement [140,173,177,188]. Rho/ROCK suppression triggers the amoeboid to mesenchymal transition (AMT). The serine/threonine kinase ROCK cooperates with mDia to assemble actomyosin bundles (e.g., stress fibers). Besides, Rac and ROCK negatively regulate each other [140]. Actomyosin contractility, together with Arp2/3-mediated actin polymerization, generates a retrograde flow of actin microfilaments [189]. When engaged by focal adhesions, this retrograde flow of actin microfilaments promotes traction force. Focal adhesions transmit pulling forces generated by these bundles to the ECM. Moreover, as traction forces increase, so does the size of focal adhesions [190]. As a result, cells can propel themselves forward, not only reorienting and lengthening the surrounding substrate fibers but also increasing their density [191–193]. Of note, according to the molecular clutch hypothesis, such forces may not be optimally transmitted depending on substrate features (e.g., stiffness, viscoelasticity, and stored strain energy) [103,187,194]. An enhanced actomyosin activity and cell contractility enable cells to migrate against stiffness gradients [195]. Therefore, metastatic cells (e.g., mammary, lung, prostate) may exhibit an adurotactic behavior in their tumor-specific niche. However, less contractile cells tend to durotax on flat surfaces.

In collective migration, contractile actin cables may appear across neighboring cells [80]. The associated actomyosin structures are coupled through adherens junctions or tight junctions to propagate tension, for instance, during tissue repair. Notably, cells seem to migrate along stress orientations, minimizing shear stresses. The alignment of actin microfilaments influences how much tension can be generated by these myosin motors [188]. Besides, cortical tension presents a biphasic response on the level of connectivity. In networks too loosely connected, stresses do not propagate, but those densely connected are too rigid and, although stresses do propagate, such networks cannot actively be remodeled. As a result, cells may actively regulate the connectivity of their actin cortex while changing their shape. In summary, the Rho/ROCK signaling is essential for cells to exert actomyosin-generated contractile forces over the ECM.

Stress fibers are essential for adhesive-dependent migration, as they couple focal adhesions to the cytoskeleton and the nucleus [196–198]. Different stress fiber subtypes (based on their location, composition, and anchorage to focal adhesions) bear unique mechanical properties and structural roles [199]. Vignaud and colleagues [200] demonstrated that stress fibers are not independent structures with discrete connections between them. Instead, stress fibers are embedded entirely in a contractile cortical actin network. This cortical meshwork allows for contractile forces exerted by stress fibers to propagate across the entire cell, actively contributing to traction force transmission to focal adhesions. Consequently, the contraction of the cortical meshwork impacts the overall magnitude of cells’ contractile energy. Interestingly, Tavares and colleagues [201] demonstrated that a transient accumulation of stress fibers increases cell rigidity before cells acquire malignant features. Later on, a higher Src contractility would disassemble stress fibers to facilitate cell migration.

Although initially stress fibers were thought to be an artifact of 2D culture, more recent publications indicated that contractile stress fibers are also fundamental *in vivo* [197]. For instance, transmembrane actin-dependent nuclear lines, stress fibers crossing the nuclear envelope and essential for nuclear movement, are also present in cells within 3D cultures [202]. Distinct cell types exhibit differences in stress fiber organization in 3D [203]. For example, pancreatic fibroblasts cultured in soft matrices displayed randomly organized stress fibers, while in those within stiffer ECMs, stress fibers presented a more organized pattern. Conversely, cancer-associated fibroblasts exhibited well-organized stress fibers. Still, mammary epithelial cells (MEC) within mechanically tunable 3D culture models did not present stress fibers [204], which may suggest that stress fibers formation is context-dependent. Indeed, amoeboid-like migration seem to lack stress fibers [167] and does not require Rac/Cdc42-driven actin polymerization [140]. Thus, mesenchymal migration requires stress fibers to transmit pulling forces across cells’ cortical actin meshwork.

#### Microtubules

Microtubules are also involved in several processes associated with cell migration. For one, their ability to resist high compressive loads and generate pushing forces makes them a relevant contributor to protrusion formation and maintenance [205,206] (Figure 6b). They can also generate pulling forces to move the cell nucleus and facilitate rapid and directional transport of specific cellular components based on cell polarity. Microtubules growth would activate Rac-mediated actin polymerization, whereas depolymerizing microtubules would increase actomyosin contractility via Rho activation [206–209]. Note that microtubule outgrowth promotes a reduction in focal adhesion size and disassembly [190,208,210]. Moreover, RhoA and formins such as Diaphanous-related formin-2 (mDia2) regulate microtubule stabilization. Bouchet and colleagues [211] showed that the elongated shape of mesenchymal cells and their migration in 3D environments (*in vitro* and *in vivo*) requires persistent microtubule growth at the cell cortex. Interestingly, substrate stiffness regulates the polarization of the microtubule network during cell migration [212]. Further, ECM stiffening stabilizes microtubules and reorganizes the microtubule network [207]. Therefore, the ability of microtubules to generate pushing and pulling forces supports protrusive structures and cell organization, and its dynamics – regulated by Rho signaling and the ECM – influence cell morphology and migration.

Regarding molecular trafficking to and from the plasma membrane, microtubule motors serve as cargo tracks for cytoskeletal regulators and components, from integrins, Cdc42, and Rac GTPases to intermediate filaments (Figure 6d) [205,206,208]. They also carry messenger ribonucleic acid (mRNA) encoding proteins involved in actin polymerization, such as the Arp2/3 complex. Microtubules participate in matrix metalloproteinase (MMP) exocytosis [205 208,209]. Different studies suggest that microtubules may further act as an endocytosis controller [205]. Microtubules anchored to the plasma membrane serve as tracks for the transport of exocytic vesicles to focal adhesion sites. Consequently, they allow for focal adhesion disassembly and promote their turnover. Hence, microtubule-based intracellular trafficking contributes to cell polarization, protrusion formation, and focal adhesion turnover during migration.

By interacting with other cytoskeletal networks and cross-linking proteins, microtubules are guided toward focal adhesion and establish stable anchorages in their vicinity (Figure 6d) [208,213]. Formins mDia1 and mDia2 take part in the orientation and alignment of the microtubule and actin networks in different cell types. Intermediate filaments may also play a role in this process, but further studies are required to shed some light on this matter. The microtubule-anchoring machinery is crucial in regulating focal adhesion dynamics and cell migration in response to specific ECM components. Besides, this mechanism might be cell type-dependent and cue-specific. Microtubules can also affect Rho GTPase signaling and stress fiber assembly [133,205,208]. Recent studies on astrocytes depicted a novel crosstalk between actin and microtubules [214]. In particular, rigidity-dependent microtubule acetylation would alter the dynamics and distribution of focal adhesions, as well as actomyosin contractility. These interactions, downstream of integrin-mediated signaling, would promote mechanosensitive migration. Thus, actin microfilaments are crucial for cell migration because of their role in protrusions formation and stabilization, focal adhesion turnover and regulation, cell polarity, and membrane vesicle trafficking [133,167,208].

#### Intermediate filaments

Intermediate filaments play a leading role in reinforcing cell structure and organizing cells into tissues. They maintain the mechanical integrity of the cytoplasm and regulate the organization of cellular organelles. Although the intermediate filament structure is highly flexible, intermediate filaments are more stable than actin microfilaments and microtubules, which allows for their role as scaffolds.

Intermediate filaments can spread through the entire cell cytoplasm, encapsulating the nucleus (Figure 6c) [133 215,216]. The spatiotemporal localization of intermediate filaments is phosphorylation-dependent. Moreover, these phosphorylation events have a functional role in different cellular processes, including cell migration [216]. For instance, intermediate filaments promote the formation and maturation of focal adhesions, which stabilize FAK, and influence integrin clustering, recycling, and motility [216–218]. They also influence signaling pathways regulating actin dynamics, cell polarity, and cell migration.

Regarding intermediate filaments’ structural role, they provide mechanical support for the plasma membrane in contact sites with other cells and the ECM (Figure 6) [215,216]. They can also behave as an elastic and conductive network to transmit force and propagate mechanical stimuli within and between cells via adhesion complexes. Indeed, tensile forces reinforce stress fibers by a coordinated effort between Rho signaling and the intermediate filament network. Still, at larger forces and extensions, intermediate filaments deform in a plastic manner, stiffening and decreasing their diameter [215]. Besides, once organized into networks, intermediate filaments acquire viscoelastic properties based on the number of crosslinks and which intermediate filament proteins are involved.

Intermediate filaments may participate in protein traffic by interacting with microtubules and with intracellular compartments and regulators of membrane trafficking. They also assemble into the nuclear lamina – which binds to the inner nuclear membrane and the chromatin – and act as a nuclear scaffold and mechanosensor [219–221]. Moreover, the composition of the intermediate filament network is cell-type specific. It depends on the mode of migration and thus on the properties of the surrounding ECM. The intermediate filament network may be optimized to protect the cell and regulate the distribution of actomyosin pulling forces throughout the cell [217]. Additionally, recent studies suggest that intermediate filaments optimize collective cell migration by regulating actomyosin-generated forces [153,222]. Hence, intermediate filaments play different roles in distinct cellular regions and influence several processes involved in cell motility.

#### Interactions between different cytoskeletal components and with other cellular units

Although often viewed as three separate entities, actin microfilaments, intermediate filaments, and microtubules cooperatively interact with each other [167,213,216]. For example, through multiple direct, indirect, and steric interactions, actin microfilaments and microtubules influence intermediate filaments organization (Figure 6d). Moreover, perturbing actin microfilaments, microtubules, or their associated molecular motors can trigger intermediate filaments collapse. Cross-linking proteins hold together actin microfilaments and myosin motors in stress fibers. In turn, stress fibers bind to the microtubule network enabling cytoskeleton contractility [133,223]. Vimentin (one of the most abundant members of the intermediate filament family) stabilize microtubules by direct interactions, decreasing microtubule catastrophe and increasing the rescue of depolymerizing microtubules. Furthermore, actin seems to modulate microtubule dynamics and their lifetime based on the actin network architecture. Shanghvi-Shah and colleagues [216] also noted that cells use the available cytoskeletal network to facilitate adhesion and cohesion and balance intracellular tension and externally-derived stresses. More recently, Doss and colleagues [224] showed that at least in 2D substrates, active and passive cytoskeletal stresses regulate cells’ ability to respond to ECM stiffness. They also found that crosslinks and the relative cell-to-ECM elasticity modulate the organization of the actin cytoskeleton. Tension transmitted through the ligand-receptor axis is crucial for the organization of the actin cytoskeleton, at least in T cells [225]. Integrin-based adhesions mediate interactions between microtubules and the actomyosin network [190]. These interactions strongly influence focal adhesions too. The coupling between microtubules and integrins locally regulates Rho/ROCK signaling. It also modulates the formation of myosin filaments. In turn, these myosin filaments act as controllers of integrin-based adhesions. Microtubules disappear from trailing protrusions before or during their retraction [226]. Notably, microtubule depolymerization locally coordinates actomyosin contractility and competing protrusions when cells migrate within complex environments [227]. Other studies on flat surfaces showed that the architecture of the actin network defines the position of the centrosome, the main organizer of microtubules [228]. In particular, the centrosome is located at the geometric center of an inner space devoid of actin bundles. Nonetheless, the spatial distribution of cell adhesions regulates the anisotropy of the actin network. Therefore, this location may not be the geometric center of the cell. Besides, based on the level of actomyosin contraction, the nucleus may displace the centrosome from this position. Noteworthily, the cortical actomyosin network modulates the organization of components of the plasma membrane, and the plasma membrane composition can also regulate cytoskeletal dynamics [229]. Such dynamic interplay between plasma membrane organization and the actin cytoskeleton provides the cell with a stable yet flexible cell surface that can continuously adapt to the surrounding environment.

Cytoskeletal dynamics, initiated by cell migration, activate transcriptional coactivators Yes-associated protein (YAP) and Tafazzin (TAZ), triggering a transcriptional regulation program. Indeed, FAK controls YAP/TAZ nuclear translocation via the RhoA pathway, which is promoted by increasing ECM stiffness and faster stress relaxation [3,140,230,231]. Interestingly, the nuclear transport of YAP and other transcriptional activators may not depend on contractility per se [16]. Rather, it would rely on contractile strain energy transmission to the nucleus and stress generation in the nuclear envelope. This transcriptional regulation program feeds back to modulate cell mechanics, maintain a responsive cytoskeletal equilibrium, and prevent migration arrest [232]. Cell spreading on flat substrates promotes stress fiber formation and YAP/TAZ nuclear shuttling through Rho GTPases. Once in the nucleus, YAP regulates cell mechanics by controlling focal adhesion assembly [233,234]. Moreover, the activity of YAP/TAZ – which limits cytoskeletal tension and focal adhesion maturation – , although not required for initiating cell migration, is essential for persistence cell motility [235]. Transcriptional co-factors YAP/TAZ are also required in and induce several steps of the invasion-metastasis cascade [236,237]. Notably, YAP not only promotes focal adhesion assembly but also tumor invasiveness by regulating FAK phosphorylation in breast cancer [238]. Besides, YAP/TAZ activity also enhances TGFβ signaling, which drives substrate stiffening [3], and crosstalks with VEGF during angiogenesis [233]. Nevertheless, the role of YAP in mechanotransduction is context-dependent. Indeed, YAP does not mediate mechanotransduction in breast cancer [204] but does so in other *in vivo* contexts such as pancreatic cancer [29,239].

In summary, all three cytoskeletal networks must act in coordination for an efficient cell migration [167]. They not only share common regulators, but each of them can also influence the other two through cytoskeletal crosslinks or signaling pathways. As a result, cells can adapt to an always-changing environment. Such crosstalks between actin microfilaments, intermediate filaments, and microtubules are involved in cell polarity, protrusions formation, cell adhesion, and contractility. Moreover, all three cytoskeletal components are associated with cancer by interacting with signaling pathways or through proteins that participate in their dynamics [177]. Overall, different signaling effectors tightly regulate the dynamics of the cytoskeleton. They can be dependent on cell type and the profile of the surrounding microenvironment. They are also fundamental for cell motility.

### Nuclear dynamics

**Figure 7.**
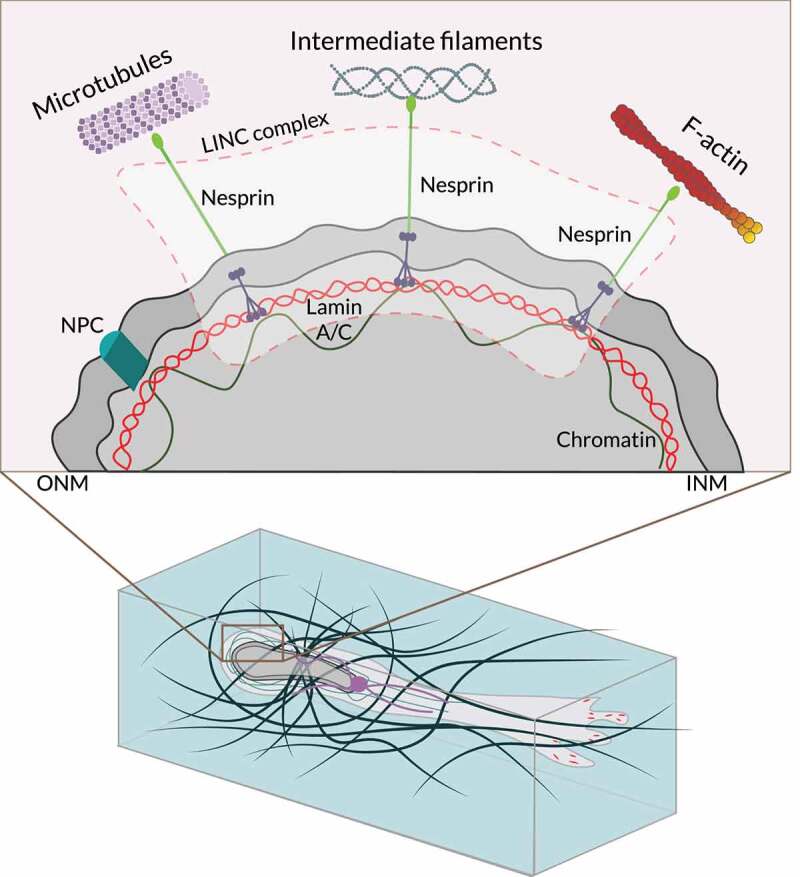
**The nucleus during 3D cell migration**. Mesenchymal cell migration within the extracellular matrix (ECM) requires multiple steps, including nuclear rotation and repositioning. Translocating the bulky nucleus of migrating cells through ECM barriers can become challenging unless the ECM is loose or highly pliable. Alternatively, the nucleus can be used to drive lobopodial cell migration, acting as a pressure-generating piston. Also, during amoeboid migration, cells can use the nucleus as a mechanical gauge or ruler by presenting it anteriorly to ‘measure’ the diameter of pores or passages in the ECM microenvironment. The cell then translocates through a passageway that is sufficiently wide to accommodate the bulky nucleus as the route of least resistance. The LINC complex is at the center of the nuclear-cytoskeletal coupling. On the cytoplasmic side, different nesprin isoforms connect the nucleus to the cytoskeleton. In the perinuclear space, nesprins bind SUN proteins, which span the inner nuclear membrane (INM) and interact with the nuclear lamina through lamin A. Emerin, a protein from the inner nuclear membrane, anchors SUN protein to lamin A and interacts directly with chromatin. NPC, nuclear pore complex. ONM, outer nuclear membrane. Adapted from Ref. [133].

The nucleus is the largest, most complex, and organized organelle within the cell. It is also the most rigid. It comprises different structures such as the nuclear envelope, the lamina network, and chromatin, a complex of DNA and proteins forming the chromosomes of eukaryotic cells (Figure 7). In 1D and 2D environments, establishing cell polarity and migration does not depend on the cell’s nucleus [240]. Still, in 3D domains, it may be essential for proper cell contractility and migration [241]. For example, in confining viscoelastic environments, mesenchymal stem cells (MSCs) create migration paths through a nuclear piston [242]. Amoeboid cells often migrate with their nucleus in front of the microtubule-organizing center (MTOC) as well as the Golgi apparatus (Figure 1) [226,227]. In this configuration, the nucleus would act as a mechanical gauge, enabling cells to distinguish between pores of different sizes. As a result, cells would preferentially migrate along the path of least resistance. Conversely, the posterior passage of the MTOC beyond an obstacle or through a gap would determine the future trajectory of the cell. Then, all but the leading protrusion should retract by cutting off their microtubule supply. Note that, in confined environments, the nucleus is the main source of steric hindrance for 3D migration [108]. Recent studies reported that HT1080 (fibrosarcoma) cells within confined 3D substrates show speed accelerations by nucleus deformation and recoil [241]. Nuclear dynamics can thus also play a fundamental role in 3D cell migration.

#### Interactions between the nucleus and other cellular components

Cells cultured on rigid flat surfaces spread and flatten their nucleus [243]. Conversely, on soft 2D substrates and in 3D hydrogels, cells promote a rounded or elliptical nuclear shape. Confined spaces have low porosity and constraining micropores. Besides, sometimes cells need to cross physical barriers. In such scenarios, cells may deform and change the morphology of their nuclei (Figure 7) [221]. Cells would also attach to the ECM via integrins and focal adhesions, while stress fibers exert high contractile forces transmitted to the nucleus through nuclear anchorage proteins [220,244]. The linker of nucleoskeleton and cytoskeleton (LINC) complex and the nuclear pore complex are some of the main players enabling nucleus-cytoskeleton interactions [240 245]. The LINC complex couples these two cellular components together, whereas the nuclear pore complex allows the transport of molecules across the nuclear envelope. Furthermore, the LINC complex is also essential for nuclear mechanotransduction and translocation [21,220,240,246]. Note that the LINC complex includes two protein domains, which span the inner nuclear membrane and the outer nuclear membrane. Different proteins such as nesprins bind the cytoskeleton to the nucleus through proteins from the inner nuclear membrane Sad1 and UNC-84 domain containing 1 and 2 (SUN1/2) (Figure 7) [133,148]. Indeed, by accumulating at the front of the nucleus during confined cell migration, nesprins contribute to pulling it forward through narrow micropores and constrictions [244]. This nucleus–cytoskeleton coupling allows, for instance, microtubules to interact with proteins from the outer nuclear membrane, exerting mechanical forces onto them. In turn, proteins from the outer nuclear membrane relay these forces to the proteins from the inner nuclear membrane, the nuclear lamina, and chromatin [245]. These mechanical forces may alter the nuclear shape and induce nuclear envelope invaginations. Also, actin microfilaments located above the nucleus (perinuclear actin cap) align cells nuclei with the orientation of migration in some cell types (e.g., fibroblasts) [247]. As a result, cells can adapt and respond to external cues from the ECM.

Exerting high pushing and pulling forces may not be sufficient for cells to overcome these obstacles though. Cells may also need to deform and change the morphology of their nuclei to migrate (Figure 7) [221,247]. Cells can modulate the ratios of lamins located in this organelle [220,248–250]. As a result, cells contribute to the nucleus viscoelasticity by regulating the nuclear morphology and deformability. Mukherjee and colleagues [251] inhibited lamin A/C phosphorylation in HT-1080 fibrosarcoma cells, which increased their nuclei stiffness. Those cells migrate through 3 μm pores less efficiently than within 5 μm pores. They exhibited a dramatic change in nuclear circularity, suggesting that their nuclei underwent plastic deformation. Also, the proportion of nuclei with blebs after migrating through such pores increased threefold compared to the control group. Shiu and colleagues [252] showed that lamin A/C null fibroblasts exhibited a strongly reduced integrin clustering into the perinuclear region. The authors also reported an impaired YAP nuclear translocation.

Interestingly, Harada and colleagues [253] showed that 3D cell migration is biphasic in lamin-A levels. Moreover, partial loss of lamin-A is associated with several types of cancers (e.g., lung, breast, colon, ovarian, and prostate) [243]. While lamina dominates the mechanical resistance at large deformations, chromatin primarily governs such behavior for small ones [249]. Indeed, cells can change the balance of open and condensed chromatin within their nuclei [219,250]. For instance, confined conditions in 3D induce chromatin decompaction and seem to decrease nuclear stiffness. Variations in substrate rigidity can also drive changes to the nucleus and chromatin state [221,254]. Indeed, stiffer ECMs increase lamina-associated chromatin and the number of accessible chromatin sites. Such an event induces a tumorigenic phenotype in mammary epithelium. Interestingly, microtubules may also alter lamin phosphorylation and regulate chromatin dynamics [245]. The former, through the tension exerted onto the nucleus, while the latter by mediating the transport of specific molecular cargo within or to the nucleus. Microtubules not only interact with the nucleus through the LINC complex. They also force the transport of effector molecules and DNA repair proteins through nuclear pore complexes to influence chromatin and promote genome stability, respectively.

Constriction-induced deformation of the nucleus can have deleterious effects such as nuclear envelope rupture and excessive DNA damage (Figure 7) [220,240,255]. Cells have some protective mechanisms against these events. The nuclear lamina is an organized meshwork of different lamins (i.e., intermediate filaments) underlying the nuclear envelope and separating the nucleus from the cytoplasm. Together with the cytoskeleton, it protects the nucleus against high nuclear stress [243]. Interestingly, Nava and colleagues [219] recently showed that persistent, high-amplitude stretch triggers a protective mechanism against DNA damage. As a result, the supracellular alignment of tissue redistributes stress before it reaches the nucleus. Such tissue-scale mechanoadaptation involves a separate signaling cascade mediated by cell–cell contacts. This process allows cells to switch off the nuclear mechanotransduction and restore their initial chromatin state. Defects on nuclear dynamics are associated with the onset of devastating diseases [220].

Novel studies on MSCs showed that the nuclear envelope is wrinkled on soft 2D hydrogels [16]. However, on stiff 2D substrates (plastic or rigid glass), most cultured MSCs exhibited smooth nuclei, that is, little to no nuclear envelope wrinkling. A similar trend emerged in 3D systems, where MMP-degradability would determine the nuclear envelope morphology. Cell spreading would only happen after cytoskeletal tension removed nuclear envelope wrinkling in cells cultured on flat surfaces. Robust focal adhesion maturation would also require a taut nuclear envelope. In MMP-degradable hydrogels, MSCs exhibited prominent stress fibers and nuclear envelope wrinkling caused by actin impingement. Interestingly, a wrinkled nuclear envelope may also be associated with the chromatin-dominated regime of mechanical resistance. Conversely, a nuclear envelope with no wrinkles would indicate that the nucleus is under higher deformations and that lamins are the leading mechanical regulator of nucleus rigidity.

Recent works have proven the nucleus’s ability to measure cellular shape variations [256,257]. In particular, cell confinement below a threshold height deforms the nucleus. It also triggers actomyosin contractility, promoting fast amoeboid cell migration. As a result, cells might avoid getting stuck in their surroundings, of relevance during cancer cell invasion, and immune cells patrolling across peripheral tissues. It may also be paramount for progenitor cell motility within a highly crowded cell mass of a developing embryo. Hence, the dynamics of cells’ nuclei allow them to migrate even across some of the most challenging 3D environments.

### ECM remodeling through cell–matrix interactions

Cells are continuously interacting with the ECM, not only probing for cues but also remodeling its structure [2,10]. Such interactions between cells and their extracellular environment involve distinct mechanisms. The cell phenotype and the profile of the substrate determine which of these mechanisms are activated. For example, hydrogels with higher stress relaxation amplitudes seem to promote cell penetration and ECM remodeling [35]. This would enhance cell elongation, migration, and proliferation [35].

#### Aligning ECM fibers by exerting contractile forces

**Figure 8.**
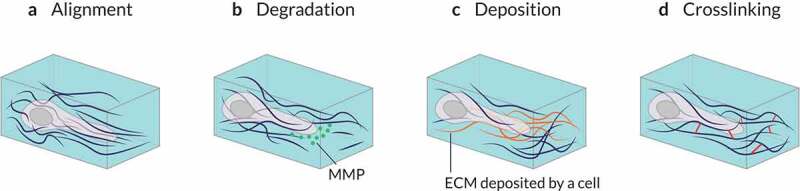
**Matrix remodeling through cell–matrix interactions.** (a) Mechanical forces exerted by cells can structurally remodel the surrounding matrix by stretching and aligning fibers of the extracellular matrix (ECM). (b) Cells may biochemically degrade a surrounding ECM by secreting various types of matrix metalloproteinases (MMPs). (c) Specific types of cells, such as fibroblasts, deposit additional ECM components on the surrounding matrix. This can lead to elevated matrix stiffness and smaller matrix pore size. (d) Cells can cross-link matrix fibers, resulting in the enhanced stiffness and elasticity of the ECM.

During migration, cells exert contractile forces to the ECM through focal adhesions, resulting in fiber alignment (Figure 8a) [95,148,192,258]. For example, after migrating toward the injured area through chemotaxis, fibroblasts bring wound edges together by exerting pulling forces to their surroundings. Alignment in ECM fibers and microenvironment topography modulate, among others, the PI3K signaling pathway and promote cytoskeletal remodeling and cell polarization [258]. Interestingly, Matrigel-containing hydrogels increase alignment anisotropy around cells *in vitro*. The alignment of ECM fibrils allows for long-range communication between cells during angiogenesis and tissue repair. Fiber alignment also enhances the invasion of tumor cells [259]. The pushing and pulling behaviors of cells such as fibroblasts or human mesenchymal stem cells also induce ECM stiffening by fiber compaction [95,148,259,260]. Substrate deformation gradually increases along a single axis during fibroblasts migrating in 3D domains, with higher and lower deformations at the leading and trailing edges, respectively [261]. Note that HT-1080 cells also exhibited this high frontal substrate prestrain found in fibroblasts. MDA-MB-231 cells, on the other hand, showed very similar displacements at the leading and rear edges. Moreover, during initial cell spreading within 3D matrices, fibroblasts seem to transmit anisotropic strain to the ECM to polarize. Chaudhuri and colleagues [34] highlighted the importance of matrix stress relaxation – which has recently been established as a key requirement for robust cell migration on soft substrates [187] – in cell–matrix interactions. Other works have shown a more versatile ECM because of the heterogeneity in crosslink unbinding kinetics [258,262]. Predominantly permanent crosslinks increase tension sustainability. Conversely, high levels of transient crosslinks increase plastic remodeling (i.e., nonelastic densification) during cell–matrix interactions. Therefore, a shift in the balance between permanent and transient crosslinks will bias ECM response to contractile cells. Other studies have shed some light on alternative strategies that facilitate cancer cell protease-independent invasion of basement membranes mediated by matrix mechanical plasticity [263,264]. Cells’ ability to mechanically remodel their surroundings is thus fundamental to many biological processes, including cell migration [2].

#### Degrading the ECM through cellular proteolytic activity

Cells can also degrade the surrounding environment and expand ECM pores by releasing MMPs (Figure 8b) [95,265]. The properties of the microenvironment, including substrate composition, may influence cells’ ability to align ECM fibers. For instance, in Matrigel-containing hydrogels, H1299 cancer cells require a more intense MMP activity to migrate than in collagen-only substrates. The former are stiffer and with fewer but larger pores than the latter. Matrix metalloproteinases act on several extracellular proteins (e.g., cytokines, antimicrobial peptides). Accordingly, they regulate, among others, different aspects of inflammation and immunity. Interestingly, Cdc42 and RhoA participate in MMPs trafficking to invadopodia tips [140,171]. Cytoskeletal dynamics may also modulate MMPs transport [208]. A dysregulated MMP activity is also associated with cancer, fibrosis, and cardiovascular disease [3]. Besides MMPs, cells can use other proteases such as adamalysins and meprins to biochemically break down ECM components [104]. They do so by catalyzing proteolysis, which breaks down proteins into smaller polypeptides or single amino acids. Notice that, in the absence of proteolytic activity, and if the matrix is viscoelastic enough, cells may deform and expand nanometer-size pores and migrate through [32]. Further, after receptor-mediated internalization, endocytic cargo degradation enables cells to internalize and degrade ECM molecules in lysosomes. Indeed, collagen internalization is considered a key protection mechanism in liver fibrosis *in vivo* [266,267]. Moreover, PI3K products have a role in membrane tension, influencing the endocytic response and membrane trafficking used by migrating cells (e.g., fibroblasts and neutrophils) [268]. Mechanical forces may alter the structure of some proteins, inhibiting/facilitating their interactions with the surrounding molecules [269]. As a result, strain suppresses the degradation of some ECM proteins, such as collagen [270]. Therefore, cells’ ability to degrade the surrounding substrate is tension-dependent and involved different players, such as proteases and lysosomes.

#### Regulating ECM composition by synthesis, secretion, deposition, and cross-linking of ECM components

Cells also regulate ECM composition by depositing and cross-linking some of its components (Figures 8c,8d). For instance, during morphogenesis, epithelial cells synthesize components of the basement membrane, such as collagen IV and laminin. Osteoblasts secret different ECM components (e.g., osteocalcin, osteopontin) during bone formation [271]. In the interstitial matrix, fibroblasts deposit several distinct ECM components within intact and wounded tissues [104]. Note that some of these secreted ECM proteins provide cell growth factors and cytokines, which may promote a chemotactic response. Other deposited components serve as physical scaffolds or mechanotransducers, promoting fibrils formation from collagen and fibronectin, and their cross-linking by enzymes. An example of ECM-modifying enzymes, lysyl oxidases (LOXs) covalently cross-link collagen fibrils, which is fundamental for the correct assembly of collagen fibers [1,270,272]. Tissue Transglutaminase (TG2) cross-links other ecm molecules, including fibronectin and collagen IV. Lysyl oxidases and TG2 are frequently overexpressed in cancer, increasing fibrosis, ECM stiffness, and cross-linking [87,89]. Further, they promote tumorigenesis, metastasis, and affect mechanical properties and cell–matrix signaling. By depositing viscoelastic ECM components, cells can also remodel the surrounding microenvironment and promote cell migration in elastic degradable substrates [32]. Recent studies have shown that collagen endocytosis can also support fibril assembly at the plasma membrane [273]. Aberrant overexpression of growth factors such as TGFβ, PDGF, and VEGF may be associated with different pathologies [3,119]. For instance, TGFβ overexpression promotes myofibroblasts differentiation, cell proliferation, and matrix production. At the same time, TGFβ signaling inhibits proteolytic activity, driving ECM stiffening. In response to substrate stiffening, cells exert higher contractile forces against the surrounding environment, which activates matrix production [274]. Moreover, by leveraging ECM remodeling through cell–matrix interactions, tumors create microenvironments that promote tumorigenesis and metastasis [272]. Interestingly, TGFβ controls many aspects of primary tumor growth and dissemination by inducing EMT and EMT-associated changes [58].

#### Interactions between different ECM remodeling mechanisms

Tissue homeostasis requires a balanced synthesis and degradation of structural proteins. Abnormal composition of the ECM because of the failed regulation of some of these processes is associated with different pathologies, such as fibrosis and metastasis [52,275]. For example, during the wound healing response fibroblasts end up undergoing apoptosis or become quiescent. However, during cancer and fibrotic diseases, the fibroblast response is sustained [3]. Fonta and colleagues [276] recently showed that in progressive diseases (e.g., cancer, viral infections of lymph nodes), tensional tissue homeostasis is perturbed by cell–matrix interactions. More recently, Perestrelo and colleagues [277] provided a comprehensive description of the changes in collagen network organization during pathological cardiac ECM remodeling. They also showed that underlying this reorganization, in cardiac fibroblasts, YAP is activated to rearrange the substrate in a profibrotic feed-forward loop. Note YAP activity also promotes the transcription of genes involved in cell–matrix interactions, ECM composition, and cytoskeleton integrity [133,234,278]. In summary, cells can interact with their surrounding microenvironment through a variety of mechanisms. Such cell–matrix interactions are fundamental for many cellular functions, including cell migration.

## Computational models of cell migration

Computational models can help overcome some of the challenges of experimental research and advance the understanding of complex biological processes such as cell migration. Unraveling the intricacies of some of these mechanisms is getting increasingly expensive. It requires costly equipment and highly qualified professionals. Furthermore, as computational power and data storage capabilities increase, so does the use of *in silico* modeling tools. Mathematical models may offer valuable insights more efficiently, for example, by more easily isolating some specific mechanisms and behavioral patterns. They could even act as advisors and consultants for experimental researchers by fostering new hypotheses to be tested at the lab.

Over the last several decades, the research community has developed a wide variety of *in silico* models, aiming to further our knowledge on cell migration. Most of the mathematical models proposed are focused on cells migrating on flat surfaces, which is what we know best so far [279–288]. However, there is an increasingly large number of *in silico* models replicating the more complex and physiologically relevant migration within 3D matrices [289–293]. Nonetheless, some of these computational models are 2D representations of 3d cell migration [294–296], or model a 3D cell moving on a flat substrate [297]. As a result, such works cannot replicate some hallmarks of cell motility within 3D matrices.

Next, we will present different *in silico* models classified according to different criteria based on the mode of migration, the scale, and the modeling approach.

### Investigating different modes of migration

Mathematical models of cell motility can be classified according to the migratory strategy used by simulated cells (e.g., individual or collective). Sun and Zaman [298] reviewed models of cell migration and cytoskeletal dynamics associated with this cell motility. The authors analyzed differences between amoeboid and mesenchymal migration, as well as individual versus collective migration. Shatkin and colleagues [299] reviewed different theoretical approaches used to consider how the biophysical properties of the ECM modulate cell migration. In particular, they focused on mathematical models that improved our understanding of metastasic behaviors and durotaxis. The authors also reviewed *in silico* models of mesenchymal and amoeboid migration. Interestingly, the authors noted that none of the models included in their review considered all the variables involved in cell motility, which would likely be infeasible.

#### Individual migration

Some *in silico* models have tried to shed some light on individual cellular motility – which, for instance, allow leukocytes to patrol tissues looking for pathogens. In these cases, and depending on distinct factors (e.g., cell type, the properties of the environment), cells can use different migrating strategies.

Fibroblasts – which are essential for maintaining connective tissue homeostasis and tissue repair – are usually considered the prototypical mesenchymal cell. These are thin and elongated cells that migrate using protrusive structures that adhere to the ECM through numerous, robust, and dynamic focal adhesions. They also rely on MMPs proteolytic activity to degrade the surrounding ECM, expanding the pores through which they squeeze themselves. Myosin expression, which maintains polarized substrate prestrain during migration, is another essential component of the mesenchymal phenotype [261]. Note, however, that distinct cell types exhibit different degrees of mesenchymal features.

Different numerical works focused on modeling mesenchymal migration within 3D matrices [300–302]. For instance, Heck and colleagues [294] developed an *in silico* model of cells migrating through a degradable viscoelastic ECM. This computational model enabled them to provide new insights regarding the role of protrusions in this mode of migration. Bangasser and colleagues [98] proposed a model that predicted an optimal ECM stiffness for mesenchymal cell migration. Interestingly, altering the number of active molecular motors and clutches could shift this stiffness optimum. Afterward, the authors verified this prediction experimentally.

During amoeboid-like migration, cells have a limited proteolytic capacity and largely reduced adhesion that hinders their ability to pull and rearrange ECM fibers.

Several computational models focused on this protease-independent migration strategy [303]. For instance, Moure and Gomez [304] presented a computational model of amoeboid cells chemotaxing on 2D surfaces and within 3D matrices. Their modeling efforts unveiled an intricate interaction between the dynamics of chemotactic ligands and the geometry of the substrate. Such interplay would tightly regulate cell migration. Campbell and Bagchi [305] proposed an *in silico* model that predicted that cell deformability and protein diffusivity would alter swimming behavior and speed. In particular, increasing the former would increase the speed of migration and switch from a random to a persistent unidirectional motion.

Cells can also move within 3D environments using a lobopodial mode of migration [4,7,306].

Although this mode of migration was more recently proposed, a few *in silico* models already focus on it. Serrano-Alcalde and colleagues [307] presented a computational model to shed some light on the factors and mechanisms activating this mode of migration. Through finite element modeling, the authors identified two possible mechanotransduction mechanisms that may regulate the switch from mesenchymal to lobopodial migration: the fluid flow velocity inside the cytoplasm and the pore pressure.

#### Collective models

Cells may also interact with their neighbors through cell–cell adhesions (e.g., tight junctions, cadherin-based adherens junctions, desmosomes). Collective migration is associated with development, regeneration, and tissue repair. During these events, cells can move as sheets adhered to the surrounding ECM. Tumoral cells also use this mode of migration while invading as sheets at the interface between tissues.

This cooperative mode of migration has been extensively studied using *in silico* models. Indeed, Alert and Trepat [308], as well as Camley and Rappel [309], recently reviewed the physical models developed by the research community to explain collective cell migration. Deutsch and colleagues [310] proposed BIO-LGCA, a cellular automaton, to analyze this mode of migration to predict the formation of clusters in adhesive interacting cells. Garcia-Gonzalez and Muñoz-Barrutia [311] were interested in studying how substrate stiffness influences collective migration. They developed a model to test different hypotheses regarding which mechanisms drive collective motion. The authors suggested that the main driver of non-symmetric collective motility is the induced cell polarization by substrate stiffness gradients. Notably, Mayalu and colleagues [312] superposed single-cell computational models to predict multicellular behaviors. Neumann and colleagues [313] integrated experimental and computational data to create an *in silico* model of tube elongation. This computational model revealed that mammary morphogenesis can emerge by combining intercalation, interfacial tension dynamics, and high basal stress. Escribano and colleagues [314] developed a computational model that enabled them to compare single and collective migration. Their *in silico* model helped them understand why collective motion is much more efficient than single-cell migration.

### Investigating at different scales

Mathematical models can also be classified according to their scale (i.e., subcellular-, cellular-, and tissue level) [315,316]. Of note, Buttenschon and Edelstein-Keshet [317] recently reviewed multi-scale models, coupling events from the intracellular to the cellular to the multicellular scales. Ferruzzi and colleagues [33] examined the experimental and modeling techniques available to study the structure and multi-scale mechanics of collagen networks. Conversely, Spill and colleagues [318] reviewed models and supported experimental findings of different aspects of mechanobiology – which are also related to cell migration – spanning different scales. Lastly, Cheng and colleagues [319] reviewed models from different scales proposed to improve our knowledge of how cells respond to biophysical stimuli.

#### Subcellular models

Subcellular models have tried to shed some light on specific processes involved in cell migration that may occur in some cellular regions. For instance, Borau and colleagues [320] focused on the mechanosensing properties of the actomyosin network. Fatunmbi and colleagues [321] focused on the recruitment of actin nucleating proteins at the membrane interface. In contrast, Hetmanski and colleagues [322] proposed a combination of distinct modeling approaches to study rear retraction dynamics of migrating cells within 3D substrates. Hobson and Stephens [323] reviewed the mechanical modeling of cell nuclei. Regarding chemotaxis, Hopkins and Camley [324] recently used *in silico* modeling to study cells’ ability to accurately process external signals in uncertain environments. They argue that cells should adapt their cell surface receptor expression based on the surrounding environment. In particular, cells should only express multiple receptor types if they typically explore environments where ligand concentrations vary over orders of magnitude. Karagoz and colleagues [325] reviewed the computational models of integrin signaling. Also, LeRoux and colleagues [82] included a review of different mathematical models proposed to improve our knowledge of the impact of mechanical stimuli on the plasma membrane and its complex mechanochemistry. Conversely, Oria and colleagues [131] proposed a general framework to explain how cells sense spatial and physical information at the nanoscale. They combined *in vitro* observations with a computational molecular-clutch model, in which individual integrin-matrix bounds respond to force loading by recruiting additional integrins (up to a maximum value). Interestingly, their results showed that, contrary to the by-then consensus, an increase in substrate stiffness or ligand density promoted adhesion growth. Lastly, Vignaud and colleagues [200] built a biophysical model to investigate the properties of an elastic network of actin fibers embedded in a cortical meshwork. One of the main novelties of this work was that stress fibers were not connected to the ECM but the adjacent cortical meshwork.

#### Cellular models

Cellular models may be interested in combining some of the aforementioned biological events to explain distinct aspects of cell motility and simulate the entire cell. For instance, Adebowale and colleagues [187] developed an *in silico* model that was able to replicate several observed experimental trends. First, how stress relaxation on viscoelastic substrates and stiffness on elastic ones influence cell migration speeds. Secondly, the impact of inhibition of adhesion, actin polymerization, and actomyosin contraction. In contrast, Cao and colleagues [297] proposed a computational model of cell migration integrating two continuum models: a biochemical activator-inhibitor system coupled with cell mechanics (cell membrane deformation and cell motion). Merino-Casallo and colleagues [326] developed an *in silico* model of 3D cell migration that integrated intracellular signaling with cell mechanics that replicated some of the main observations of *in vitro* experiments under different biochemical profiles. Also, Li and colleagues [291] proposed a 3D model of breast cancer cell migration, in which they included distinct modulating factors, such as fluid dynamics, autologous chemotaxis, substrate rigidity, and fibrillar structure, as well as cell-fiber and cell-flow interactions. Lastly, Moure and Gomez [327] recently studied the influence of myosin activity on cell polarization and how mechanical cues induce motion. In particular, their *in silico* model for keratocytes considered cell deformations, myosin-RhoA dynamics, and forces associated with the actomyosin network.

#### Tissue-level models

Tissue-level models represent collective cell motility. For example, Gonzalez-Valverde and Garcia-Aznar [328] proposed a hybrid model to simulate collective cell migration in epithelial monolayers. Nosbisch and colleagues [329] developed a framework that enabled them to couple signal transduction mechanisms at the molecular level to individual and collective migration guided by chemoattractant gradients in tissues. Conversely, Peng and colleagues [330] proposed a multi-scale model of tumour invasive growth. This model considered the active interplay between the molecular mechanics of some proteolytic enzymes at the cell scale and the tissue-scale tumor dynamics. Sunyer and colleagues [79] found that multicellular clusters exhibited durotactic behavior – even if their isolated constituent cells did not durotax – because of supracelllular transmission of contractile forces. To explained the observed phenomenology, the authors proposed a continuum model integrating clutch-like cell–matrix dynamic at focal adhesions, long-range force transmission through cell–cell junctions, and actin polymerization at monolayer edges. Notably, Fletcher and Osborne [331] recently reviewed the progress in multi-scale modeling of multicellular tissues. They also highlighted some ongoing challenges associated with their definition, implementation, and validation.

### Investigating through different modeling approaches

Mathematical models may also be classified depending on the modeling approach used (continuum, discrete, or hybrid) [316,332].

#### Continuum models

Continuum models are based on the definition of constitutive laws to model processes and events (e.g., transport of biochemical substances, actomyosin contraction, or nuclear deformation). They rely on solving partial differential equations. The finite element method and other derived methods (e.g., smoothed-particle hydrodynamics) are some of the most applied techniques [251 294,333,334]. Other authors have opted for the phase-field model [335]. These models have been extensively used to reproduce large-scale biological systems. However, as the number of biological processes included in these models increases, so does the complexity of the defined constitutive laws. For example, Ahmadzadeh and colleagues [336] developed a continuum model to determine how cells collaborate to elongate epithelial tubes. In this model, the authors included different aspects of cell migration, including cell adhesions, substrate rigidity, fiber realignment, strain stiffening, ECM ligand density, and pore size. In contrast, Arefi and colleagues [333] developed a finite-element model to simulate the extravasation process. They included the chemo-mechanics of the stress fibers and focal adhesions, as well as the contractile forces pulling the nucleus of tumor cells against the elastic resistance of the endothelial cells. Banavar and colleagues [337] focused their attention on the role of genetically encoded mechanical feedback as a coordinator of cell morphogenesis and polarity. Also, Bennett and colleagues [338] developed a continuum model to explain the DNA damage occurring during constricted migration. Hervas-Raluy and colleagues [334] focused on the effects of actin and myosin in cell motility within confined environments, considering the different mechanical properties of the cytoplasm and the nucleus. Notably, Lee and colleagues [290] presented a combined *in silico* and *in vitro* model of macrophages migrating within 3D matrices in response to biophysical and biochemical factors. They coupled chemokine- and intermediate filament-mediated signaling cascades commonly regulated by Rho GTPases. Mackenzie, Rowlatt, and Insall [339] presented a finite element method to approximate systems of bulk-surface reaction-diffusion equations on 2D domains. They also used the proposed methodology to model individual migration guided by chemotaxis. Conversely, Moure and Gomez [335] reviewed phase-field models of individual and collective migration. Mukherjee and colleagues [251] used a continuum model to analyze the evolution of nuclear shape and stresses during the confined migration of a cell through a deformable ECM. Lastly, Serrano-Alcalde and colleagues [340] developed a continuum model to study the role of nuclear mechanics in cell deformation under different creeping flows.

#### Discrete models

In discrete models, the different agents involved are portrayed as separate units in the system. Therefore, it is more direct and intuitive to represent the spatial inhomogeneities and variability of biological systems. As a result, we can include more information in those models. Historically, discrete models were computationally expensive as they are representing every agent as an independent unit. They must also consider how those units interact with each other. However, computational costs have greatly decreased during the last several decades, which has dramatically alleviated this issue. Besides, the open-source community offers an increasing number of applications and libraries based on the discrete approach (e.g., PhysiCell, FLAME) [341,342].

Different authors have proposed agent-based models related to cell migration. For one, Feng and colleagues [343] integrated signaling networks, integrin dynamics, and substrate stiffness in a mechanochemical model of neutrophil migration. Reinhardt and Gooch [344] proposed a model focused on cell–matrix interactions. In particular, they studied the impact of different biophysical features of the substrate in ECM remodeling. Also, Drasdo, Van Liedekerke, and colleagues [345,346] focused on different discrete modeling approaches (lattice and off-lattice) to simulate different biological processes, including cell migration. Lastly, PhysiBoSS is a multi-scale agent-based modeling framework that combines intracellular signaling and multicellular behavior [347].

#### Hybrid models

Hybrid models combine continuum and discrete models to overcome their intrinsic limitations. Designing the interface between those models is their main issue, as they must share information with each other.

Different works have proposed a hybrid approach to replicate some of the biological processes associated with cell migration. For example, Gonçalves and Garcia-Aznar [348] proposed a hybrid model to simulate how the ECM density regulates the formation of tumor spheroids through cell motility. They modeled cells using a discrete center-based framework while a continuum model defined the ECM. Also, Gonzalez-Valverde and Garcia-Aznar focused on understanding how forces at cell–cell contact sites and the rigidity of epithelial monolayers modulate collective migration and topology [349]. In this case, an agent-based model defined cells whereas a continuum material model described the cell passive mechanics. Macnamara and colleagues [350] presented an *in silico* model to simulate cancer growth and migration within a 3D heterogeneous tissue. They used an agent-based model to simulate the behavior of cells and the tempo-spatial interactions between each other. The authors coupled this model to a finite-element solver to model the diffusion of oxygen from blood vessels to cells. Rens and Merks [280] proposed a hybrid model to explain the full range of cell shape and durotaxis from focal adhesion dynamics. They used an agent-based lattice model to represent cells. However, they calculated the planar stress in the ECM using a continuum model where they represented the substrate with a finite-element model. Lastly, Sfakianakis, Madzvamuse, and Chaplain [351] proposed a hybrid multi-scale model to describe cancer invasion of the ECM.

## Conclusions and future perspectives

Researchers are extensively expanding our knowledge of fundamental processes such as cell migration, shedding light on some of the principles that lay in the dark until very recently. Still, there is much to be investigated about the different processes involved in cell migration. For instance, despite intense research since the 1970s, many of the most fundamental questions about RTK and GPCR activity are still unanswered [68,352]. During cell migration, different surface receptors trigger a variety of signaling pathways. The Rho gtpases family, which consists of 20 members in humans, plays a significant role in many of these pathways [145]. The traditional view, based fundamentally on evidence gathered in 2D assays, portrayed a very simple Rho GTPase network. This view proposed a Rac1-dominated leading edge for promoting protrusions formation and a RhoA-dominated rear for actomyosin contractility [140]. However, more recent studies about 3D domains have revealed that these Rho networks are much more complicated [140,171]. Distinct mechanisms regulate Rho GTPase signaling. The activation of these mechanisms, in turn, is usually mediated by roughly 70 different RhoGEFs and 80 distinct RhoGAPs [146,171,353]. Such regulatory events are likely to be context-dependent, varying among cell types and in response to different signals. Therefore, our understanding of the Rho GTPase regulation in particular – and other signaling pathways, such as the Hippo, in general – is still far from complete [354,355].

Mechanosensors enable cells to sense and transduce the physical stimuli they are exposed to. For instance, the forces generated by neighboring cells, blood flow, or in confined interstitial spaces [356]. The magnitude, direction, and temporal dynamics of these forces influence the cellular responses. We are just beginning to uncover how cells get this spatiotemporal information. Cells’ ability to extract this information builds upon the specific sensitivities of distinct mechanosensors and their underlying transduction mechanisms. We also lack a comprehensive understanding of the heterogeneous nature of IACs and their varied functions [122]. Further research should also focus on how the hundreds of IAC-associated proteins form the complex network of interactions within these adhesive complexes.

More studies are required to develop a comprehensive knowledge of the influence of each cytoskeletal component over the events associated with cell migration, and how the interactions between them regulate this migratory process [206,208]. For example, it is still unclear if the role of filopodia is more related to sensing, or its structure acts as a force-generating/bearing player [189]. Emerging evidence points toward different subtypes of filopodia fulfilling these distinct roles, based on context. Actin polymerization can also direct proteolytic activity by accumulating actin microfilaments at sites of ECM contact, where it promotes the recruitment of specific MMPs. A more thorough understanding of how the distinct actin microfilament networks compete for actin monomers as well as the cooperation and competition among actin-binding proteins is also required [169]. Yang and colleagues [357] recently developed a novel proteomics-based approach to identify polarity regulators. As a result, they identified Leep1, which regulates activities at the leading edge, including actin dynamics. Interestingly, the actin-binding protein Cofilin-1, but not ADF, was found to affect cell stiffness [182]. Furthermore, new details of cellular crosstalks that originated in the cytoskeleton may be uncovered, highlighting its regulatory role in cell signaling pathways [358]. We still lack detailed knowledge of how the spatiotemporal signaling variations influence actin cortex reorganization at the molecular level and the mechanical changes happening at the cellular scale [188]. More research about the actin/adhesion crosstalk is also required [359]. Buracco and colleagues [178] argued that we still lack a coherent picture of the global mechanisms that induce a heterogeneous actin distribution within cells.

There are still unanswered questions about the interactions between the cytoskeleton and the nucleus too [21,240,247]. For instance, how does the LINC complex sense and transduce forces? Preliminary reports from Newman and colleagues [151] have recently shed some light on this issue. In particular, the authors showed nuclear force transduction between IACs and the LINC complex in invasive migrating cells within 3D environments.

Recently, different works have pointed toward phase separation, a particular form of biological phase transition involved in cytoskeletal and signaling dynamics [360,361], having a regulating role in dynamic processes happening at the front of migrating cells [362]. Phase separation adds an extra layer of localization and nonlinearity [176]. Besides, it is extremely hard to measure *in vivo*, and its importance is yet to be confirmed.

Our knowledge of cell–matrix interactions in 2D is relatively comprehensive. Still, studies must be translated to 3D *in vivo* scenarios because of the stark differences between these two systems regarding their biochemical and biophysical properties [171]. For instance, we still do not know much about the composition, regulation, and role of IACs in 3D domains [122,,151]. New protocols are required to increase our knowledge of the mechanisms of cell–matrix interactions in these more realistic environments [33,236]. Novel techniques to increase the resolution of our observations are also required. For example, to monitor the space between the plasma membrane and the actin network [180] and visualize the structure and dynamics of integrin-mediated adhesions in 3D [113]. Recent studies even suggest that focal adhesions may not act as a universal biophysical sensor across all cell types [121]. Other authors showed that the local plasma membrane curvature at the cell–ECM interface acts as a nanotopography sensor [82,121].

Some of the findings mentioned in this review and others not included here may not translate to 3D (*in vitro* and *in vivo*) systems. In particular, those about cytoskeletal and signaling dynamics. For instance, Sarkar and colleagues [363] showed that, on flat substrates, cells create an ECM ligand gradient, promoting persistent cell motility. Nonetheless, as pointed out by Elosegui-Artola and Oria [364], do these novel findings happen in more physiologically relevant environments? Shellard and Mayor [365] also noted that the relevance of durotaxis *in vivo* is still unclear. Partially, at least, because of the current challenges associated with *in vivo* research. Therefore, we must keep improving our ability to control and measure distinct physiologically relevant features so that *in vitro* studies replicate more accurately *in vivo* conditions. Quantifying these features *in vivo* is even more difficult [80], and thus, more challenges lie ahead on this front.

Historically, the research community has tried to simplify experiments and models to make them understandable [176]. Interest in complex phenomena (e.g., nonlinear responses, feedback loops, and competition between distinct components) has recently emerged [169,366,367]. As a result, we may discover behaviors that would only emerge from such complexity.

Cell migration is an extremely complex phenomenon. A myriad of biological processes may participate in cell motility. Nevertheless, they might play different roles based on the specifics of any given scenario. This multi-modal nature of cell migration enables tumor cells to evade the targeted inhibition of specific pathways involved in cell motility. Notably, Hapach and colleagues [368] recently showed that E-cadherin, generally considered a tumor suppressor, enables breast cancer metastasis. Therefore, it is one of the main challenges in developing effective cancer therapies [369]. Studying specific components and players in isolation and on a given scale may still be helpful. Nevertheless, it is imperative to analyze them at different scales as well as their interactions with each other [176,229,367,370]. For instance, mechanosensing must happen in milliseconds, while protein modifications mediating mechanotransduction occur in seconds to minutes, and the associated transcriptional responses require minutes to hours to happen [23]. Accordingly, novel protocols, methodologies, and techniques for quantitative analysis of these components and events are still required.

We may also expand our focus when studying cell motility. For instance, the initial engineered cell–hydrogel interface is not the only factor influencing cellular outcomes in *in vitro* assays, at least in cell signaling studies [371]. Instead, researchers should also consider other factors, such as the adhesion to and remodeling of nascent proteins already deposited by cells soon after culture initiation. Not until very recently have we started exploring the impact of ECM viscoelasticity in cell behavior [32]. Hence, there are still substantial gaps in our knowledge of the role of viscoelasticity and viscoplasticity in cell motility. Whatsmore, we still need to fully understand how other biophysical factors, such as the stiffness and the microarchitecture of the ECM, influence migratory behaviors. Phillip and colleagues [372] recently study single-cell motility patterns of primary dermal fibroblasts from healthy donors. Their results demonstrated an age-associated decrease in overall cell migration and cellular heterogeneity. They also found a prevalence of lesser motile phenotypes in older individuals. Other recent studies have focused on investigating how metabolic heterogeneities influence cell migration [373]. Interestingly, some of the chemical and mechanical factors mentioned in this review, such as matrix density, stiffness, fiber alignment, and confinement, influence metabolic plasticity during cancer cell migration [374,375]. Energy demands are specific to any given microenvironment. Therefore, such metabolic and migratory plasticity is essential for the successful invasion and migration of cancer cells. For example, cells migrating in denser matrices require more energy for actin cytoskeletal remodeling and actomyosin contractility [60]. Matrix stiffening also induces microtubule glutamylation, which has been shown *in vitro* and *in vivo* to be necessary and sufficient to promote breast cancer cell invasion [207]. Furthermore, the mode of migration heavily biases the migratory potential and metabolism of migrating cells. However, we still cannot monitor and measure energy production and consumption in real-time *in vivo*. Neither can we interrogate metabolic activity in cancer progression with cellular-level resolution using clinical imaging.

*In silico* models should be considered as a powerful prediction tool. For instance, mathematical modeling could predict how Rho GTPases would signal in response to perturbations of the signaling networks they interact with [171]. Modeling formalisms, such as Guarded Flexible Nets, may assist during the study of signaling networks [376]. Sometimes, we are interested in how just a handful of species from a complex and large-scale network evolve. In such cases, surrogate-assisted approaches for model reduction could also be helpful [377]. Also, Hellander and Hellander developed an algorithm that couples mesoscopic simulations in Cartesian meshes with different granularity [378]. As a result, they could save up to three orders of magnitude of computational time without losing significant accuracy.

As our ability to collect data increases, the integration of machine learning and multi-scale modeling seems extremely powerful. Indeed, Alber and colleagues [379] demonstrated that machine learning and multi-scale modeling complement each other. The processes studied by and the models developed by the scientific community are increasing their complexity. These works required the use of more complex and costly equipment by more specialized technicians. By automating some of these workflows, we can enhance their efficiency (minimizing errors, standardizing protocols and procedures, and scaling them up). Otherwise, our efforts to make new and compelling discoveries may be hindered.

Bayesian optimization, bioimage analysis, and smart microscopy are techniques that exemplify this new trend. Bayesian optimization is a general-purpose black-box optimization methodology [380,381]. By using principles of statistical inference and decision theory, Bayesian optimization efficiently finds the global optimization of expensive-to-evaluate objective functions. Thus, Bayesian optimization is especially suitable for the experimental design and calibration of expensive processes [382–385]. Notably, Bayesian optimization allows us to perform model calibration, avoiding the usual grid search, using a mixture of exploration and exploitation approaches in a completely hands-free process. Further, Bayesian methodologies may be appropriate even when the optimal parametrization is input dependent [386]. They may even be suitable when the optimal parametrization is not constant over time [387].

On the other hand, bioimage analysis includes a myriad of methods and techniques for different purposes, such as object detection, localization, and segmentation [388–391]. Some of these procedures are based on artificial intelligence. For instance, convolutional neural networks in particular, and deep learning algorithms, in general, have been widely used for image recognition [392–395]. By automating image processing and analysis, experimental researchers and technicians can boost their productivity. It may drastically reduce the time spent by these professionals processing the data generated during their experiments. This automation could also improve the statistical significance of their findings by increasing the number of experiments performed. Experimentalists might even evaluate different hypotheses and study distinct scenarios more quickly. Notably, researchers have gone a step further with the emerging field of computer-assisted imaging known as smart microscopy [396]. Algorithms analyze the incoming data and guide the instrument accordingly (e.g., which events to image, how to do it, or compensating for any optical or physical noisy perturbations). For example, the AutoPilot platform is a light-sheet microscopy framework for spatiotemporally adaptive live imaging [397]. Still, smart microscopy is just an example of a broader movement focused on using computational techniques to make the most out of imaging experiments. Machine learning may help design better microscopy experiments [398–401], overcome limitations in imaging quality [402], and boost the performance of an instrument beyond the limits of its optics [403,,404]. Note that, as with any machine learning-based technique, users should always keep in mind that using well-trained neural networks is not enough to avoid bias. Therefore, implementing quality control measures and data audits is paramount so that appealing but inaccurate results do not mislead us.

In summary, a century of research in cell migration has allowed us to answer many (but not all) of the emerging questions about cell migration. A comprehensive understanding of how cells probe and respond to the surrounding microenvironment would greatly improve our quality of life. For example, it would enable us to create synthetic organs and find the cure for some of the leading causes of death worldwide. Developing new methods and techniques to increase the scale and resolution of our experimental analyses is essential to uncover some of the remaining mysteries that lie ahead. *In silico* modeling has proven fundamental to advance our knowledge in many fields, including cell biology and motility. The integration of other computational tools (e.g., machine-learning, Bayesian optimization, bioimage analysis) in our workflows has demonstrated to be a very promising venue in our quest for a complete and detailed picture of cell migration.

## Abbreviations

**1D** One-Dimensional**2D** Two-Dimensional **3D** Three-Dimensional**AMT** Amoeboid to Mesenchymal Transition**Arp2/3** Actin Related Protein 2/3**CAT** Collective to Amoeboid Transition**Cdc42** Cell Division Control Protein 42 Homolog**DNA** Deoxyribonucleic Acid**EAT** Epithelial to Amoeboid Transition**ECM** Extracellular Matrix **EGF** Epidermal Growth Factor**EGFR** Endothelial Growth Factor Receptor**EMT** Endothelial to Mesenchymal Transition**Ena/VASP** Enabled/Vasodilator-stimulated Phosphoprotein**FAK** Focal Adhesion Kinase**GAP** GTPase-activating Protein**GEF** Guanine Nucleotide Exchange Factor**GPCR** G Protein-coupled Receptor**GTPases** Guanosine Triphosphatases**IAC** Integrin Adhesion Complex**LINC** Linker of Nucleoskeleton and Cytoskeleton**LOX** Lysyl Oxidase**MAT** Mesenchymal to Amoeboid Transition**mDia1** Diaphanous-related Formin-1**mDia2** Diaphanous-related Formin-2 **MEC** Mammary Epithelial Cells**MMP** Matrix Metalloproteinases**mRNA** Messenger Ribonucleic Acid**MSC** Mesenchymal Stem Cell**MT1-MMP** Membrane Type 1 Metalloprotease**MTOC** Microtubule-organizing Center**PDGF** Platelet-derived Growth Factor**PI(4,5)P_2_** Phosphatidylinositol 4,5-bisphosphate**PI3K** Phosphoinositide 3-kinase(also called Phosphatidylinositol 3- kinase)**Rac1** Ras-related C3 Botulinum Toxin Substrate 1**RhoA** Ras Homolog Family Member A**RhoB** Ras Homolog Gene Family, Member B**RhoBTB1** Rho-related BTB Domain-containing Protein 1**ROCK** Rho-associated Protein Kinase**RTK** Receptor Tyrosine Kinase**Src** Proto-oncogene Tyrosine-protein Kinase Src**SUN1/2** Sad1 and UNC84 Domains Containing 1 and 2**TAZ** Tafazzin**TG2** Tissue Transglutaminase**TGFβ** Transforming Growth Factor Beta**VEGF** Vascular Endothelial Growth Factor**VEGFR** Vascular Endothelial Growth Factor Receptor**YAP** Yes-associated Protein

## References

[1] Karamanos NK, Theocharis AD, Piperigkou Z, et al. A guide to the composition and functions of the extracellular matrix. FEBS J. 2021;228:15776. 10.1111/febs.15776 33605520

[2] David ACW, Kenneth MY. The extracellular matrix in development. Development. 147(10):2020. 10.1242/dev.175596 PMC7272360 32467294

[3] Marsha CL, Cynthia AR-K. Targeting extracellular matrix stiffness to attenuate disease: from molecular mechanisms to clinical trials. Sci Transl Med. 10(422):2018. 10.1126/scitranslmed.aao0475 29298864

[4] Kenneth MY, Sixt M. Mechanisms of 3D cell migration. Nat Rev Mol Cell Biol. 2019;20(12):738–752. 31582855 10.1038/s41580-019-0172-9

[5] Eddy CZ, Raposo H, Wong R, et al. Morphodynamics facilitate cancer cells to navigate 3D extracellular matrix. Scientific Reports. 2020;11(1):1–10. 10.1038/s41598-021-99902-9. PMC8516896 34650167

[6] Zanotelli MR, Chada NC, Andrew Johnson C, et al. The physical Microenvironment of Tumors: characterization and clinical impact. In: Phys. cancer. World Scientific Publishing Company; 2020. p. 165–195. https://www.worldscientific.com/doi/abs/10.1142/9789811223495_0008.

[7] Yamada KM, Collins JW, Cruz Walma DA, et al. Extracellular matrix dynamics in cell migration, invasion and tissue morphogenesis. Int J Exp Pathol. 2019;100(3):144–152. 31179622 10.1111/iep.12329 PMC6658910

[8] Paul CD, Mistriotis P, Konstantopoulos K. Cancer cell motility: lessons from migration in confined spaces. Nat Rev Cancer. 2017;17(2):131–140. 27909339 10.1038/nrc.2016.123 PMC5364498

[9] Shellard A, Mayor R. Rules of collective migration: from the wildebeest to the neural crest: rules of neural crest migration. Philos Trans R Soc B Biol Sci. 2020;375:1807. 10.1098/rstb.2019.0387 PMC7423382 32713298

[10] De Pascalis C, Etienne-Manneville S, Weaver VM. Single and collective cell migration: the mechanics of adhesions. Mol Biol Cell. 2017;28(14):1833–1846. 28684609 10.1091/mbc.E17-03-0134 PMC5541834

[11] Friedl P, Mayor R. Tuning collective cell migration by cell-cell junction regulation. Cold Spring Harb Perspect Biol. 2017;9(4):a029199. 28096261 10.1101/cshperspect.a029199 PMC5378050

[12] Lintz M, Muñoz A, Reinhart-King CA. The mechanics of single cell and collective migration of tumor cells. J Biomech Eng. 2017;139(2):9. 10.1115/1.4035121 PMC5395914 27814431

[13] Pfeffer W. Locomotorische Richtungsbewegungen durch chemische Reize:(Aus den” Untersuchungen aus dem botanischen Institut zu Tübingen Bd. I. Heft 3 p. 363-482). W. Engelmann; 1884.

[14] Caton R. Contributions to the cell-migration theory. J Anat Physiol. 1870;5(Pt 1):35–420.7. PMC1318808 17230884

[15] Addison W. Experimental and practical researches on the nature and origin of tubercles in the lungs. Prov Med Surg J. 1842;1-4(20):403–407.

[16] Cosgrove BD, Loebel C, Driscoll TP, et al. Nuclear envelope wrinkling predicts mesenchymal progenitor cell mechano-response in 2D and 3D microenvironments. Biomaterials. 2021;270:120662. 33540172 10.1016/j.biomaterials.2021.120662 PMC7936657

[17] Harris MJ, Wirtz D, Pei Hsun W. Dissecting cellular mechanics: implications for aging, cancer, and immunity. Semin Cell Dev Biol. 2019;93:16–25. 30359779 10.1016/j.semcdb.2018.10.008

[18] Kim JE, Reynolds DS, Zaman MH, et al. Characterization of the mechanical properties of cancer cells in 3D matrices in response to collagen concentration and cytoskeletal inhibitors. Integr Biol. 2018;10(4):232–241. 10.1039/c8ib00044a PMC5916796 29620778

[19] Moure A, Gomez H. Dual role of the nucleus in cell migration on planar substrates. Biomech Model Mechanobiol. 2020;19(5):1491–1508. 31907682 10.1007/s10237-019-01283-6

[20] Pei-Hsun W, Gilkes DM, Wirtz D. The biophysics of 3D cell migration. Annu Rev Biophys. 2018;47(1):549–567.

[21] Zhu R, Liu C, Gundersen GG. Nuclear positioning in migrating fibroblasts. Semin Cell Dev Biol. 2018;82:41–50. 29241691 10.1016/j.semcdb.2017.11.006 PMC5995615

[22] Scott KE, Rychel K, Ranamukhaarachchi S, et al. Emerging themes and unifying concepts underlying cell behavior regulation by the pericellular space. Acta Biomater. 2019;96:81–98. 31176842 10.1016/j.actbio.2019.06.003

[23] Espina JA, Marchant CL, Barriga EH. Durotaxis: the mechanical control of directed cell migration. FEBS J. 2021. DOI:10.1111/febs.15862 PMC9292038 33811732

[24] Zhang F, Wang J, Lü D, et al. Mechanomics analysis of hESCs under combined mechanical shear, stretch, and compression. Biomech Model Mechanobiol. 2020;20(1):205–222. 32809130 10.1007/s10237-020-01378-5

[25] Kameritsch P, Renkawitz J. Principles of Leukocyte migration strategies. Trends Cell Biol. 2020;30(10):818–832. 32690238 10.1016/j.tcb.2020.06.007

[26] Bajanca F, Gouignard N, Colle C, et al. In vivo topology converts competition for cell-matrix adhesion into directional migration. Nat Commun. 2019;10(1):1–17. 30944331 10.1038/s41467-019-09548-5 PMC6447549

[27] te Boekhorst V, Preziosi L, Friedl P. Plasticity of cell migration in vivo and in silico. Annu Rev Cell Dev Biol. 2016;32(1):491–526. 27576118 10.1146/annurev-cellbio-111315-125201

[28] Geiger F, Rüdiger D, Zahler S, et al. Fiber stiffness, pore size and adhesion control migratory phenotype of MDA-MB-231 cells in collagen gels. PLoS One. 2019;14(11):e0225215. 31721794 10.1371/journal.pone.0225215 PMC6853323

[29] Chaudhuri O, Cooper-White J, Janmey PA, et al. Effects of extracellular matrix viscoelasticity on cellular behaviour. Nature. 2020;584(7822):535–546. 32848221 10.1038/s41586-020-2612-2 PMC7676152

[30] Paul CD, Hruska A, Staunton JR, et al. Probing cellular response to topography in three dimensions. Biomaterials. 2019;197:101–118. 30641262 10.1016/j.biomaterials.2019.01.009 PMC6390976

[31] Mak M, Anderson S, McDonough MC, et al. Integrated analysis of intracellular dynamics of MenaINV cancer cells in a 3D matrix. Biophys J. 2017;112(9):1874–1884. 28494958 10.1016/j.bpj.2017.03.030 PMC5425400

[32] Elosegui-Artola A. The extracellular matrix viscoelasticity as a regulator of cell and tissue dynamics. Curr Opin Cell Biol. 2021;72:10–18. 33993058 10.1016/j.ceb.2021.04.002

[33] Ferruzzi J, Zhang Y, Roblyer D, et al. Multi-scale mechanics of collagen networks: biomechanical basis of matrix remodeling in cancer. In: Stud. mechanobiol. tissue eng. biomater. Vol. 23. Springer; 2020. p. 343–387. https://link.springer.com/chapter/10.1007/978-3-030-20182-1_11.

[34] Chaudhuri O, Luo G, Klumpers D, et al. Hydrogels with tunable stress relaxation regulate stem cell fate and activity. Nat Mater. 2016;15(3):326–334. 26618884 10.1038/nmat4489 PMC4767627

[35] Hazur J, Endrizzi N, Schubert DW, et al. Stress relaxation amplitude of hydrogels determines migration, proliferation, and morphology of cells in 3-D. bioRxiv. 2021;10:270–280. 10.1039/d1bm01089a 34850787

[36] Hervas-Raluy S, Jose Gomez-Benito M, Borau-Zamora C, et al. A new 3D finite element-based approach for computing cell surface tractions assuming nonlinear conditions. PLoS One. 2021;16(4):e0249018. 33852586 10.1371/journal.pone.0249018 PMC8046236

[37] Gómez-González M, Latorre E, Arroyo M, et al. Measuring mechanical stress in living tissues. Nat Rev Phys. 2020;2(6):300–317. 10.1038/s42254-020-0184-6 PMC7617344 39867749

[38] Zhang J, Chada NC, Reinhart-King CA. Microscale interrogation of 3D tissue mechanics. Front Bioeng Biotechnol. 2019;7:412. 31921816 10.3389/fbioe.2019.00412 PMC6927918

[39] Duval K, Grover H, Hsin Han L, et al. Modeling physiological events in 2D vs. 3D cell culture. Physiology. 2017;32(4):266–277. 28615311 10.1152/physiol.00036.2016 PMC5545611

[40] Conti S, Kato T, Park D, et al. CAFs and cancer cells co-migration in 3D spheroid invasion assay. In Zhang, Yanhang: Methods mol. biol. Vol. 2179. Humana Press Inc; 2020. p. 243–256. https://link.springer.com/chapter/10.1007/978-3-030-20182-1_11#citeas. 10.1007/978-1-0716-0779-4_19 32939725

[41] Shelton SE, Tuan Nguyen H, Barbie DA, et al. Engineering approaches for studying immune-tumor cell interactions and immunotherapy. iScience. 2020;24(1):101985. 33490895 10.1016/j.isci.2020.101985 PMC7808917

[42] Szabó A, Mayor R. Mechanisms of neural crest migration. Annu Rev Genet. 2018;52(1):43–63. 30476447 10.1146/annurev-genet-120417-031559

[43] Roca-Cusachs P, Sunyer R, Trepat X. Mechanical guidance of cell migration: lessons from chemotaxis. Curr Opin Cell Biol. 2013;25(5):543–549. 23726023 10.1016/j.ceb.2013.04.010

[44] Carter SB. Haptotaxis and the mechanism of cell motility. Nature. 1967;213(5073):256–260. 6030602 10.1038/213256a0

[45] Harris H. Role of Chemotaxis in Inflammation. Physiol Rev. 1954;34(3):529–562. 13185754 10.1152/physrev.1954.34.3.529

[46] Brokaw CJ. Chemotaxis of bracken spermatozoids: implications of electrochemical orientation. J Exp Biol. 1958;35(1):197–212.

[47] Brokaw CJ. Chemotaxis of bracken spermatozoids: the role of bimalate ions. J Exp Biol. 1958;35(1):192–196.

[48] Bronner M. Riding the crest for 150 years! Dev Biol. 2018;444:S1–S2. 30904167 10.1016/j.ydbio.2019.03.001

[49] Harrison RG, Greenman MJ, Mall FP, et al. Observations of the living developing nerve fiber. Anat Rec. 1907;1(5):116–128.

[50] Ramon Y Cajal S. La rétine des vertébrés. Cellule. 1893;9:119–255.

[51] His W. Untersuchungen über die erste Anlage des Wirbelthierleibes: die erste Entwickelung des Hühnchens im Ei. Vol. 1. FCW Vogel; 1868.

[52] Jain N, Moeller J, Vogel V. Mechanobiology of macrophages: how physical factors coregulate macrophage plasticity and phagocytosis. Annu Rev Biomed Eng. 2019;21:267–297. 31167103 10.1146/annurev-bioeng-062117-121224

[53] Boyden S. The chemotactic effect of mixtures of antibody and antigen on polymorphonuclear leucocytes. J Exp Med. 1962;115(3):453–466. 13872176 10.1084/jem.115.3.453 PMC2137509

[54] McCutcheon M. Chemotaxis in leukocytes. Physiol Rev. 1946;26(3):319–336. 20993553 10.1152/physrev.1946.26.3.319

[55] Donà E, Barry JD, Valentin G, et al. Directional tissue migration through a self-generated chemokine gradient. Nature. 2013;503(7475):285–289. 24067609 10.1038/nature12635

[56] Tweedy L, Thomason PA, Paschke PI, et al. Seeing around corners: cells solve mazes and respond at a distance using attractant breakdown. Science (80). 2020;369:6507. 10.1126/science.aay9792 32855311

[57] Plou J, Juste-Lanas Y, Olivares V, et al. From individual to collective 3D cancer dissemination: roles of collagen concentration and TGF-*β*. Scientific reports *Reports 2018 8* 2018;8(1):1–14. 30143683 10.1038/s41598-018-30683-4 PMC6109049

[58] Ungefroren H, Witte D, Lehnert H. The role of small GTPases of the Rho/Rac family in TGF-*β*-induced EMT and cell motility in cancer. Dev Dyn. 2018;247(3):451–461. 28390160 10.1002/dvdy.24505

[59] López-Luque J, Bertran E, Crosas-Molist E, et al. Downregulation of Epidermal growth factor receptor in hepatocellular carcinoma facilitates transforming growth Factor-*β*-induced epithelial to amoeboid transition. Cancer Lett. 2019;464:15–24. 31465839 10.1016/j.canlet.2019.08.011 PMC6853171

[60] Wu JS, Jiang J, Jun Chen B, et al. Plasticity of cancer cell invasion: patterns and mechanisms. Transl Oncol. 2021;14(1):100899. 33080522 10.1016/j.tranon.2020.100899 PMC7573380

[61] Wenqian L, Zhang X, Wang J, et al. TGF-*β*1 in fibroblasts-derived exosomes promotes epithelial-mesenchymal transition of ovarian cancer cells. Oncotarget. 2017;8(56):96035–96047. 29221185 10.18632/oncotarget.21635 PMC5707079

[62] Gao J, Zhu Y, Nilsson M, et al. TGF-*β* isoforms induce EMT independent migration of ovarian cancer cells. Cancer Cell Int. 2014;14(1):1–10. 25278811 10.1186/s12935-014-0072-1 PMC4180856

[63] Kumar Jolly M, Somarelli JA, Sheth M, et al. Hybrid epithelial/mesenchymal phenotypes promote metastasis and therapy resistance across carcinomas. Pharmacol Ther. 2019;194:161–184. 30268772 10.1016/j.pharmthera.2018.09.007

[64] Pastushenko I, Blanpain C. EMT transition states during tumor progression and metastasis. Trends Cell Biol. 2019;29(3):212–226. 30594349 10.1016/j.tcb.2018.12.001

[65] Miaczynska M. Effects of membrane trafficking on signaling by receptor tyrosine kinases. Cold Spring Harb Perspect Biol. 2013;5(11):a009035. 24186066 10.1101/cshperspect.a009035 PMC3809584

[66] Jessica BC, Andrea IM. Spatial regulation of receptor tyrosine kinases in development and cancer. Nat Rev Cancer. 2012;12(6):387–400. 22622641 10.1038/nrc3277 PMC3767127

[67] Faron-Górecka A, Szlachta M, Kolasa M, et al. Understanding GPCR dimerization. Methods Cell Biol. 2019;149:155–178. 30616817 10.1016/bs.mcb.2018.08.005

[68] Michael DP, Hristova K. The RTK Interactome: overview and Perspective on RTK Heterointeractions. Chem Rev. 2019;119(9):5881–5921. 30589534 10.1021/acs.chemrev.8b00467 PMC6918702

[69] Henrik Heldin C, Benson L, Evans R, et al. Signals and receptors. Cold Spring Harb Perspect Biol. 2016;8(4):a005900. 27037414 10.1101/cshperspect.a005900 PMC4817805

[70] MacDonald E, Savage B, Zech T. Connecting the dots: combined control of endocytic recycling and degradation. Biochem Soc Trans. 2020;48(6):2377–2386. 33300959 10.1042/BST20180255 PMC7752043

[71] Nathan JP, Peter AF. GPCR signaling and trafficking: the long and short of it. Trends Endocrinol Metab. 2017;28(3):213–226. 27889227 10.1016/j.tem.2016.10.007 PMC5326587

[72] Stefanie LR, Randy AH. Fine-tuning of GPCR activity by receptor-interacting proteins. Nat Rev Mol Cell Biol. 2009;10(12):819–830. 19935667 10.1038/nrm2803 PMC2825052

[73] Simon RF, Bräuner-Osborne H. Investigating internalization and intracellular trafficking of gpcrs: new techniques and real-time experimental approaches. Handb Exp Pharmacol. 2017;245:41–61. 10.1007/164_2017_57 29018878

[74] Irannejad R, Von Zastrow M. GPCR signaling along the endocytic pathway. Curr Opin Cell Biol. 2014;27(1):109–116. 24680436 10.1016/j.ceb.2013.10.003 PMC4968408

[75] Ulloa-Aguirre A, Ann Janovick J, Zariñán T, et al. Intracellular trafficking of G protein-coupled receptors to the cell surface plasma membrane in health and disease. Cell Endocrinol Health Dis. 2021;375–412.

[76] Du Z, Christine ML, Li Y. Mechanisms of receptor tyrosine kinase activation in cancer. Mol Cancer. 2018;17(1):1–13. 29455648 10.1186/s12943-018-0782-4 PMC5817791

[77] Trenker R, Jura N. Receptor tyrosine kinase activation: from the ligand perspective. Curr Opin Cell Biol. 2020;63:174–185. 32114309 10.1016/j.ceb.2020.01.016 PMC7813211

[78] Laura EK, Stephen JH. Transactivation of G protein-coupled receptors (GPCRs) and receptor tyrosine kinases (RTKs): recent insights using luminescence and fluorescence technologies. Curr Opin Endocr Metab Res. 2021;16:102–112. 33748531 10.1016/j.coemr.2020.10.003 PMC7960640

[79] Sunyer R, Conte V, Escribano J, et al. Collective cell durotaxis emerges from long-range intercellular force transmission. Science. 2016;353(6304):1157–1161. 27609894 10.1126/science.aaf7119

[80] Ladoux B, Marc Mège R. Mechanobiology of collective cell behaviours. Nat Rev Mol Cell Biol. 2017;18(12):743–757. 29115298 10.1038/nrm.2017.98

[81] Chen S, Hourwitz MJ, Campanello L, et al. Actin Cytoskeleton and focal adhesions regulate the biased migration of breast cancer cells on nanoscale asymmetric sawteeth. ACS Nano. 2019;13(2):1454–1468. 30707556 10.1021/acsnano.8b07140 PMC7159974

[82] Lise Le Roux A, Quiroga X, Walani N, et al. The plasma membrane as a mechanochemical transducer. Philos Trans R Soc B Biol Sci. 2019;374:1779. 10.1098/rstb.2018.0221 PMC6627014 31431176

[83] Sunyer R, Trepat X. Durotaxis. Curr Biol. 2020;30(9):R383–R387. 32369745 10.1016/j.cub.2020.03.051

[84] DuChez BJ, Doyle AD, Dimitriadis EK, et al. Durotaxis by human cancer cells. Biophys J. 2019;116(4):670–683. 30709621 10.1016/j.bpj.2019.01.009 PMC6382956

[85] Barriga EH, Franze K, Charras G, et al. Tissue stiffening coordinates morphogenesis by triggering collective cell migration in vivo. Nature. 2018;554(7693):523–527. 29443958 10.1038/nature25742 PMC6013044

[86] Isomursu A, Park K-Y, Hou J, et al. Negative durotaxis: cell movement toward softer environments. bioRxiv. 2020. https://www.biorxiv.org/content/10.1101/2020.10.27.357178v1

[87] Sarah TB, Zahied Johan M, Michael SS. Tumour-directed microenvironment remodelling at a glance. J Cell Sci. 2020;133(24):jcs247783. 33443095 10.1242/jcs.247783

[88] Beunk L, Brown K, Nagtegaal I, et al. Cancer invasion into musculature: mechanics, molecules and implications. Semin Cell Dev Biol. 2019;93:36–45. 30009945 10.1016/j.semcdb.2018.07.014 PMC6399078

[89] Boon Kai F, Drain AP, Weaver VM. The extracellular matrix modulates the metastatic journey. Dev Cell. 2019;49(3):332–346. 31063753 10.1016/j.devcel.2019.03.026 PMC6527347

[90] Malandrino A, Mak M, Kamm RD, et al. Complex mechanics of the heterogeneous extracellular matrix in cancer. Extrem Mech Lett. 2018;21:25–34. 10.1016/j.eml.2018.02.003 PMC6097546 30135864

[91] Berger AJ, Renner CM, Hale I, et al. Scaffold stiffness influences breast cancer cell invasion via EGFR-linked Mena upregulation and matrix remodeling. Matrix Biol. 2020;85-86:80–93. 31323325 10.1016/j.matbio.2019.07.006 PMC6962577

[92] Hayn A, Fischer T, Tanja Mierke C. Inhomogeneities in 3D collagen matrices impact matrix mechanics and cancer cell migration. Front Cell Dev Biol. 2020;8:1224. 10.3389/fcell.2020.593879 PMC7674772 33251219

[93] Taufalele PV, VanderBurgh JA, Muñoz A, et al. Fiber alignment drives changes in architectural and mechanical features in collagen matrices. PLoS One. 2019;14(5):e0216537. 31091287 10.1371/journal.pone.0216537 PMC6519824

[94] Sapudom J, Kalbitzer L, Xiancheng W, et al. Fibril bending stiffness of 3D collagen matrices instructs spreading and clustering of invasive and non-invasive breast cancer cells. Biomaterials. 2019;193:47–57. 30554026 10.1016/j.biomaterials.2018.12.010

[95] Anguiano M, Morales X, Castilla C, et al. The use of mixed collagen-Matrigel matrices of increasing complexity recapitulates the biphasic role of cell adhesion in cancer cell migration: ECM sensing, remodeling and forces at the leading edge of cancer invasion. PLoS One. 2020;15(1):e0220019. 31945053 10.1371/journal.pone.0220019 PMC6964905

[96] Balcioglu HE, Balasubramaniam L, Vasilica Stirbat TV, et al. A subtle relationship between substrate stiffness and collective migration of cell clusters. Soft Matter. 2020;16(7):1825–1839. 31970382 10.1039/c9sm01893j

[97] Janmey PA, Fletcher DA, Reinhart-King CA. Stiffness Sensing by Cells. Physiol Rev. 2020;100(2):695–724. 31751165 10.1152/physrev.00013.2019 PMC7276923

[98] Bangasser BL, Shamsan GA, Chan CE, et al. Shifting the optimal stiffness for cell migration. Nat Commun. 2017;8(1):15313. 28530245 10.1038/ncomms15313 PMC5458120

[99] Gkretsi V, Stylianopoulos T. Cell adhesion and matrix stiffness: coordinating cancer cell invasion and metastasis. Front Oncol. 2018;8:145. 29780748 10.3389/fonc.2018.00145 PMC5945811

[100] Chang J, Pang EM, Adebowale K, et al. Increased stiffness inhibits invadopodia formation and cell migration in 3D. Biophys J. 2020;119(4):726–736. 32697977 10.1016/j.bpj.2020.07.003 PMC7451915

[101] Cóndor M, Mark C, Gerum RC, et al. Breast cancer cells adapt contractile forces to overcome steric hindrance. Biophys J. 2019;116(7):1305–1312. 30902366 10.1016/j.bpj.2019.02.029 PMC6451061

[102] Higgins G, Kim JE, Ferruzzi J, et al. Decreased cell stiffness facilitates cell detachment and cell migration from breast cancer spheroids in 3D collagen matrices of different rigidity. bioRxiv. 2021. https://www.biorxiv.org/content/10.1101/2021.01.21.427639v4

[103] Panzetta V, Fusco S, Netti PA. Cell mechanosensing is regulated by substrate strain energy rather than stiffness. Proc Natl Acad Sci U S A. 2019;116(44):22004–22013. 31570575 10.1073/pnas.1904660116 PMC6825315

[104] Jing L, Jung W, Nam S, et al. Roles of interactions between cells and extracellular matrices for cell migration and matrix remodeling. In Zhang Yanhang editor. Multi-scale extracell. matrix mech. mechanobiol. Springer; 2020. p. 247–282. https://link.springer.com/chapter/10.1007/978-3-030-20182-1_8

[105] Kim J, Cao Y, Eddy C, et al. The mechanics and dynamics of cancer cells sensing noisy 3D contact guidance. Proc Natl Acad Sci U S A. 118(10):2021. 10.1073/pnas.2024780118 PMC7958230 33658384

[106] Fraley SI, Hsun Wu P-H, Lijuan L, et al. Three-dimensional matrix fiber alignment modulates cell migration and MT1-MMP utility by spatially and temporally directing protrusions. Sci Rep. 2015;5(1):14580. 26423227 10.1038/srep14580 PMC4589685

[107] Ray A, Slama ZM, Morford RK, et al. Enhanced directional migration of cancer stem cells in 3D aligned collagen matrices. Biophys J. 2017;112(5):1023–1036. 28297639 10.1016/j.bpj.2017.01.007 PMC5355487

[108] Lautscham LA, Kämmerer C, Lange JR, et al. Migration in confined 3D environments is determined by a combination of adhesiveness, nuclear volume, contractility, and cell stiffness. Biophys J. 2015;109(5):900–913. 26331248 10.1016/j.bpj.2015.07.025 PMC4564685

[109] Friedl P, Wolf K. Plasticity of cell migration: a multiscale tuning model. J Cell Biol. 2010;188(1):11–19. 19951899 10.1083/jcb.200909003 PMC2812848

[110] Velez DO, Ranamukhaarachchi SK, Kumar A, et al. 3D collagen architecture regulates cell adhesion through degradability, thereby controlling metabolic and oxidative stress. Integr Biol. 2019;11(5):221–234. 10.1093/intbio/zyz019 PMC6657753 31251330

[111] Leiphart RJ, Chen D, Peredo AP, et al. Mechanosensing at Cellular Interfaces. Langmuir. 2019;35(23):7509–7519. 30346180 10.1021/acs.langmuir.8b02841

[112] James RWC, Jacquemet G, Malliri A. Cell matrix adhesion in cell migration. Essays Biochem. 2019;63(5):535–551. 31444228 10.1042/EBC20190012

[113] Seetharaman S, Etienne-Manneville S. Integrin diversity brings specificity in mechanotransduction. Biol Cell. 2018;110(3):49–64. 29388220 10.1111/boc.201700060

[114] Kim S, Uroz M, Bays JL, et al. Harnessing mechanobiology for tissue engineering. Dev Cell. 2021;56(2):180–191. 33453155 10.1016/j.devcel.2020.12.017 PMC7855912

[115] Fattet L, Yun Jung H-Y, Matsumoto MW, et al. Matrix rigidity controls epithelial-mesenchymal plasticity and tumor metastasis via a Mechanoresponsive EPHA2/LYN complex. Dev Cell. 2020;54(3):302–316.e7. 32574556 10.1016/j.devcel.2020.05.031 PMC7423770

[116] Burridge K, Monaghan-Benson E, Graham DM. Mechanotransduction: from the cell surface to the nucleus via RhoA. Philos Trans R Soc B Biol Sci. 2019;374(1779):20180229. 10.1098/rstb.2018.0229 PMC6627015 31431179

[117] Humphries JD, Chastney MR, Askari JA, et al. Signal transduction via integrin adhesion complexes. Curr Opin Cell Biol. 2019;56:14–21. 30195153 10.1016/j.ceb.2018.08.004

[118] Samaržija I, Dekanić A, Humphries JD, et al. Integrin crosstalk contributes to the complexity of signalling and unpredictable cancer cell fates. Cancers (Basel). 2020;12(7):1910. 10.3390/cancers12071910 PMC7409212 32679769

[119] Bachmann M, Kukkurainen S, Hytönen VP, et al. Cell adhesion by integrins. Physiol Rev. 2019;99(4):1655–1699. 31313981 10.1152/physrev.00036.2018

[120] Kechagia JZ, Ivaska J, Roca-Cusachs P. Integrins as biomechanical sensors of the microenvironment. Nat Rev Mol Cell Biol. 2019;20(8):457–473. 31182865 10.1038/s41580-019-0134-2

[121] SenGupta S, Parent CA, Bear JE. The principles of directed cell migration. Nat Rev Mol Cell Biol. 2021;22:1–19. 33990789 10.1038/s41580-021-00366-6 PMC8663916

[122] Chastney MR, Conway JRW, Ivaska J. Integrin adhesion complexes. Curr Biol. 2021;31(10):R536–R542. 34033786 10.1016/j.cub.2021.01.038

[123] Peláez R, Larrayoz IM, Pérez-Sala Á, et al. Integrins: moonlighting proteins in invadosome formation. Cancers (Basel). 2019;11(5):615. 10.3390/cancers11050615 PMC6562994 31052560

[124] Hamidi H, Ivaska J. Every step of the way: integrins in cancer progression and metastasis. Nat Rev Cancer. 2018;18(9):533–548. 30002479 10.1038/s41568-018-0038-z PMC6629548

[125] Sun Z, Costell M, Fässler R. Integrin activation by talin, kindlin and mechanical forces. Nat Cell Bio. 21(1):2019. 10.1038/s41556-018-0234-9 30602766

[126] Karimi F, O’Connor AJ, Qiao GG, et al. Integrin clustering matters: a review of biomaterials functionalized with multivalent integrin-binding ligands to improve cell adhesion, migration, differentiation, angiogenesis, and biomedical device integration. Adv Healthc Mater. 2018;7(12):1701324. 10.1002/adhm.201701324 29577678

[127] Farage E, Patrick TC. Quantitative analysis of integrin trafficking. In: Methods mol. biol. Vol. 2217. Humana Press Inc; 2021. p. 251–263. 33215385 10.1007/978-1-0716-0962-0_14

[128] Moreno-Layseca P, Icha J, Hamidi H, et al. Integrin trafficking in cells and tissues. Nat Cell Bio. 2019;21(2):122–132. 30602723 10.1038/s41556-018-0223-z PMC6597357

[129] Alfonzo-Mendez MA, Sochacki KA, Paule Strub M, et al. Dual clathrin and adhesion signaling systems regulate growth factor receptor activation. bioRxiv. 2020;12:1910. 10.1038/s41467-022-28373-x PMC8850434 35173166

[130] Baade T, Paone C, Baldrich A, et al. Clustering of integrin *β* cytoplasmic domains triggers nascent adhesion formation and reveals a protozoan origin of the integrin-talin interaction. Sci Rep. 2019;9(1):1–13. 30952878 10.1038/s41598-019-42002-6 PMC6450878

[131] Oria R, Wiegand T, Escribano J, et al. Force loading explains spatial sensing of ligands by cells. Nature. 2017;552(7684):219–224. 29211717 10.1038/nature24662

[132] Changede R, Cai H, Wind SJ, et al. Integrin nanoclusters can bridge thin matrix fibres to form cell–matrix adhesions. Nat Mater. 2019;18(12):1366–1375. 31477904 10.1038/s41563-019-0460-y PMC7455205

[133] Martino F, Perestrelo AR, Vinarský V, et al. Cellular mechanotransduction: from tension to function. Front Physiol. 2018;9:824. 30026699 10.3389/fphys.2018.00824 PMC6041413

[134] Kleinschmidt EG, Schlaepfer DD. Focal adhesion kinase signaling in unexpected places. Curr Opin Cell Biol. 2017;45:24–30. 28213315 10.1016/j.ceb.2017.01.003 PMC5482783

[135] Noh K, Hiep Bach D, Jin Choi H, et al. The hidden role of paxillin: localization to nucleus promotes tumor angiogenesis. Oncogene. 2020;40(2):384–395. 33149280 10.1038/s41388-020-01517-3 PMC8275353

[136] Rabanal-Ruiz Y, Byron A, Wirth A, et al. mTORC1 activity is supported by spatial association with focal adhesions. J Cell Biol. 220(5):2021. 10.1083/jcb.202004010 PMC7923692 33635313

[137] Yusheng W, Zanotelli MR, Zhang J, et al. Matrix-driven changes in metabolism support cytoskeletal activity to promote cell migration. Biophys J. 2021. 10.1016/j.bpj.2021.02.044 PMC8204337 33705759

[138] Suk Park J, Burckhardt CJ, Lazcano R, et al. Mechanical regulation of glycolysis via cytoskeleton architecture. Nature. 2020;578(7796):621–626. 32051585 10.1038/s41586-020-1998-1 PMC7210009

[139] Campbell DL, Anne JR. Rho GTPase signaling complexes in cell migration and invasion. J Cell Biol. 2018;217(2):447–457. 29233866 10.1083/jcb.201612069 PMC5800797

[140] Jansen S, Gosens R, Wieland T, et al. Paving the Rho in cancer metastasis: rho GTPases and beyond. Pharmacol Ther. 2018;183:1–21. 28911825 10.1016/j.pharmthera.2017.09.002

[141] Natasha SC, Anne JR. Targeting Rho GTPase signaling networks in cancer. Front Cell Dev Biol. 2020;8:222. 32309283 10.3389/fcell.2020.00222 PMC7145979

[142] Cerutti C, Anne JR. Endothelial cell-cell adhesion and signaling. Exp Cell Res. 2017;358(1):31–38. 28602626 10.1016/j.yexcr.2017.06.003 PMC5700119

[143] Haga RB, Garg R, Collu F, et al. RhoBTB1 interacts with ROCKs and inhibits invasion. Biochem J. 2019;476(17):2499–2514. 31431478 10.1042/BCJ20190203 PMC6744581

[144] Julius HS, Brakebusch C. Rho GTPases in cancer: friend or foe? Oncogene. 2019;38(50):7447–7456. 31427738 10.1038/s41388-019-0963-7

[145] Aspenström P. Activated Rho GTPases in cancer—the beginning of a new paradigm. Int J Mol Sci. 2018;19(12):3949. 10.3390/ijms19123949 PMC6321241 30544828

[146] Xosé RB. RHO GTPases in cancer: known facts, open questions, and therapeutic challenges. Biochem Soc Trans. 2018;46(3):741–760. 29871878 10.1042/BST20170531 PMC7615761

[147] Chinthalapudi K, Rangarajan ES, Izard T. The interaction of talin with the cell membrane is essential for integrin activation and focal adhesion formation. Proceedings of the National Academy of Sciences. 2018;115(41):10339–10344. 10.1073/pnas.1806275115 PMC6187153 30254158

[148] Van Helvert S, Storm C, Friedl P. Mechanoreciprocity in cell migration. Nat Cell Bio. 2018;20(1):8–20. 29269951 10.1038/s41556-017-0012-0 PMC5943039

[149] Kluger C, Braun L, Sedlak SM, et al. Different vinculin binding sites use the same mechanism to regulate directional force transduction. Biophys J. 2020;118(6):1344–1356. 32109366 10.1016/j.bpj.2019.12.042 PMC7091509

[150] Andreu I, Falcones B, Hurst S, et al. The force loading rate drives cell mechanosensing through both reinforcement and cytoskeletal softening. Nat Commun. 2021;12(1):4229. 34244477 10.1038/s41467-021-24383-3 PMC8270983

[151] Newman D, Young L, Waring T, et al. 3D matrix adhesion composition facilitates nuclear force coupling to drive invasive cell migration. bioRxiv. 2021. 10.1016/j.celrep.2023.113554 38100355

[152] Theveneau E, Linker C. Leaders in collective migration: are front cells really endowed with a particular set of skills? F1000Res. 2017;6:1899. 29152225 10.12688/f1000research.11889.1 PMC5664975

[153] De Pascalis C, Pérez-González C, Seetharaman S, et al. Intermediate filaments control collective migration by restricting traction forces and sustaining cell-cell contacts. J Cell Biol. 2018;217(9):3031–3044. 29980627 10.1083/jcb.201801162 PMC6122997

[154] Arnold TR, Stephenson RE, Miller AL. Rho GTPases and actomyosin: partners in regulating epithelial cell-cell junction structure and function. Exp Cell Res. 2017;358(1):20–30. 28363828 10.1016/j.yexcr.2017.03.053 PMC5544599

[155] Singh J, Pagulayan A, Camley BA, et al. Rules of contact inhibition of locomotion for cells on suspended nanofibers. Proceedings of the National Academy of Sciences. 2021;118(12):e2011815118. 10.1073/pnas.2011815118 PMC8000107 33737392

[156] Roycroft A, Mayor R. Michael abercrombie: contact inhibition of locomotion and more. Int J Dev Biol. 2018;62(1–3):5–13. 29616739 10.1387/ijdb.170277rm

[157] Abercrombie M. Contact inhibition in tissue culture. Vitro. 1970;6(2):128–142. 10.1007/BF02616114 4943054

[158] Loeb L. AMOEBOID MOVEMENT,TISSUE FORMATION AND CONSISTENCY OF PROTOPLA-SM. Am J Physiol Content. 1921;56(1):140–167.

[159] Thomas LA, Yamada KM. Contact stimulation of cell migration. J Cell Sci. 1992;103(4):1211–1214. 1487497 10.1242/jcs.103.4.1211

[160] Miekus K, Czernik M, Sroka J, et al. Contact stimulation of prostate cancer cell migration: the role of gap junctional coupling and migration stimulated by heterotypic cell-to-cell contacts in determination of the metastatic phenotype of Dunning rat prostate cancer cells. Biol Cell. 2005;97(12):893–903. 15907197 10.1042/BC20040129

[161] Bischoff MC, Lieb S, Renkawitz-Pohl R, et al. Filopodia-based contact stimulation of cell migration drives tissue morphogenesis. Nat Commun. 2021;12(1):1–18. 33542237 10.1038/s41467-020-20362-2 PMC7862658

[162] Balasubramaniam L, Doostmohammadi A, Beng Saw T, et al. Investigating the nature of active forces in tissues reveals how contractile cells can form extensile monolayers. Nat Mater. 2021;1–11. 33603188 10.1038/s41563-021-00919-2 PMC7611436

[163] Nguyen T, Duchesne L, Hari Narayana Sankara Narayana G, et al. Enhanced cell–cell contact stability and decreased N-cadherin-mediated migration upon fibroblast growth factor receptor-N-cadherin cross talk. Oncogene. 2019;38(35):6283–6300. 31312021 10.1038/s41388-019-0875-6

[164] Hohmann T, Dehghani F. The cytoskeleton—A complex interacting meshwork. Cells. 2019;8(4):362. 10.3390/cells8040362 PMC6523135 31003495

[165] Bershadsky AD, Vasiliev JM. Cytoskeleton. 1st ed. Springer Science & Business Media; 1988.

[166] Koltzoff N. Experimental biology and the work of the Moscow institute. Science. 1924;59(1536):497–502. 17775910 10.1126/science.59.1536.497

[167] Seetharaman S, Etienne-Manneville S. Cytoskeletal crosstalk in cell migration. Trends Cell Biol. 2020;30(9):720–735. 32674938 10.1016/j.tcb.2020.06.004

[168] Pegoraro AF, Janmey P, Weitz DA. Mechanical properties of the cytoskeleton and cells. Cold Spring Harb Perspect Biol. 2017;9(11):a022038. 29092896 10.1101/cshperspect.a022038 PMC5666633

[169] Kadzik RS, Homa KE, Kovar DR. F-Actin cytoskeleton network self-organization through competition and cooperation. Annu Rev Cell Dev Biol. 2020;36:35–60. 33021819 10.1146/annurev-cellbio-032320-094706 PMC8675537

[170] Senju Y, Lappalainen P. Regulation of actin dynamics by PI(4,5)P2 in cell migration and endocytosis. Curr Opin Cell Biol. 2019;56:7–13. 30193157 10.1016/j.ceb.2018.08.003

[171] Warner H, Wilson BJ, Caswell PT. Control of adhesion and protrusion in cell migration by Rho GTPases. Curr Opin Cell Biol. 2019;56:64–70. 30292078 10.1016/j.ceb.2018.09.003 PMC6368645

[172] Lehtimaki J, Hakala M, Lappalainen P. Actin filament structures in migrating cells. Handb Exp Pharmacol. 2017;235:1–30. 27469496 10.1007/164_2016_28

[173] Raquel RB, Anne JR. Rho GTPases: regulation and roles in cancer cell biology. Small GTPases. 2016;7(4):207–221. 27628050 10.1080/21541248.2016.1232583 PMC5129894

[174] Krajnik A, Brazzo JA, Vaidyanathan K, et al. Phosphoinositide signaling and mechanotransduction in cardiovascular biology and disease. Front Cell Dev Biol. 2020;8:1588. 10.3389/fcell.2020.595849 PMC7767973 33381504

[175] Sun Y, Yue H, Copos C, et al. PI3K inhibition reverses migratory direction of single cells but not cell groups in electric field. bioRxiv. 2020. https://www.biorxiv.org/content/10.1101/2020.08.05.238170v1

[176] Insall R. Actin in 2021. Curr Biol. 2021;31(10):R496–R498. 34033777 10.1016/j.cub.2021.04.013

[177] Ruggiero C, Lalli E. Targeting the cytoskeleton against metastatic dissemination. Cancer Metastasis Rev. 2021;40:1–52. 33471283 10.1007/s10555-020-09936-0

[178] Buracco S, Claydon S, Insall R. Control of actin dynamics during cell motility. F1000Res. 2019;8:1977. 10.12688/f1000research.18669.1 PMC6880267 31824651

[179] Sitarska E, Diz-Muñoz A. Pay attention to membrane tension: mechanobiology of the cell surface. Current Opinion in Cell Biology. 2020;66:11–18. 32416466 10.1016/j.ceb.2020.04.001 PMC7594640

[180] Welf ES, Miles CE, Huh J, et al. Actin-membrane release initiates cell protrusions. Developmental Cell. 2020;55(6):723–736.e8. 33308479 10.1016/j.devcel.2020.11.024 PMC7908823

[181] Bisaria A, Hayer A, Garbett D, et al. Membrane-proximal F-actin restricts local membrane protrusions and directs cell migration. Science. 2020;368(6496):1205–1210. 32527825 10.1126/science.aay7794 PMC8283920

[182] Ullo MF, Logue JS. ADF and cofilin-1 collaborate to promote cortical actin flow and the leader bleb-based migration of confined cells. Elife. 2021;10:e67856. DOI:10.7554/eLife.67856. PMC8253594 34169836

[183] Kanellos G, Zhou J, Patel H, et al. ADF and cofilin1 control actin stress fibers, nuclear integrity, and cell survival. Cell Rep. 2015;13(9):1949–1964. 26655907 10.1016/j.celrep.2015.10.056 PMC4678118

[184] Prakash Singh SP, Insall H. Insall. Adhesion stimulates Scar/WAVE phosphorylation in mammalian cells. Communicative & Integrative Biology. 2021;14(1):1–4. 10.1080/19420889.2020.1855854 PMC7781634 33447346

[185] Prakash Singh SP, Thomason PA, Lilla S, et al. Cell–substrate adhesion drives Scar/WAVE activation and phosphorylation by a Ste20-family kinase, which controls pseudopod lifetime. PLOS Biology. 2020;18(8):e3000774. 32745097 10.1371/journal.pbio.3000774 PMC7425996

[186] Carey SP, Goldblatt ZE, Martin KE, et al. Local extracellular matrix alignment directs cellular protrusion dynamics and migration through Rac1 and FAK. Integrative Biology. 2016;8(8):821–835. 27384462 10.1039/c6ib00030d PMC4980151

[187] Adebowale K, Gong Z, Hou JC, et al. Enhanced substrate stress relaxation promotes filopodia-mediated cell migration. Nat Mater. 2021;20(9):1–10. https://www.nature.com/articles/s41563-021-00981-w#citeas 33875851 10.1038/s41563-021-00981-w PMC8390443

[188] Kelkar M, Bohec P, Charras G. Mechanics of the cellular actin cortex: from signalling to shape change. Current Opinion in Cell Biology. 2020;66:69–78. 32580115 10.1016/j.ceb.2020.05.008

[189] Caswell PT. letting go to move on: membrane detachment initiates protrusion. Developmental Cell. 2020;55(6):671–672. 33352139 10.1016/j.devcel.2020.11.026

[190] Bte Mohd Rafiq N, Nishimura Y, Plotnikov SV, et al. A mechano-signalling network linking microtubules, myosin IIA filaments and integrin-based adhesions. Nature Materials. 2019;18(6):638–649. 31114072 10.1038/s41563-019-0371-y

[191] Lin J, Shi Y, Men Y, et al. Mechanical roles in formation of oriented collagen fibers. Tissue Engineering Part B: Reviews. 2020;26(2):116–128. 31801418 10.1089/ten.TEB.2019.0243

[192] Olivares V, Cóndor M, Del Amo C, et al. Image-based characterization of 3D collagen networks and the effect of embedded cells. Microscopy and Microanalysis. 2019;25(4):971–981. 31210124 10.1017/S1431927619014570

[193] Kim J, Feng J, Jones CAR, et al. Stress-induced plasticity of dynamic collagen networks. Nature Communications. 2017;8(1):842. 10.1038/s41467-017-01011-7 PMC5635002 29018207

[194] Elosegui-Artola A, Trepat X, Roca-Cusachs P. Control of mechanotransduction by molecular clutch dynamics. Trends Cell Biol. 2018;28(5):356–367. 29496292 10.1016/j.tcb.2018.01.008

[195] Yeoman B, Shatkin G, Beri P, et al. Adhesion strength and contractility enable metastatic cells to become adurotactic. Cell Rep. 2021;34(10):108816. 33691109 10.1016/j.celrep.2021.108816 PMC7997775

[196] Chizhik AM, Wollnik C, Ruhlandt D, et al. Dual-color metal-induced and Förster resonance energy transfer for cell nanoscopy. Molecular Biology of the Cell. 2018;29(7):846–851. 29444956 10.1091/mbc.E17-05-0314 PMC5905297

[197] Lee S, Kumar S. Actomyosin stress fiber mechanosensing in 2D and 3D. F1000Res. 2016;5:2261. version 1; referees: 3 approved. 10.12688/f1000research.8800.1 PMC5017290 27635242

[198] Livne A, Geiger B. The inner workings of stress fibers − from contractile machinery to focal adhesions and back. Journal of Cell Science. 2016;129(7):1293–1304. 27037413 10.1242/jcs.180927

[199] Lee S, Kassianidou E, Kumar S. Actomyosin stress fiber subtypes have unique viscoelastic properties and roles in tension generation. Molecular Biology of the Cell. 2018;29(16):1992–2004. 29927349 10.1091/mbc.E18-02-0106 PMC6232976

[200] Vignaud T, Copos C, Leterrier C, et al. Stress fibres are embedded in a contractile cortical network. Nat Mater. 2020;20(3): 1–11. https://www.nature.com/articles/s41563-020-00825-z#citeas 10.1038/s41563-020-00825-z PMC7610471 33077951

[201] Tavares S, Filipe Vieira AF, Verena Taubenberger AV, et al. Actin stress fiber organization promotes cell stiffening and proliferation of pre-invasive breast cancer cells. Nature Communications. 2017;8(1):1–18. 10.1038/ncomms15237 PMC5440822 28508872

[202] Caswell PT, Zech T. Actin-based cell protrusion in a 3D matrix. Trends Cell Biol. 2018;28(10):823–834. 29970282 10.1016/j.tcb.2018.06.003 PMC6158345

[203] Stylianou A, Gkretsi V, Louca M, et al. Collagen content and extracellular matrix cause cytoskeletal remodelling in pancreatic fibroblasts. J R Soc Interface. 16(154):2019. 10.1098/rsif.2019.0226 PMC6544883 31113335

[204] Kunitski M, Eicke N, Huber P, et al. Double-slit photoelectron interference in strong-field ionization of the neon dimer. Nature Communications. 2019;10(1):1–9. DOI:10.1038/s41467-018-07882-8 PMC6315036 30602773

[205] Dogterom M, Koenderink GH. Actin–microtubule crosstalk in cell biology. Nature Reviews Molecular Cell Biology. 2019;20(1):38–54. 30323238 10.1038/s41580-018-0067-1

[206] Garcin C, Straube A, Malliri A. Microtubules in cell migration. Essays Biochem. 2019;63(5):509–520. 31358621 10.1042/EBC20190016 PMC6823166

[207] Torrino S, Grasset EM, Audebert S, et al. Mechano-induced cell metabolism promotes microtubule glutamylation to force metastasis. Cell Metab. 2021;33(7):1342–1357.e10. 34102109 10.1016/j.cmet.2021.05.009

[208] Seetharaman S, Etienne-Manneville S. Microtubules at focal adhesions - a double-edged sword. J Cell Sci. 132(19): 2019.https://journals.biologists.com/jcs/article/132/19/jcs232843/223908/Microtubules-at-focal-adhesions-a-double-edged 10.1242/jcs.232843 31597743

[209] Bouchet BP, Akhmanova A, Ewald A. Microtubules in 3D cell motility. Journal of Cell Science. 2017;130(1):39–50. 28043967 10.1242/jcs.189431

[210] Zinn A, Goicoechea SM, Kreider-Letterman G, et al. The small GTPase RhoG regulates microtubule-mediated focal adhesion disassembly. Scientific Reports. 2019;9(1):1–15. 30914742 10.1038/s41598-019-41558-7 PMC6435757

[211] Bouchet BP, Noordstra I, van Amersfoort M, et al. Mesenchymal cell invasion requires cooperative regulation of persistent microtubule growth by SLAIN2 and CLASP1. Developmental Cell. 2016;39(6):708–723. 27939686 10.1016/j.devcel.2016.11.009 PMC5178967

[212] Raab M, Discher DE. Matrix rigidity regulates microtubule network polarization in migration. Cytoskeleton. 2017;74(3):114–124. 27935261 10.1002/cm.21349 PMC5352467

[213] Pimm ML, Henty-Ridilla JL, Bement W. New twists in actin–microtubule interactions. Molecular Biology of the Cell. 2021;32(3):211–217. 33507109 10.1091/mbc.E19-09-0491 PMC8098829

[214] Seetharaman S, Vianay B, Roca V, Farrugia, A. J., De Pascalis, C., Boëda, B., Dingli, F., Loew, D., Vassilopoulos, S., Bershadsky, A., Théry, M., & Etienne-Manneville, S., et al. Microtubules tune mechanosensitive cell responses. Nature Materials. 2020;21(3): 366–377. 10.1038/s41563-021-01108-x 34663953

[215] Etienne-Manneville S. Cytoplasmic intermediate filaments in cell biology. Annual Review of Cell and Developmental Biology. 2018;34(1):1–28. 10.1146/annurev-cellbio-100617-062534 30059630

[216] Sanghvi-Shah R, Weber GF. Intermediate filaments at the junction of mechanotransduction, migration, and development. Frontiers in Cell and Developmental Biology. 2017;5:81. 28959689 10.3389/fcell.2017.00081 PMC5603733

[217] Van Bodegraven EJ, Etienne-Manneville S. Intermediate filaments against actomyosin: the david and goliath of cell migration. Curr Opin Cell Biol. 2020;66:79–88. 32623234 10.1016/j.ceb.2020.05.006

[218] Jones JCR, Yuan Kam C, Harmon RM, et al. Intermediate filaments and the plasma membrane. Cold Spring Harb Perspect Biol. 2017;9(1):a025866. 28049646 10.1101/cshperspect.a025866 PMC5204322

[219] Nava MM, Miroshnikova YA, Biggs LC, et al. Heterochromatin-driven nuclear softening protects the genome against mechanical stress-induced damage. Cell. 2020;181(4):800–817.e22. 32302590 10.1016/j.cell.2020.03.052 PMC7237863

[220] Sneider A, Hah J, Wirtz D, et al. Recapitulation of molecular regulators of nuclear motion during cell migration. Cell Adh Migr. 2019;13(1):50–62. 30261154 10.1080/19336918.2018.1506654 PMC6527386

[221] Calero-Cuenca FJ, Janota CS, Gomes ER. Dealing with the nucleus during cell migration. Curr Opin Cell Biol. 2018;50:35–41. 29454272 10.1016/j.ceb.2018.01.014

[222] Wang F, Chen S, Liu HB, et al. Keratin 6 regulates collective keratinocyte migration by altering cell–cell and cell–matrix adhesion. J Cell Biol. 2018;217(12):4314–4330. 30389720 10.1083/jcb.201712130 PMC6279382

[223] Schaedel L, Lorenz C, Schepers AV, et al. Vimentin intermediate filaments stabilize dynamic microtubules by direct interactions. Nat Commun. 2021;12(1):1–12. 34145230 10.1038/s41467-021-23523-z PMC8213705

[224] Doss BL, Pan M, Gupta M, et al. Cell response to substrate rigidity is regulated by active and passive cytoskeletal stress. Proc Natl Acad Sci U S A. 2020;117(23):12817–12825. 32444491 10.1073/pnas.1917555117 PMC7293595

[225] Kumari S, Mak M, Poh Y, et al. Cytoskeletal tension actively sustains the migratory T‐cell synaptic contact. EMBO J. 2020;39(5):e102783. 31894880 10.15252/embj.2019102783 PMC7049817

[226] Renkawitz J, Kopf A, Stopp J, et al. Nuclear positioning facilitates amoeboid migration along the path of least resistance. Nature. 2019;568(7753):546–550. 30944468 10.1038/s41586-019-1087-5 PMC7217284

[227] Kopf A, Renkawitz J, Hauschild R, et al. Microtubules control cellular shape and coherence in amoeboid migrating cells. J Cell Biol. 219(6):2020. 10.1083/jcb.201907154 PMC7265309 32379884

[228] Joaquina Jimenez A, Schaeffer A, De Pascalis C, et al. Acto-myosin network geometry defines centrosome position. Curr Biol. 2021. 10.1016/j.cub.2021.01.002 33609453

[229] Mathew Kalappurakkal J, Sil P, Mayor S. Toward a new picture of the living plasma membrane. Protein Sci. 2020;29(6):1355–1365. 32297381 10.1002/pro.3874 PMC7255504

[230] Barber-Pérez N, Georgiadou M, Guzmán C, et al. Mechano-responsiveness of fibrillar adhesions on stiffness-gradient gels. J Cell Sci. 2020;133(12):jcs.242909. 10.1242/jcs.242909 PMC7328166 32393601

[231] Lachowski D, Cortes E, Robinson B, et al. FAK controls the mechanical activation of YAP, a transcriptional regulator required for durotaxis. FASEB J. 2018;32(2):1099–1107. 29070586 10.1096/fj.201700721R

[232] Mason DE, Collins JM, Dawahare JH, et al. YAP and TAZ limit cytoskeletal and focal adhesion maturation to enable persistent cell motility. J Cell Biol. 2019;218(4):1369–1389. 30737263 10.1083/jcb.201806065 PMC6446844

[233] Totaro A, Panciera T, Piccolo S. YAP/TAZ upstream signals and downstream responses. Nat Cell Bio. 2018;20(8):888–899. 30050119 10.1038/s41556-018-0142-z PMC6186418

[234] Nardone G, Oliver-De La Cruz J, Vrbsky J, et al. YAP regulates cell mechanics by controlling focal adhesion assembly. Nat Commun. 2017;8:15321. 28504269 10.1038/ncomms15321 PMC5440673

[235] Praful RN, Wirtz D. Enabling migration by moderation: YAP/TAZ are essential for persistent migration. J Cell Biol. 2019;218(4):1092–1093. 30872360 10.1083/jcb.201902035 PMC6446847

[236] Zanconato F, Cordenonsi M, Piccolo S. YAP and TAZ: a signalling hub of the tumour microenvironment. Nat Rev Cancer. 2019;19(8):454–464. 31270418 10.1038/s41568-019-0168-y

[237] Warren J, Xiao Y, Lamar J. YAP/TAZ activation as a target for treating metastatic cancer. Cancers (Basel). 2018;10(4):115. 10.3390/cancers10040115 PMC5923370 29642615

[238] Shen J, Cao B, Wang Y, et al. Hippo component YAP promotes focal adhesion and tumour aggressiveness via transcriptionally activating THBS1/FAK signalling in breast cancer. J Exp Clin Cancer Res. 2018;37(1):1–17. 30055645 10.1186/s13046-018-0850-z PMC6064138

[239] Panciera T, Citron A, Di Biagio D, et al. Reprogramming normal cells into tumour precursors requires ECM stiffness and oncogene-mediated changes of cell mechanical properties. Nat Mater. 2020;19(7):797–806. 32066931 10.1038/s41563-020-0615-x PMC7316573

[240] Janota CS, Javier Calero-Cuenca F, Gomes ER. The role of the cell nucleus in mechanotransduction. Curr Opin Cell Biol. 2020;63:204–211. 32361559 10.1016/j.ceb.2020.03.001

[241] Krause M, Wei Yang F, te Lindert M, et al. Cell migration through three-dimensional confining pores: speed accelerations by deformation and recoil of the nucleus. Philos Trans R Soc B Biol Sci. 2019;374(1779):20180225. 10.1098/rstb.2018.0225 PMC6627020 31431171

[242] Pyo Lee H, Alisafaei F, Adebawale K, et al. The nuclear piston activates mechanosensitive ion channels to generate cell migration paths in confining microenvironments. Sci Adv. 2021;7(2):eabd4058. 33523987 10.1126/sciadv.abd4058 PMC7793582

[243] Xia Y, Pfeifer CR, Discher DE. Nuclear mechanics during and after constricted migration. Acta Mech Sin Xuebao. 2019;35(2):299–308.

[244] Davidson PM, Battistella A, Déjardin T, et al. Nesprin‐2 accumulates at the front of the nucleus during confined cell migration. EMBO Rep. 2020;21(7):e49910. 32419336 10.15252/embr.201949910 PMC7332974

[245] Shokrollahi M, Mekhail K. Interphase microtubules in nuclear organization and genome maintenance. Trends Cell Biol. 2021;31(9): 721–731. https://www.sciencedirect.com/science/article/abs/pii/S0962892421000702 33902985 10.1016/j.tcb.2021.03.014

[246] Bouzid T, Kim E, Riehl BD, et al. The LINC complex, mechanotransduction, and mesenchymal stem cell function and fate. J Biol Eng. 2019;13(1):1–12. 31406505 10.1186/s13036-019-0197-9 PMC6686368

[247] Patricia MD, Cadot B. Actin on and around the Nucleus. Trends Cell Biol. 2021;31(3):211–223. 33376040 10.1016/j.tcb.2020.11.009

[248] Jin Heo S, Hoon Song K, Thakur S, et al. Nuclear softening expedites interstitial cell migration in fibrous networks and dense connective tissues. Sci Adv. 2020;6(25):5083–5102. 10.1126/sciadv.aax5083 PMC7304973 32596438

[249] Vortmeyer-Krause M, te Lindert M, te Riet J, et al. Lamin B2 follows lamin A/C- mediated nuclear mechanics and cancer cell invasion efficacy. bioRxiv Cell Biol. 2020. https://www.biorxiv.org/content/10.1101/2020.04.07.028969v1

[250] Dreger M, Madrazo E, Hurlstone A, et al. Novel contribution of epigenetic changes to nuclear dynamics. Nucleus. 2019;10(1):42–47. 30784352 10.1080/19491034.2019.1580100 PMC6527383

[251] Mukherjee A, Barai A, Singh RK, et al. Nuclear plasticity increases susceptibility to damage during confined migration. PLOS Comput Biol. 2020;16(10):e1008300. 33035221 10.1371/journal.pcbi.1008300 PMC7577492

[252] Ye Shiu J, Aires L, Lin Z, et al. Nanopillar force measurements reveal actin-cap-mediated YAP mechanotransduction. Nat Cell Bio. 2018;20(3):262–271. 29403039 10.1038/s41556-017-0030-y

[253] Harada T, Swift J, Irianto J, et al. Nuclear lamin stiffness is a barrier to 3D migration, but softness can limit survival. J Cell Biol. 2014;204(5):669–682. 24567359 10.1083/jcb.201308029 PMC3941057

[254] Stowers RS, Shcherbina A, Israeli J, et al. Matrix stiffness induces a tumorigenic phenotype in mammary epithelium through changes in chromatin accessibility. Nat Biomed Eng. 2019;3(12):1009–1019. 31285581 10.1038/s41551-019-0420-5 PMC6899165

[255] Pfeifer CR, Vashisth M, Xia Y, et al. Nuclear failure, DNA damage, and cell cycle disruption after migration through small pores: a brief review. Essays Biochem. 2019;63(5):569–577. 31366473 10.1042/EBC20190007

[256] Venturini V, Pezzano F, Català Castro F, et al. The nucleus measures shape changes for cellular proprioception to control dynamic cell behavior. Science. 2020;370:6514. 10.1126/science.aba2644 33060331

[257] Lomakin AJ, Cattin CJ, Cuvelier D, et al. The nucleus acts as a ruler tailoring cell responses to spatial constraints. Science. 2020;370:6514. 10.1126/science.aba2894 PMC8059074 33060332

[258] Mak M. Impact of crosslink heterogeneity on extracellular matrix mechanics and remodeling. Comput Struct Biotechnol J. 2020;18:3969–3976. 33335693 10.1016/j.csbj.2020.11.038 PMC7734217

[259] Pakshir P, Alizadehgiashi M, Wong B, et al. Dynamic fibroblast contractions attract remote macrophages in fibrillar collagen matrix. Nat Commun. 10(1):2019. 10.1038/s41467-019-09709-6 PMC6478854 31015429

[260] Kim OV, Litvinov RI, Alber MS, et al. Quantitative structural mechanobiology of platelet-driven blood clot contraction. Nat Commun. 2017;8(1):1274. 29097692 10.1038/s41467-017-00885-x PMC5668372

[261] Doyle AD, Sykora DJ, Pacheco GG, et al. 3D mesenchymal cell migration is driven by anterior cellular contraction that generates an extracellular matrix prestrain. Dev Cell. 2021. 10.1016/j.devcel.2021.02.017 PMC8082573 33705692

[262] Malandrino A, Trepat X, Kamm RD, et al. Dynamic filopodial forces induce accumulation, damage, and plastic remodeling of 3D extracellular matrices. PLOS Comput Biol. 2019;15(4):e1006684. 30958816 10.1371/journal.pcbi.1006684 PMC6472805

[263] Chang J, Chaudhuri O. Beyond proteases: basement membrane mechanics and cancer invasion. J Cell Biol. 2019;218(8):2456–2469. 31315943 10.1083/jcb.201903066 PMC6683740

[264] Wisdom KM, Adebowale K, Chang J, et al. Matrix mechanical plasticity regulates cancer cell migration through confining microenvironments. Nat Commun. 2018;9(1):1–13. 30297715 10.1038/s41467-018-06641-z PMC6175826

[265] Movilla N, Borau C, Valero C, et al. Degradation of extracellular matrix regulates osteoblast migration: a microfluidic-based study. Bone. 2018;107:10–17. 29107125 10.1016/j.bone.2017.10.025

[266] Chen Z, Jain A, Liu H, et al. Targeted drug delivery to hepatic stellate cells for the treatment of liver fibrosis. J Pharmacol Exp Ther. 2019;370(3):695–702. 30886124 10.1124/jpet.118.256156 PMC6806344

[267] Rainero E. Extracellular matrix endocytosis in controlling matrix turnover and beyond: emerging roles in cancer. Biochem Soc Trans. 2016;44(5):1347–1354. 27911717 10.1042/BST20160159

[268] Jose Thottacherry J, Joanna Kosmalska A, Kumar A, et al. Mechanochemical feedback control of dynamin independent endocytosis modulates membrane tension in adherent cells. Nat Commun. 2018;9(1):1–14. 30310066 10.1038/s41467-018-06738-5 PMC6181995

[269] Saini K, Dennis ED. Forced unfolding of proteins directs biochemical cascades. Biochemistry. 2019;58(49):4893–4902. 31736312 10.1021/acs.biochem.9b00839

[270] Saini K, Cho S, Dooling LJ, et al. Tension in fibrils suppresses their enzymatic degradation – a molecular mechanism for ‘use it or lose it’. Matrix Biol. 2020;85-86:34–46. 31201857 10.1016/j.matbio.2019.06.001 PMC6906264

[271] Nasello G, Alaman-Diez P, Schiavi J, et al. Primary human osteoblasts cultured in a 3D microenvironment create a unique representative model of their differentiation into osteocytes. Front Bioeng Biotechnol. 2020;8:336. 32391343 10.3389/fbioe.2020.00336 PMC7193048

[272] Winkler J, Abisoye-Ogunniyan A, Metcalf KJ, et al. Concepts of extracellular matrix remodelling in tumour progression and metastasis. Nat Commun. 2020;11(1):1–19. 33037194 10.1038/s41467-020-18794-x PMC7547708

[273] Chang J, Pickard A, Garva R, et al. The endosome is a master regulator of plasma membrane collagen fibril assembly. bioRxiv. 2021. https://www.biorxiv.org/content/10.1101/2021.03.25.436925v1

[274] Vogel V. Unraveling the mechanobiology of extracellular matrix. Annu Rev Physiol. 2018;80:353–387. 29433414 10.1146/annurev-physiol-021317-121312

[275] Hua Zhou Z, Cheng Dong J, Liang Xiao H, et al. Reorganized collagen in the tumor microenvironment of gastric cancer and its association with prognosis. J Cancer. 2017;8(8):1466–1476. 28638462 10.7150/jca.18466 PMC5479253

[276] Fonta CM, Arnoldini S, Jaramillo D, et al. Fibronectin fibers are highly tensed in healthy organs in contrast to tumors and virus-infected lymph nodes. Matrix Biol Plus. 2020;8:100046. 33543039 10.1016/j.mbplus.2020.100046 PMC7852196

[277] Rubina Perestrelo A, Catarina Silva A, Oliver-De La Cruz J, et al. Multiscale analysis of extracellular matrix remodeling in the failing heart. Circ Res. 2021;128(1):24–38. 33106094 10.1161/CIRCRESAHA.120.317685

[278] Calvo F, Ege N, Grande-Garcia A, et al. Mechanotransduction and YAP-dependent matrix remodelling is required for the generation and maintenance of cancer-associated fibroblasts. Nat Cell Bio. 2013;15(6):637–646. 23708000 10.1038/ncb2756 PMC3836234

[279] Fang Y, Gong H, Yang R, et al. An active biomechanical model of cell adhesion actuated by intracellular tensioning-taxis. Biophys J. 2020;118(11):2656–2669. 32380000 10.1016/j.bpj.2020.04.016 PMC7264853

[280] Elisabeth GR, Roeland MHM. Cell shape and durotaxis explained from cell-extracellular matrix forces and focal adhesion dynamics. iScience. 2020;23(9):101488. 32896767 10.1016/j.isci.2020.101488 PMC7482025

[281] Vargas DA, Gonçalves IG, Heck T, et al. Modeling of mechanosensing mechanisms reveals distinct cell migration modes to emerge from combinations of substrate stiffness and adhesion receptor–ligand affinity. Front Bioeng Biotechnol. 2020;8:459. 32582650 10.3389/fbioe.2020.00459 PMC7283468

[282] Zmurchok C, Collette J, Rajagopal V, et al. Membrane tension can enhance adaptation to maintain polarity of migrating cells. Biophys J. 2020;119(8):1617–1629. 32976760 10.1016/j.bpj.2020.08.035 PMC7642449

[283] Zheng Y, Fan Q, Eddy CZ, et al. Modeling multicellular dynamics regulated by extracellular-matrix-mediated mechanical communication via active particles with polarized effective attraction. Phys Rev E. 2020;102(5):052409. 33327171 10.1103/PhysRevE.102.052409

[284] Feng J, Levine H, Mao X, et al. Cell motility, contact guidance, and durotaxis. Soft Matter. 2019;15(24):4856–4864. 31161163 10.1039/c8sm02564a

[285] Rahman Hassan A, Biel T, Kim T. Mechanical model for durotactic cell migration. ACS Biomater Sci Eng. 2019;5(8):3954–3963. 32676537 10.1021/acsbiomaterials.8b01365 PMC7365619

[286] Thüroff F, Goychuk A, Reiter M, et al. Bridging the gap between single-cell migration and collective dynamics. Elife. 2019;8: e46842. https://elifesciences.org/articles/46842 31808744 10.7554/eLife.46842 PMC6992385

[287] Zheng Y, Nan H, Liu Y, et al. Modeling cell migration regulated by cell extracellular-matrix micromechanical coupling. Phys Rev E. 2019;100(4):043303. 31770879 10.1103/PhysRevE.100.043303

[288] Andasari V, Lü D, Swat M, et al. Computational model of wound healing: EGF secreted by fibroblasts promotes delayed re-epithelialization of epithelial keratinocytes. Integr Biol. 2018;10(10):605–634. 10.1039/c8ib00048d PMC6571173 30206629

[289] Moreira-Soares M, Cunha SP, Rafael Bordin J, et al. Adhesion modulates cell morphology and migration within dense fibrous networks. J Phys Condens Matter. 2020;32(31):314001. 32378515 10.1088/1361-648X/ab7c17

[290] Sharon Wei Ling Lee RJS, Litvak F, Spill F, et al. Integrated in silico and 3D in vitro model of macrophage migration in response to physical and chemical factors in the tumor microenvironment. Integr Biol. 2020;12(4):90–108. 10.1093/intbio/zyaa007 PMC7167463 32248236

[291] Ang L, Sun M, Spill F, et al. Are the effects of independent biophysical factors linearly additive? A 3D tumor migration model. Biophys J. 2019;117(9):1702–1713. 31630809 10.1016/j.bpj.2019.09.037 PMC6838959

[292] Van Liedekerke P, Neitsch J, Johann T, et al. A quantitative high resolution computational cell model to unravel the mechanics in living tissues. Comput Methods Biomech Biomed Eng. 2019;22(sup1):S367–S369.

[293] Winkler B, Aranson IS, Ziebert F. Confinement and substrate topography control cell migration in a 3D computational model. Commun Phys. 2019;2(1):82.

[294] Heck T, Vargas DA, Smeets B, et al. The role of actin protrusion dynamics in cell migration through a degradable viscoelastic extracellular matrix: insights from a computational model. PLOS Comput Biol. 2020;16(1):e1007250. 31929522 10.1371/journal.pcbi.1007250 PMC6980736

[295] Maxian O, Mogilner A, Strychalski W. Computational estimates of mechanical constraints on cell migration through the extracellular matrix. PLOS Comput Biol. 2020;16(8):e1008160. 32853248 10.1371/journal.pcbi.1008160 PMC7480866

[296] Zhu J, Mogilner A. Comparison of cell migration mechanical strategies in three-dimensional matrices a computational study. Interface Focus. 2016;6(5):20160040. 27708764 10.1098/rsfs.2016.0040 PMC4992743

[297] Cao Y, Ghabache E, Miao Y, et al. A minimal computational model for three-dimensional cell migration. J R Soc Interface. 16(161):2019. 10.1098/rsif.2019.0619 PMC6936042 31847757

[298] Sun M, Muhammad HHZ. Modeling, signaling and cytoskeleton dynamics: integrated modeling-experimental frameworks in cell migration. Wiley Interdiscip Rev Syst Biol Med. 2017;9(1):e1365. 10.1002/wsbm.1365 PMC5338640 27863122

[299] Shatkin G, Yeoman B, Birmingham K, et al. Computational models of migration modes improve our understanding of metastasis. APL Bioeng. 2020;4(4):41505. 10.1063/5.0023748 PMC7647620 33195959

[300] Movilla N, Valero C, Borau C, et al. Matrix degradation regulates osteoblast protrusion dynamics and individual migration. Integr Biol. 2019;11(11):404–413. 10.1093/intbio/zyz035 31922533

[301] Kim M-C, Silberberg YR, Abeyaratne R, et al. Computational modeling of three-dimensional ECM-rigidity sensing to guide directed cell migration. Proc Natl Acad Sci U S A. 2018;115(3):E390–E399. 29295934 10.1073/pnas.1717230115 PMC5776995

[302] Ribeiro FO, Gómez-Benito MJ, Folgado J, et al. Computational model of mesenchymal migration in 3D under chemotaxis. Comput Methods Biomech Biomed Eng. 2017;20(1):59–74. 10.1080/10255842.2016.1198784 PMC5061084 27336322

[303] Eric JC, Bagchi P. A computational study of amoeboid motility in 3D: the role of extracellular matrix geometry, cell deformability, and cell–matrix adhesion. Biomech Model Mechanobiol. 2021;20(1): 167–191. https://link.springer.com/article/10.1007/s10237-020-01376-7#citeas 32772275 10.1007/s10237-020-01376-7

[304] Moure A, Gomez H. Three-dimensional simulation of obstacle-mediated chemotaxis. Biomech Model Mechanobiol. 17(5):2018. 10.1007/s10237-018-1023-x 29728784

[305] Eric JC, Bagchi P. A computational model of amoeboid cell swimming. Phys Fluids. 2017;29(10):101902.

[306] Ryan JP, Gavara N, Richard SC, et al. Nonpolarized signaling reveals two distinct modes of 3D cell migration. J Cell Biol. 2012;197(3):439–455. 22547408 10.1083/jcb.201201124 PMC3341168

[307] Serrano-Alcalde F, Manuel García-Aznar J, José Gómez-Benito M. Cell biophysical stimuli in lobodopodium formation: a computer based approach. Comput Methods Biomech Biomed Eng. 2020;24(5):496–505. https://www.tandfonline.com/doi/abs/10.1080/10255842.2020.1836622 10.1080/10255842.2020.1836622 33111554

[308] Alert R, Trepat X. Physical models of collective cell migration. Annu Rev Condens Matter Phys. 2020;11(1):77–101.

[309] Camley BA, Rappel W-J. Physical models of collective cell motility: from cell to tissue. J Phys D Appl Phys. 2017;50(11):113002. 28989187 10.1088/1361-6463/aa56fe PMC5625300

[310] Deutsch A, Manik Nava-Sedeño J, Syga S, et al. BIO-LGCA: a cellular automaton modelling class for analysing collective cell migration. PLOS Comput Biol. 2021;17(6):e1009066. 34129639 10.1371/journal.pcbi.1009066 PMC8232544

[311] Garcia-Gonzalez D, Muñoz-Barrutia A. Computational insights into the influence of substrate stiffness on collective cell migration. Extrem Mech Lett. 2020;40:100928.

[312] Mayalu MN, Cheol Kim M, Harry Asada H. Multi-cell ECM compaction is predictable via superposition of nonlinear cell dynamics linearized in augmented state space. PLOS Comput Biol. 2019;15(9):e1006798. 31539369 10.1371/journal.pcbi.1006798 PMC6774565

[313] Neumann NM, Perrone MC, Veldhuis JH, et al. Coordination of receptor tyrosine kinase signaling and interfacial tension dynamics drives radial intercalation and tube elongation. Dev Cell. 2018;45(1):67–82.e6. 29634937 10.1016/j.devcel.2018.03.011 PMC5983037

[314] Escribano J, Sunyer R, Teresa Sánchez M, et al. A hybrid computational model for collective cell durotaxis. Biomech Model Mechanobiol. 2018;17(4):1–16. 10.1007/s10237-018-1010-2 29500553

[315] Moure A, Gomez H. Phase-field model of cellular migration: three-dimensional simulations in fibrous networks. Comput Methods Appl Mech Eng. 2017;320:162–197.

[316] Bellomo N, Elaiw A, Althiabi AM, et al. On the interplay between mathematics and biology. hallmarks toward a new systems biology. Phys Life Rev. 2015;12:44–64. 25529144 10.1016/j.plrev.2014.12.002

[317] Buttenschön A, Edelstein-Keshet L. Bridging from single to collective cell migration: a review of models and links to experiments. PLOS Comput Biol. 2020;16(12):e1008411. 33301528 10.1371/journal.pcbi.1008411 PMC7728230

[318] Spill F, Bakal C, Mak M. Mechanical and systems biology of cancer. Comput Struct Biotechnol J. 2018;16:237–245. 30105089 10.1016/j.csbj.2018.07.002 PMC6077126

[319] Cheng B, Lin M, Huang G, et al. Cellular mechanosensing of the biophysical microenvironment: a review of mathematical models of biophysical regulation of cell responses. Phys Life Rev. 2017;22-23:88–119. 28688729 10.1016/j.plrev.2017.06.016 PMC5712490

[320] Borau C, Kim T, Bidone T, et al. Dynamic mechanisms of cell rigidity sensing: insights from a computational model of actomyosin networks. PLoS One. 2012;7(11):e49174. 23139838 10.1371/journal.pone.0049174 PMC3489786

[321] Fatunmbi O, Bradley RP, Kutti Kandy S, et al. A multiscale biophysical model for the recruitment of actin nucleating proteins at the membrane interface. Soft Matter. 2020;16(21):4941–4954. 32436537 10.1039/d0sm00267d PMC7373224

[322] Hetmanski JHR, Jones MC, Chunara F, et al. Combinatorial mathematical modelling approaches to interrogate rear retraction dynamics in 3D cell migration. PLOS Comput Biol. 2021;17(3):e1008213. 33690598 10.1371/journal.pcbi.1008213 PMC7984637

[323] Chad MH, Andrew DS. Modeling of cell nuclear mechanics: classes, components, and applications. Cells. 2020;9(7):1623. 10.3390/cells9071623 PMC7408412 32640571

[324] Hopkins A, Brian AC. Chemotaxis in uncertain environments: hedging bets with multiple receptors. arXiv. 2020;2(4):43146.

[325] Karagöz Z, Rijns L, Dankers PYW, et al. Towards understanding the messengers of extracellular space: computational models of outside-in integrin reaction networks. Comput Struct Biotechnol J. 2021;19:303–314. 33425258 10.1016/j.csbj.2020.12.025 PMC7779863

[326] Merino-Casallo F, Gomez-Benito MJ, Juste-Lanas Y, et al. Integration of in vitro and in silico models using bayesian optimization with an application to stochastic modeling of mesenchymal 3D cell migration. Front Physiol. 2018;9:1246. 30271351 10.3389/fphys.2018.01246 PMC6142046

[327] Moure A, Gomez H. Influence of myosin activity and mechanical impact on keratocyte polarization. Soft Matter. 2020;16(22):5177–5194. 32459252 10.1039/d0sm00473a

[328] Gonzalez-Valverde I, Manuel Garcia-Aznar J. Mechanical modeling of collective cell migration: an agent-based and continuum material approach. Comput Methods Appl Mech Eng. 2018;337:246–262.

[329] Nosbisch JL, Rahman A, Mohan K, et al. Mechanistic models of PLC/PKC signaling implicate phosphatidic acid as a key amplifier of chemotactic gradient sensing. PLOS Comput Biol. 2020;16(4):e1007708. 32255775 10.1371/journal.pcbi.1007708 PMC7164671

[330] Peng L, Trucu D, Lin P, et al. A multiscale mathematical model of tumour invasive growth. Bull Math Biol. 2017;79(3):389–429. 28210916 10.1007/s11538-016-0237-2

[331] Alexander GF, James MO. Seven challenges in the multiscale modeling of multicellular tissues. WIREs Mech Dis. 2021;14(1): e1527. https://wires.onlinelibrary.wiley.com/doi/full/10.1002/wsbm.1527 35023326 10.1002/wsbm.1527 PMC11478939

[332] Gonzalez-Valverde I. *Modeling and simulation of multi-cellular systems using hybrid FEM/Agent-based approaches*. PhD thesis, University of Zaragoza, Zaragoza, 2018.

[333] Arefi SMA, Tsvirkun D, Verdier C, et al. A biomechanical model for the transendothelial migration of cancer cells. Phys Biol. 2020;17(3):036004. 32015219 10.1088/1478-3975/ab725c

[334] Hervas-Raluy S, Garcia-Aznar JM, Gomez-Benito MJ. Modelling actin polymerization: the effect on confined cell migration. Biomech Model Mechanobiol. 2019;18(4):1177–1187. 30843134 10.1007/s10237-019-01136-2 PMC6647863

[335] Moure A, Gomez H. Phase-field modeling of individual and collective cell migration. Arch Comput Methods Eng. 2019;1:3.

[336] Ahmadzadeh H, Webster MR, Behera R, et al. Modeling the two-way feedback between contractility and matrix realignment reveals a nonlinear mode of cancer cell invasion. Proc Natl Acad Sci U S A. 2017;114(9):E1617–E1626. 28196892 10.1073/pnas.1617037114 PMC5338523

[337] Banavar SP, Trogdon M, Drawert B, et al. Coordinating cell polarization and morphogenesis through mechanical feedback. PLOS Comput Biol. 2021;17(1):e1007971. 33507956 10.1371/journal.pcbi.1007971 PMC7872284

[338] Bennett RR, Pfeifer CR, Irianto J, et al. Elastic-fluid model for DNA damage and mutation from nuclear fluid segregation due to cell migration. Biophys J. 2017;112(11):2271–2279. 28591600 10.1016/j.bpj.2017.04.037 PMC5474726

[339] Mackenzie JA, Rowlatt CF, H R. Insall. A conservative finite element ALE scheme for mass-conserving reaction-diffusion equations on evolving two-dimensional domains. arXiv. 2019;43(1):132–166.

[340] Serrano-Alcalde F, Manuel García-Aznar J, José Gómez-Benito M. The role of nuclear mechanics in cell deformation under creeping flows. J Theor Biol. 2017;432:25–32. 28802825 10.1016/j.jtbi.2017.07.028

[341] Ghaffarizadeh A, Heiland R, Friedman SH, et al. PhysiCell: an open source physics-based cell simulator for 3-D multicellular systems. PLoS Comput Biol. 2018;14(2):e1005991. 29474446 10.1371/journal.pcbi.1005991 PMC5841829

[342] Coakley S, Gheorghe M, Holcombe M, et al. Exploitation of high performance computing in the FLAME agent-based simulation framework. *Proceedings of the 14th IEEE International Conference on High Performance Computing and Communications, HPCC-2012 - 9th IEEE International Conference on Embedded Software and Systems, ICESS-2012*, Liverpool, UK. 2012:538–545. https://ieeexplore.ieee.org/abstract/document/6332218

[343] Feng S, Zhou L, Zhang Y, et al. Mechanochemical modeling of neutrophil migration based on four signaling layers, integrin dynamics, and substrate stiffness. Biomech Model Mechanobiol. 2018;17(6):1611–1630. 29968162 10.1007/s10237-018-1047-2

[344] James WR, Keith JG. An agent-based discrete collagen fiber network model of dynamic traction force-induced remodeling. J Biomech Eng. 2018;140(5):051003. 10.1115/1.4037947 28975252

[345] Drasdo D, Buttenschön A, Liedekerke P. Agent-based lattice models of multicellular systems. In: Numer. methods adv. simul. biomech. biol. process. Vol. 12. Elsevier Ltd; 2018. p. 223–238.

[346] Liedekerke P, Buttenschön A, Drasdo D. off-lattice agent-based models for cell and tumor growth. in: numer. methods adv. simul. biomech. biol. process. Vol. 14. Elsevier Ltd; 2018. p. 245–267.

[347] Letort G, Montagud A, Stoll G, et al. PhysiBoSS: a multi-scale agent-based modelling framework integrating physical dimension and cell signalling. Bioinformatics. 2019;35(7):1188–1196. 30169736 10.1093/bioinformatics/bty766 PMC6449758

[348] Ines GG, Manuel Garcia Aznar J. Extracellular matrix density regulates the formation of tumour spheroids through cell migration. PLOS Comput Biol. 2021;17(2):e1008764. 33635856 10.1371/journal.pcbi.1008764 PMC7968691

[349] Gonzalez-Valverde I, Manuel Garcia-Aznar J. An agent-based and FE approach to simulate cell jamming and collective motion in epithelial layers. Comput Part Mech. 2019;6(1):85–96.

[350] Macnamara CK, Caiazzo A, Ramis-Conde I, et al. Computational modelling and simulation of cancer growth and migration within a 3D heterogeneous tissue: the effects of fibre and vascular structure. J Comput Sci. 2020;40:101067.

[351] Sfakianakis N, Madzvamuse A, Chaplain MAJ. A hybrid multiscale model for cancer invasion of the extracellular matrix. Multiscale Model Simul. 2020;18(2):824–850.

[352] Hauser AS, Kooistra AJ, Munk C, et al. GPCR activation mechanisms across classes and macro/microscales. Nat Struct Mol Biol. 2021;28(11):879–888. 34759375 10.1038/s41594-021-00674-7 PMC8580822

[353] Bagci H, Sriskandarajah N, Robert A, et al. Mapping the proximity interaction network of the Rho-family GTPases reveals signalling pathways and regulatory mechanisms. Nat Cell Bio. 2020;22(1):120–134. 31871319 10.1038/s41556-019-0438-7

[354] Rausch V, Carsten GH. The hippo pathway, YAP/TAZ, and the plasma membrane. Trends Cell Biol. 2020;30(1):32–48. 31806419 10.1016/j.tcb.2019.10.005

[355] Narumiya S, Thumkeo D. Rho signaling research: history, current status and future directions. FEBS Lett. 2018;592(11):1763–1776. 29749605 10.1002/1873-3468.13087 PMC6032899

[356] Swaminathan V, Gloerich M. Decoding mechanical cues by molecular mechanotransduction. Curr Opin Cell Biol. 2021;72:72–80. 34218181 10.1016/j.ceb.2021.05.006

[357] Yang Y, Dong L, Chao X, et al. Insall, and Huaqing Cai. Leep1 interacts with PIP3 and the Scar/WAVE complex to regulate cell migration and macropinocytosis. J Cell Biol. 220(7):2021. 10.1083/jcb.202010096 PMC8127007 33978708

[358] Moujaber O, Stochaj U. The cytoskeleton as regulator of cell signaling pathways. Trends Biochem Sci. 2020;45(2):96–107. 31812462 10.1016/j.tibs.2019.11.003

[359] Salomaa SI, Miihkinen M, Kremneva E, et al. Conformational dynamics regulate SHANK3 actin and rap1 binding. bioRxiv. 2020.

[360] Boeynaems S, Alberti S, Fawzi NL, et al. Protein phase separation: a new phase in cell biology. Trends Cell Biol. 2018;28(6):420–435. 29602697 10.1016/j.tcb.2018.02.004 PMC6034118

[361] Alberti S. Phase separation in biology. Curr Biol. 2017;27(20):R1097–R1102. 29065286 10.1016/j.cub.2017.08.069

[362] de Curtis I. Biomolecular condensates at the front: cell migration meets phase separation. Trends Cell Biol. 2021;31(3):145–148. 33397597 10.1016/j.tcb.2020.12.002

[363] Sarkar A, LeVine DN, Kuzmina N, et al. Cell migration driven by self-generated integrin ligand gradient on ligand-labile surfaces. Curr Biol. 2020;30(20):4022–4032.e5. 32916117 10.1016/j.cub.2020.08.020 PMC7578120

[364] Elosegui-Artola A, Oria R. Cell migration: deconstructing the matrix. Curr Biol. 2020;30(20):R1266–R1268. 33080198 10.1016/j.cub.2020.08.013

[365] Shellard A, Mayor R. Durotaxis: the hard path from in vitro to in vivo. Dev Cell. 2020;56(2):227–239. 33290722 10.1016/j.devcel.2020.11.019

[366] Allen GM, Chun Lee K, Barnhart EL, et al. Cell mechanics at the rear act to steer the direction of cell migration. Cell Syst. 2020;11(3):286–299.e4. 32916096 10.1016/j.cels.2020.08.008 PMC7530145

[367] Carlos FG, Gasperini L, Alexandra PM, et al. The stiffness of living tissues and its implications for tissue engineering. Nat Rev Mater. 2020;5(5):351–370.

[368] Hapach LA, Carey SP, Schwager SC, et al. Phenotypic heterogeneity and metastasis of breast cancer cells. Cancer Res. 2021;81(13):3649–3663. 33975882 10.1158/0008-5472.CAN-20-1799 PMC9067366

[369] Turnham DJ, Yang WW, Davies J, et al. Bcl-3 promotes multi-modal tumour cell migration via NF-*κ*B1 mediated regulation of Cdc42. Carcinogenesis. 2020;41(10):1432–1443. 31957805 10.1093/carcin/bgaa005

[370] Bai J, Haase K, Roberts JJ, et al. A novel 3D vascular assay for evaluating angiogenesis across porous membranes. Biomaterials. 2021;268:120592. 33348261 10.1016/j.biomaterials.2020.120592

[371] Loebel C, Mauck RL, Burdick JA. Local nascent protein deposition and remodelling guide mesenchymal stromal cell mechanosensing and fate in three-dimensional hydrogels. Nat Mater. 2019;18(8):883–891. 30886401 10.1038/s41563-019-0307-6 PMC6650309

[372] Phillip JM, Zamponi N, Phillip MP, et al. Fractional re-distribution among cell motility states during ageing. Commun Biol. 2021;4(1):81. 33469145 10.1038/s42003-020-01605-w PMC7815872

[373] Mosier JA, Yusheng W, Reinhart-King CA. Recent advances in understanding the role of metabolic heterogeneities in cell migration. Fac Rev. 2021;10. 10.12703/r/10-8 PMC7894266 33659926

[374] Heming G, Tian M, Pei Q, et al. Extracellular matrix stiffness: new areas affecting cell metabolism. Front Oncol. 2021;11:8. 10.3389/fonc.2021.631991 PMC7943852 33718214

[375] Mosier JA, Schwager SC, Boyajian DA, et al. Cancer cell metabolic plasticity in migration and metastasis. Clin Exp Metastasis. 2021;1:3. 10.1007/s10585-021-10102-1 34076787

[376] Júlvez J, Oliver SG. Modeling, analyzing and controlling hybrid systems by Guarded Flexible Nets. Nonlinear Anal Hybrid Syst. 2019;32:131–146.

[377] Singh P, Hellander A. Surrogate assisted model reduction for stochastic biochemical reaction networks. In *Proc. - Winter Simul. Conf*. 1773–1783. Institute of Electrical and Electronics Engineers Inc., 2017.

[378] Hellander S, Hellander A. Hierarchical algorithm for the reaction-diffusion master equation. J Chem Phys. 2020;152(3):034104. 31968960 10.1063/1.5095075 PMC6964990

[379] Alber M, Buganza Tepole A, Cannon WR, et al. Integrating machine learning and multiscale modeling—perspectives, challenges, and opportunities in the biological, biomedical, and behavioral sciences. Npj Digit Med. 2019;2(1):115. 31799423 10.1038/s41746-019-0193-y PMC6877584

[380] Peter IF. Bayesian optimization. In: Recent adv. optim. model. contemp. probl. INFORMS; 2018. p. 255–278.

[381] Shahriari B, Swersky K, Wang Z, et al. Taking the human out of the loop: a review of bayesian optimization. Proc IEEE. 2016;104(1):148–175.

[382] Shields BJ, Stevens J, Jun L, et al. Bayesian reaction optimization as a tool for chemical synthesis. Nature. 2021;590(7844):89–96. 33536653 10.1038/s41586-021-03213-y

[383] Rhys Griffiths R, Miguel Hernández-Lobato J. Constrained Bayesian optimization for automatic chemical design using variational autoencoders. Chem Sci. 2020;11(2):577–586. 32190274 10.1039/c9sc04026a PMC7067240

[384] Imani M, Fatemeh Ghoreishi S. Bayesian optimization objective-based experimental design. In Proc. Am. Control Conf 2020-July, Denver, CO, USA. 3405–3411 . Institute of Electrical and Electronics Engineers Inc., 2020.https://ieeexplore.ieee.org/document/9147824

[385] Singh P, Hellander A. Hyperparameter optimization for approximate Bayesian computation. In Proc. - Winter Simul. Conf 2018-Dec, Gothenburg, Sweden. 1718–1729. Institute of Electrical and Electronics Engineers Inc., 2019. https://ieeexplore.ieee.org/abstract/document/8632304/

[386] Karagiannis G, Konomi BA, Lin G. On the Bayesian calibration of expensive computer models with input dependent parameters. Spat Stat. 2019;34:100258.

[387] Mark C, Metzner C, Lautscham L, et al. Bayesian model selection for complex dynamic systems. Nat Commun. 2018;9(1):1803. 29728622 10.1038/s41467-018-04241-5 PMC5935699

[388] Boquet-Pujadas A, Christophe Olivo-Marin J, Guillén N. Bioimage analysis and cell motility. Patterns. 2021;2(1):100170. 33511365 10.1016/j.patter.2020.100170 PMC7815951

[389] Jacquemet G, Carisey AF, Hamidi H, et al. The cell biologist’s guide to super-resolution microscopy. J Cell Sci. 133(11):2020. 10.1242/jcs.240713 32527967

[390] Pelt DM. Deep Learning: Tackling the challenges of bioimage analysis. Elife. 2020;9:e64384. https://elifesciences.org/articles/64384 33264089 10.7554/eLife.64384 PMC7710355

[391] Vicar T, Balvan J, Jaros J, et al. Cell segmentation methods for label-free contrast microscopy: review and comprehensive comparison. BMC Bioinformatics. 2019;20(1):360. 31253078 10.1186/s12859-019-2880-8 PMC6599268

[392] Khadangi A, Boudier T, Rajagopal V. EM-stellar: benchmarking deep learning for electron microscopy image segmentation. Bioinformatics. 2021;37(1): 97–106. https://academic.oup.com/bioinformatics/article/37/1/97/6069571?login=true 10.1093/bioinformatics/btaa1094 PMC8034537 33416852

[393] Lucas AM, Ryder PV, Bin L, et al. Open-source deep-learning software for bioimage segmentation. Mol Biol Cell. 2021;32(9):823–829. 33872058 10.1091/mbc.E20-10-0660 PMC8108523

[394] von Chamier L, Laine RF, Jukkala J, et al. Democratising deep learning for microscopy with ZeroCostDL4Mic. Nat Commun. 2021;12(1):2276. 33859193 10.1038/s41467-021-22518-0 PMC8050272

[395] Meijering E. A bird’s-eye view of deep learning in bioimage analysis. Comput Struct Biotechnol J. 2020;18:2312–2325. 32994890 10.1016/j.csbj.2020.08.003 PMC7494605

[396] Eisenstein M. Smart solutions for automated imaging. Nat Methods. 2020;17(11):1075–1079. 33077968 10.1038/s41592-020-00988-2

[397] McDole K, Guignard L, Amat F, et al. In toto imaging and reconstruction of post-implantation mouse development at the single-cell level. Cell. 2018;175(3):859–876.e33. 30318151 10.1016/j.cell.2018.09.031

[398] Jiaye H, Huisken J. Image quality guided smart rotation improves coverage in microscopy. Nat Commun. 2020;11(1):1–9. 31919345 10.1038/s41467-019-13821-y PMC6952408

[399] Mahecic D, Gambarotto D, Douglass KM, et al. Homogeneous multifocal excitation for high-through-put super-resolution imaging. Nat Methods. 2020;17(7):726–733. 32572233 10.1038/s41592-020-0859-z

[400] Nehme E, Freedman D, Gordon R, et al. DeepSTORM3D: dense 3D localization microscopy and PSF design by deep learning. Nat Methods. 2020;17(7):734–740. 32541853 10.1038/s41592-020-0853-5 PMC7610486

[401] Durand A, Wiesner T, André Gardner M, et al. A machine learning approach for online automated optimization of super-resolution optical microscopy. Nat Commun. 2018;9(1):1–16. 30531817 10.1038/s41467-018-07668-y PMC6286316

[402] Yichen W, Rivenson Y, Wang H, et al. Three-dimensional virtual refocusing of fluorescence microscopy images using deep learning. Nat Methods. 2019;16(12):1323–1331. 31686039 10.1038/s41592-019-0622-5

[403] Wang H, Rivenson Y, Jin Y, et al. Deep learning enables cross-modality super-resolution in fluorescence microscopy. Nat Methods. 2019;16(1):103–110. 30559434 10.1038/s41592-018-0239-0 PMC7276094

[404] Weigert M, Schmidt U, Boothe T, et al. Content-aware image restoration: pushing the limits of fluorescence microscopy. Nat Methods. 2018;15(12):1090–1097. 30478326 10.1038/s41592-018-0216-7

